# Photoactivated probiotic micro-reactor synchronizes STING/TLRs agonists to spatiotemporally synergize antitumor immunotherapy

**DOI:** 10.1186/s12951-026-04300-w

**Published:** 2026-04-02

**Authors:** Yuzhi Qiu, Yunting Liu, Sihan Chen, Yidi Liu, Xi Yu, Xiangliang Yang, Yan Zhang, Yanhong Zhu

**Affiliations:** 1 National Engineering Research Center for Nanomedicine, Huazhong University of Science and Technology, 1037 Luoyu Road, Wuhan, 430074 P. R. China; 2 Hubei Key Laboratory of Bioinorganic Chemistry and Materia Medica, Huazhong University of Science and Technology, 1037 Luoyu Road, Wuhan, 430074 P. R. China; 3 College of Life Science and Technology, Huazhong University of Science and Technology, 1037, Luoyu Road, Wuhan, 430074 P. R. China

**Keywords:** Engineered bacteria, Optogenetics, Photolabile STING prodrug, Cancer immunotherapy

## Abstract

Reprogramming tumor-associated macrophages (TAMs) from the pro-tumoral M2-like state to the immunostimulatory M1-like phenotype has emerged as a promising strategy for tumor therapy. However, most M2-like TAMs are preferentially located in hypoxic regions of the tumor, which are poorly accessible to many advanced drug delivery systems, posing a significant challenge to effective TAM reprogramming. Here, leveraging the tropism of facultative anaerobic bacteria to localize and propagate in the hypoxic tumor, an optogenetically engineered *Escherichia coli* Nissle 1917 strain conjugated with murine STING agonist (EcN_flaB_@UPD) was developed for cancer-specific immunotherapy. Upon near-infrared light illumination, the blue and UV emissions from upconversion nanoparticles (UCNPs) simultaneously activate the expression of Toll-like receptor (TLR) agonist, flaB, from EcN_flaB_, and the release of photocaged murine STING agonist, DMXAA, respectively. This spatiotemporally synchronized dual release ensures co-localized STING and TLR5 agonists inside the hypoxic niche, repolarizing TAMs from the M2 to the M1 phenotype via synergistic TLR5-MAPK1-NF-κB and STING-NF-κB signaling. The polarization of TAMs enhances their antigen-presenting capacity and, more importantly, activates the cytotoxic, stem-like and memory CD8^+^ T cells responses. This subsequently inhibits tumor growth, relapse, and metastasis in the murine 4T1 tumor model. Collectively, our work introduces the bacteria-based system that uses near-infrared light to dual-release immunotherapeutics for systemic anti-tumor immunity, opening new avenues for precise and effective cancer immunotherapy.

**Graphical abstract:**

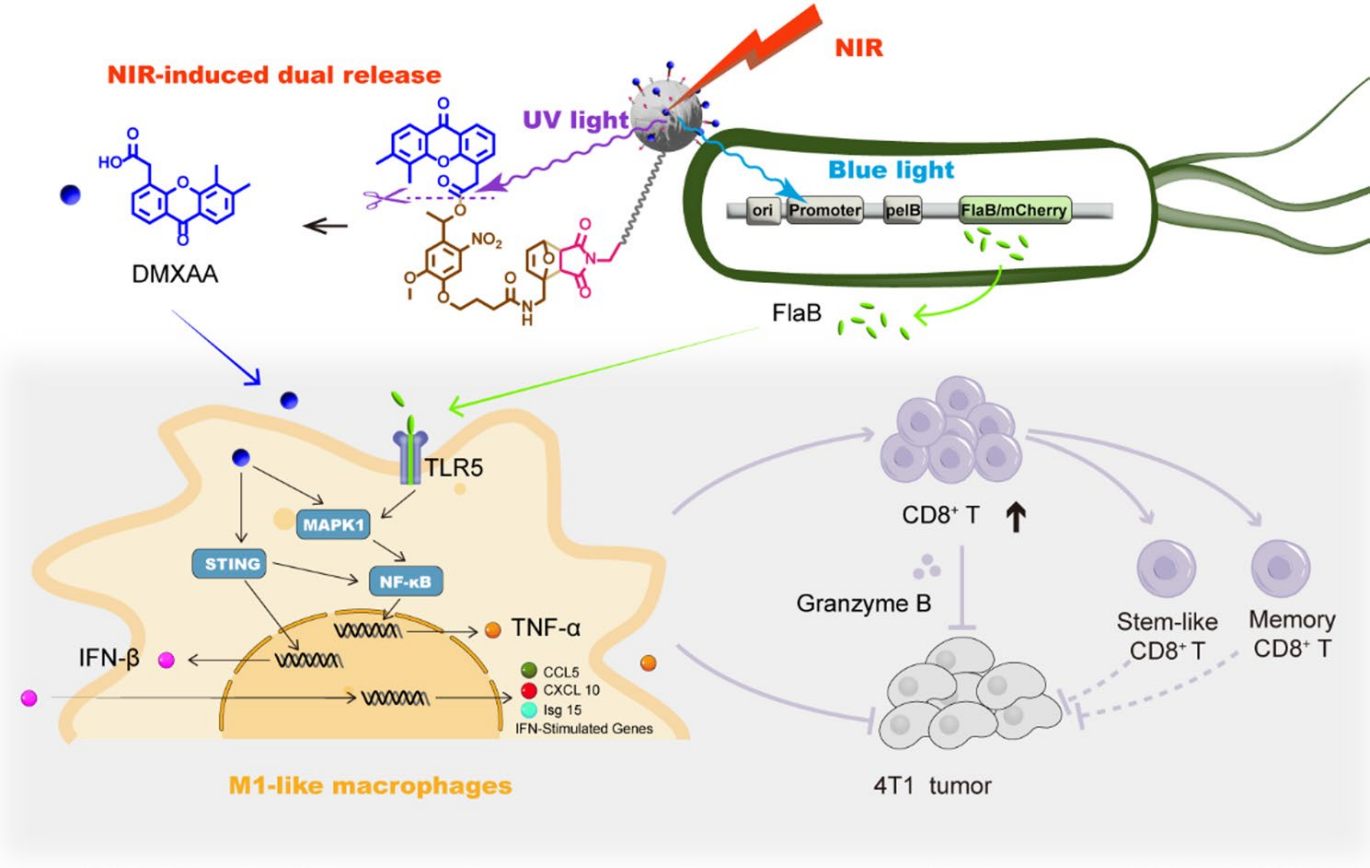

**Supplementary Information:**

The online version contains supplementary material available at 10.1186/s12951-026-04300-w.

## Introduction

The tumor immunosuppressive microenvironment (TIME) presents a key barrier to effective immunotherapy [1]. Within the TIME, tumor-associated macrophages (TAMs) are the most abundant innate immune cells that play a complex, dual role. Their function evolves from suppressing tumor growth in the early stages of cancer initiation to promoting the tumor progression and metastasis as the disease advances. This functional shift is characterized by a transition from a pro-inflammatory M1-like phenotype to an immunosuppressive M2-like phenotype in response to tumor-specific signals [2]. Considering that the high level of M2-like TAMs correlates with poor patients prognosis, repolarizing the M2-like TAMs toward the M1-like phenotype represents a promising therapeutic strategy to reverse TIME and potentiate the efficacy of immunotherapy [2–6]. However, M2-like TAMs preferentially reside in tumor hypoxic regions [3–5], which are difficult for current immunotherapies to reach due to poor penetration into hypoxic core [6]. Although nanodrugs have enhanced targeted delivery, their efficacy remains hindered by the tumor’s aberrant microenvironment—including chaotic vasculature, elevated interstitial pressure, and excessive neovascularization [7]. Thus, overcoming these barriers to access the tumor hypoxic core is essential for improving therapeutic outcomes, yet it continues to pose a major challenge.

Certain obligate or facultative anaerobic bacteria exhibit an intrinsic tropism for the hypoxic, nutrient-rich tumor core that systemically administered drugs barely penetrate [8]. By leveraging these microbes as living vectors, therapeutic payloads can be directly delivered into the deepest, hypoxic niches of solid tumors [9, 10]. Moreover, with the advancement of synthetic biology, genetically-engineered bacteria can be designed as a promising in situ drug factory, providing a steady and localized supply of peptide therapeutics [11–13]. To achieve precise therapy, genetically-engineered bacteria can be designed as environment-responsive expression systems activated by external or internal stimuli such as responsive to heat [14, 15], hypoxia [16, 17], magnetism [18, 19], and light [19, 20]. Among these, light is a particularly attractive inducer due to its capability to instantaneously and reversibly control gene expression with high spatial precision, thereby simplifying the localized release of therapeutics [21]. Light-switchable transcription molecules, such as EL222, have been demonstrated the capability for fine-tuning gene expression in a variety of organisms [22]. Although producing and releasing peptide therapeutics by the environment-responsive genetically-engineered bacteria are highly controllable, the single functionality and compromised therapeutic effects markedly limit their applications. To overcome this limitation, concurrent bacterial delivery of multiple therapeutic payloads has been shown to enhance intratumoral accumulation and therapeutic outcomes beyond what is achieved with monotherapy based on a single engineered strain [23]. Nevertheless, a fundamental obstacle persists: premature drug leakage during systemic delivery can produce off-target toxicity in healthy organs, underscoring the need for more robust containment strategies.

Prodrugs are inactive drug derivatives that convert into the active drug within the tumor in response to the endogenous or exogenous stimulus. They hold great potential for minimizing drug leakage and off-target effects [24]. A significant challenge, however, is that biopharmaceutical and pharmacodynamic barriers often prevent these prodrugs from accumulating effectively at the tumor site [25]. Obligate or facultative anaerobes naturally colonizing hypoxic tumor cores can function as living prodrug vectors, bypassing the delivery bottleneck that limits conventional prodrug strategies. Once these prodrug-armed bacteria have accumulated within the tumor, the next critical hurdle is achieving synchronized and dosage-controlled conversion to the active agents [26]. Spatially directed photolysis, particularly light-triggered cleavage of prodrug-bacteria conjugates offers a precise, on-demand solution. Independently, optogenetic circuits can be engineered into the same bacterial vector so that blue-light exposure drives de-novo synthesis of a therapeutic protein [27]. Therefore, we propose that a single light pulse can arm the system twice: liberating active drug from the prodrug and inducing in situ production of a biologic agent for re-educating M2 macrophages toward M1 phenotype. This strategy simplifies workflow, tightens spatiotemporal control, and maximizes synergistic effects within the illuminated tumor site. Notably, such a unified optical trigger has not yet been explored for bacterial-mediated combination immunotherapy.

**Fig. 1:**
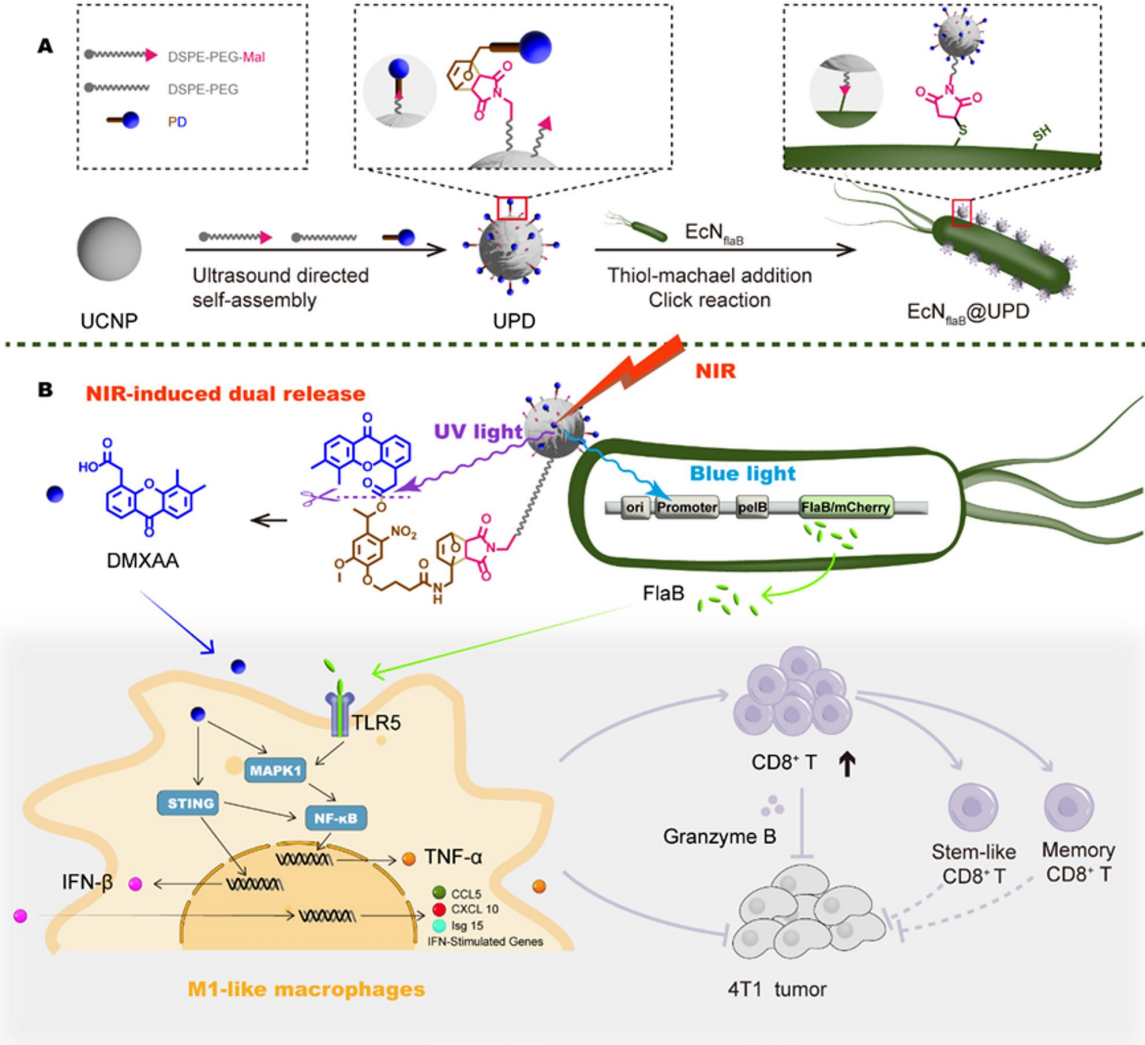
Schematic representation of optogenetics-engineered EcN conjugation with photolabile STING prodrug (EcN_flaB_@UPD) for tumor specific immunotherapy under 808 nm irradiation. **A**) The preparation of EcN_flaB_@UPD. **B**) Mechanism for simultaneous release of DMXAA and flaB from EcN_flaB_@UPD upon 808 nm irradiation. EcN_flaB_@UPD mediates the repolarization of TAMs from M2 to M1-like macrophage, and subsequent activation of cytotoxic, stem-like and memory CD8^+^ T lymphocytes in the 4T1 tumor microenvironment

Herein, we report the dually photo-responsive STING-agonists-engineered bacteria conjugates for tumor-specific immunotherapy. In this design, an upconversion nanoparticle-based prodrug-loaded genetically engineered bacterial platform (EcN_flaB_@UPD) was developed (Fig. 1). This platform utilizes genetically engineered bacteria that specifically express Flagellin B (flaB) upon blue light induction (termed EcN_flaB_). A murine STING agonist, DMXAA, was synthesized as a UV light-cleavable prodrug and loaded onto the UCNPs (termed UPD). UPD was conjugated to the surface of EcN_flaB_ through a thiol-michael addition click reaction between maleimide and thiol groups. Due to the anaerobic targeting of EcN, the prodrugs are piggybacked on bacteria to be delivered to the tumor site. Upon near-infrared illumination at the tumor site, the UCNPs trigger local UV and blue light emission, inducing DMXAA release and flaB expression, respectively. DMXAA and flaB collaboratively inhibit 4T1 tumor growth, relapse and metastasis through the polarized M1-type macrophages boosting the antitumor stem-like and memory CD8^+^ T cells.

## Results and discussion

### Design and characterization of EcN_flaB_@UPD

The synthesis of UPD is described in Figure S1 (Supporting Information). The photosensitive prodrug of DMXAA (PD) was synthesized by coupling DMXAA with o-nitrobenzyl-like photoresponsive groups via an esterification reaction [28]. The results of NMR and FT-MS spectroscopy showed the PD were successfully synthesized (Figures S2-5). The oleic acid-coated UCNPs (NaYF_4_:Yb, Tm@NaYbF_4_:Nd@NaGdF_4_) were prepared by a hydrothermal method according to our previous report [29]. Then the obtained UCNPs were modified with DSPE-PEG and DSPE-PEG2000-Mal (termed U). Thereafter, the PD was loaded on the U (termed UPD) via a click reaction between furan on the PD and maleimide on U.

**Fig. 2:**
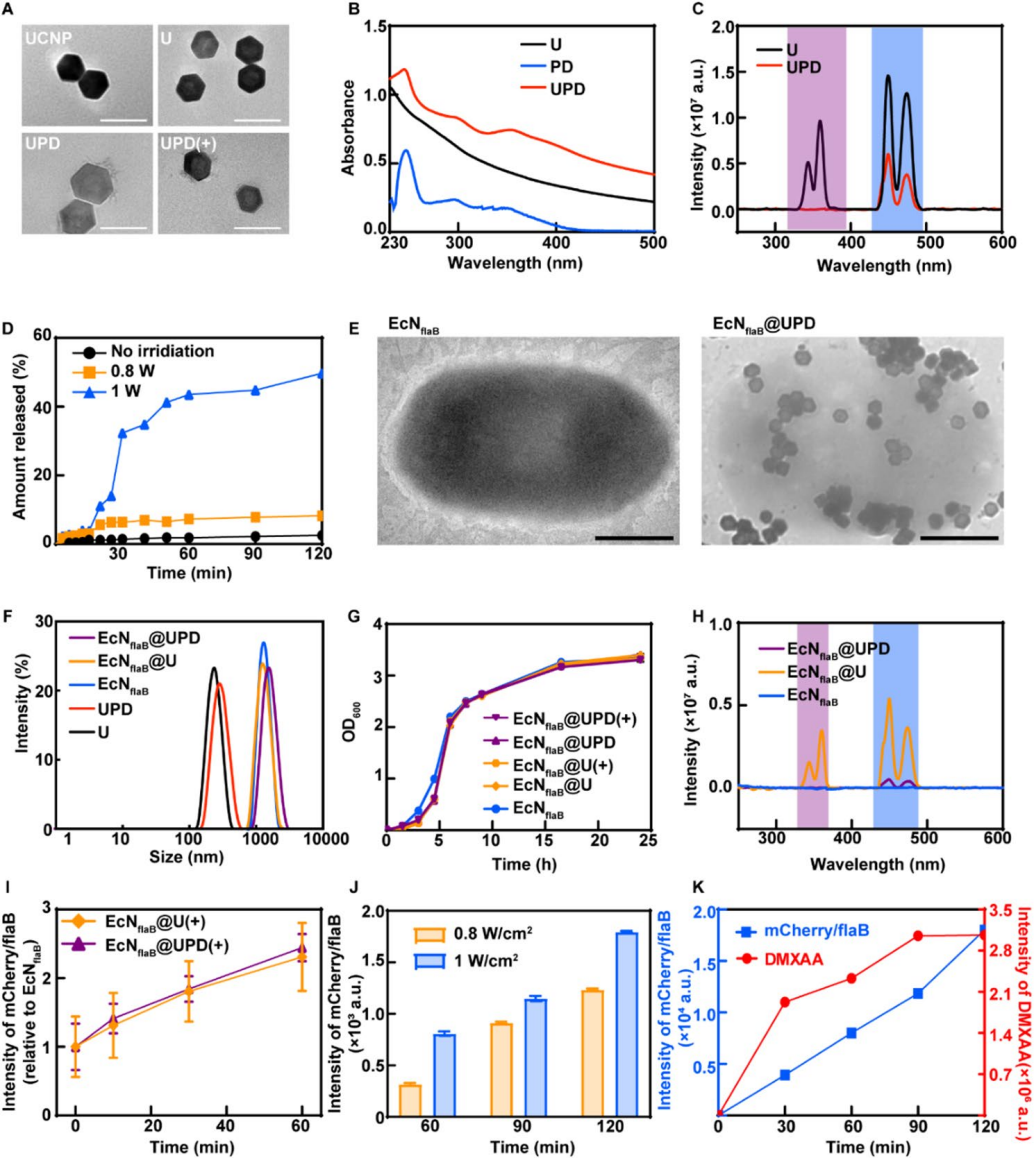
The characterization of EcN_flaB_@UPD. **A**) The TEM images of UCNP, U, UPD, and UPD after irradiation with 808 nm NIR light (UPD(+)). Scale bar = 100 nm. **B**) UV absorption diagrams of U, PD, and UPD. **C**) Upconversion luminescence of U and UPD under 808 nm NIR excitation. **D**) Drug release behavior of UPD under 808 nm NIR irradiation with different power densities. **E**) Images of EcN_flaB_ and EcN_flaB_@UPD under TEM. Scale bar = 500 nm. **F**) Hydrated particle size of U, UPD, EcN_flaB_, EcN_flaB_@U, and EcN_flaB_@UPD. G) Bacterial growth curves of EcN_flaB_, EcN_flaB_@U, EcN_flaB_@UPD with or without NIR excitation (1 W/cm^2^, 1.5 h). **H**) Fluorescence emission spectra of EcN_flaB_@U, EcN_flaB_@UPD under 808 nm NIR laser irradiation. **I**) The fluoresce intensity of mCherry/flaB in EcN_flaB_@U and EcN_flaB_@UPD relative to EcN_flaB_ at different time points under 808 nm NIR light irradiation at 1 W/cm². **J**) The fluoresce intensity of mCherry/flaB by EcN_flaB_@UPD at different time points and power densities. **K**) The simultaneous release of mCherry/flaB and DMXAA by EcN_flaB_@UPD when exposed to 808 nm NIR at the power density of 1 W/cm^2^

Figure 2A shows the transmission electron microscopy (TEM) images of UCNP, U, UPD, and UPD after NIR illumination (UPD(+)). The UCNPs were hexagonal and remained hexagonal after loading PD. The particle size of UCNP was about 42.5 nm under TEM and increased to 56.7 nm after loading the prodrug PD. The colloidal stability of UPD was evaluated using dynamic light scattering (DLS), as shown in Figure S6. After incubation in PBS for 72 h, the UPD nanoparticles maintained their hydrodynamic diameter and polydispersity index (PDI) without significant aggregation, confirming the excellent colloidal stability of UPD under physiologically relevant conditions. Elemental mapping of Y, Yb, Nd, and Gd exhibited the core/shell/shell nanostructure of UCNP in the UPD (Figure S7). The absorption spectra of U, PD, and UPD are displayed in Fig. 2B. The U had no characteristic absorption peaks at 230–500 nm, whereas both PD and UPD had absorption peaks at 245 nm, which may be attributed to o-nitrobenzyl-like photoresponsive groups in PD and UPD. According to the emission spectra shown in Fig. 2C, when illuminated with a NIR laser (808 nm), the U showed multiple emission peaks in the UV (wavelength range: 300–400 nm) and blue light (wavelength range: 400–500 nm) regions. In contrast, after piggybacking on PD, the UV emission spectrum of UPD disappeared while remained blue light emission, which may be attributed to the UV absorption of PD. After irradiation for six minutes by UV, DMXAA was completely released (Figure S8). In an ideal scenario, the maximum DMXAA loading efficiency was calculated to be 5.5 wt%. Experimentally, the percentage of UV-released DMXAA was calculated to be 5.3 wt% through fluorescence spectrometer analysis, suggesting that the PD was almost completely loaded onto U. As shown in Fig. 2D, the DMXAA release has reached 8.2% under the irradiation of 808 nm NIR at 0.8 W/cm^2^, while 49.7% DMXAA has been released from UPD under 1 W/cm^2^ illumination within two hours (the plateau occurred at one hour after the light illumination).

UPD was decorated onto the surface of EcN_flaB_ (named EcN_flaB_@UPD) via a click reaction between the maleimide on UPD and the sulfhydryl groups on the bacteria. EcN_flaB_, a genetically-engineered *E. coli* Nissle 1917 (EcN) strain, was constructed with the ability of expressing and secreting a flaB protein in response to blue light in our previous report [30]. The TEM images of the EcN_flaB_ and EcN_flaB_@UPD were shown in Fig. 2E. The average hydrodynamic diameter of each material measured by DLS was as follows: U: 232.97 nm, UPD: 285 nm, EcN_flaB_: 1153.33 nm, EcN_flaB_@U: 1339.67 nm, and EcN_flaB_@UPD: 1244.33 nm (Fig. 2F). The zeta potential plots indicated that both PD and EcN_flaB_ had a negative zeta potential of -17.76 mV and -19.43 mV, respectively. Upon crosslinking PD onto EcN_flaB_, the zeta potential of EcN_flaB_@UPD was -28.43 mV (Figure S9). The OD_600_ values of bacteria were used to determine whether the cross-linking affected the bacterial bioactivity at different time points with or without NIR illumination. As shown in Fig. 2G, similar growth curves were found in EcN_flaB_, EcN_flaB_@U, EcN_flaB_@U(+), EcN_flaB_@UPD and EcN_flaB_@UPD(+) groups, which indicated that the cross-linking and near-infrared illumination had no affection on the growth of EcN_flaB_. Furthermore, the stability of the conjugate was evaluated by incubating EcN_flaB_@UPD in 10% mouse serum at 37 °C, which showed that over 80% of UPD nanoparticles remained bound to the bacterial surface after 12 h (Figure S10). The amount of UPD cross-linked onto EcN_flaB_ was determined by inductively coupled plasma mass spectrometry (ICP-MS). A total of 0.553 mg of UPD (containing 0.268 mg of UCNPs) were crosslinked to 10^7^ CFU of bacteria (Figure S11). Next, the fluorescence emission was determined. The blue light emission of EcN_flaB_@UPD upon 808 nm NIR excitation could be detected (Fig. 2H), indicating that cross-linking did not affect the fluorescence emission performance of UPD. The mCherry/flaB fusion protein was expressed in EcN_flaB_ in response to blue light and subsequently secreted into extracellular environments. The expression levels of flaB were quantified by measuring mCherry fluorescence intensity. As shown in Fig. 2I, the fluorescence intensity of mCherry exhibited gradual increase with the irradiation time (1 W/cm^2^, 808 nm NIR). No discernible difference in mCherry fluorescence signal was found between EcN_flaB_@U(+) and EcN_flaB_@UPD(+) groups, indicating that loading PD did not affect the protein expression of EcN_flaB_. Furthermore, the fluorescence signal of mCherry in the EcN_flaB_@UPD(+) group was further demonstrated by imaging systems (IVIS) (Figure S12). In addition, the expression levels of mCherry were correlated with NIR light power density. When the irradiation power increased from 0.8 W/cm^2^ to 1 W/cm^2^, the fluorescence intensity of mCherry/flaB in the EcN_flaB_@UPD group increased (Fig. 2J). When EcN_flaB_@UPD was exposed to 1 W/cm² irradiation, the release of DMXAA and the expression of mCherry/flaB increased over time. The release of DMXAA reached a plateau at 1.5 h (Fig. 2K). Taken together, the on-demand “one stone two birds” controllable release can be achieved through UV and blue light emitted from the UCNP in the EcN_flaB_@UPD upon 808 nm illumination, in which UV induced the photolysis of the loaded PD and blue light induced the expression of the mCherrry/flaB simultaneously.

### EcN_flaB_@UPD induced immune activation and cytotoxicity in vitro

DMXAA is a murine small-molecule stimulator of interferon genes (STING) agonist that can promote macrophage polarization to M1 via STING-NF-κB or STING-MAPK1-NF-κB through direct binding to STING proteins in the cytoplasm [31]. And flaB has been shown to activate the TLR5-MAPK1-NF-κB-dependent signaling pathway, leading to macrophage recruitment and switching the phenotype of tumor-infiltrating macrophages from an anti-inflammatory M2-like phenotype to a pro-inflammatory M1-like phenotype [32]. Therefore, we deduced that EcN_flaB_@UPD under 808 nm laser with a simultaneous release of DMXAA and expression of FlaB could exhibit a synergistic ability to promote macrophage polarization towards M1.

**Fig. 3:**
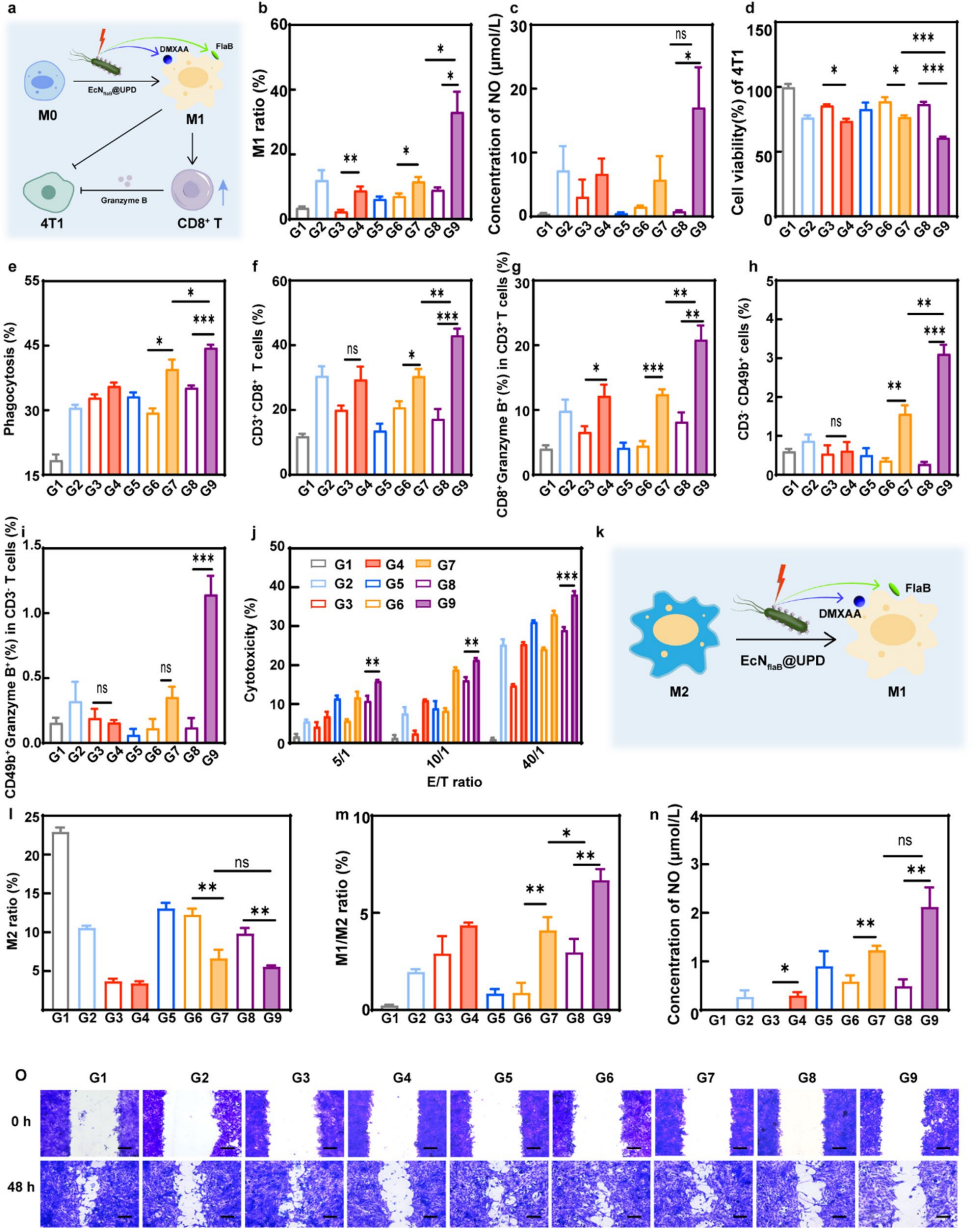
EcN_flaB_@UPD(+) mediated macrophage polarization. **A**) Schematic illustration of polarization of M0 macrophages to M1 leads to enhanced CD8^+^ T cells activation for killing 4T1 tumor cells in vitro. **B**) Quantitative analysis of the proportion of macrophages with M1 phenotype (CD86 positive) when M0 macrophages were exposed to different supernatants by flow cytometry. **C**) The content of NO in the supernatant when M0 macrophages were exposed to different material supernatants. **D**) Killing 4T1 cells by macrophage supernatants. **E**) Phagocytosis of 4T1 tumors by macrophages treated by different formulations. The ratios of CD8^+^ T cells **F**) and CD8^+^ granzyme B^+^ T lymphocytes induced by polarized macrophage **G**). **H**) and **I**) Activation of NK cells (CD3^-^CD49b^+^) mediated by polarized macrophage. **J**) Cytotoxicity of activated lymphocytes against 4T1 cells at different effector-to-target (E/T) ratios. **K**) Schematic illustration of polarization of M2 macrophages to M1. **L**) The proportion of macrophages with M1 phenotype (CD86 positive) when M2 macrophages were exposed to different treatments by flow cytometric analysis. **M**) The ratio of M1/M2 by flow cytometric analysis after different treatments. **N**) NO content in the supernatant of M2-type macrophages after different treatments. **O**) Optical microscope images of the scratch repair of migrating 4T1 cells. (4T1 cells were stained with crystal violet) Scale bar = 100 μm. G1-G9: PBS, DMXAA, UPD, UPD(+), EcN_flaB_, EcN_flaB_@U, EcN_flaB_@U(+), EcN_flaB_@UPD, and EcN_flaB_@UPD(+). *n* = 4 biologically independent samples per group. Data are means ± SEM. *P < 0.05, **P < 0.01, ***P < 0.001

Unpolarized macrophages (M0) were in a spherical state and could undergo significant morphological transformation after induction. M1-polarized macrophages possess a DC-like (a round, pancake-like) cellular morphology, while M2-type macrophages are spindle-shaped [33, 34]. To investigate whether EcN_flaB_@UPD after 808 nm illumination could promote macrophage polarization from M0-type to M1-type, the morphology changes of the RAW264.7 after incubation with supernatants of various materials were detected by a photo microscope. As shown in Figure S13 (Supporting Information), compared to the spherical morphology in the PBS group, DC-like cells could be observed in all other groups, wherein the highest proportion of DC-like morphology was found in the EcN_flaB_@UPD(+) group. To further investigate the ability of EcN_flaB_@UPD(+) to promote macrophage polarization, the expression of CD86 (a representative marker of M1-type macrophages) and CD206 (a representative marker of M2-type macrophages) was examined by flow cytometry. The ratio of M1-type macrophages was 33.27% in the EcN_flaB_@UPD(+) group, which was significantly higher than that in the UPD(+) group (9.02%) and the EcN_flaB_@U(+) group (11.85%) (Fig. 3B and Figure S14). Nitric oxide (NO) is a key nitrosative stress marker produced by M1 macrophages via iNOS [35]. The NO content in the supernatant in the EcN_flaB_@UPD(+) group was significantly higher than that in the other groups (Fig. 3C). Taken together, EcN_flaB_@UPD(+) possesses the substantial ability to promote macrophage polarization towards M1 (Fig. 3A).

Polarized M1-type macrophages secreted cytokines, including TNF-α and iNOS-derived NO, exhibit a robust antitumor capacity [36]. The viability of 4T1 tumor cells was decreased significantly after co-incubation with the supernatants of the polarized M1-type macrophages (Fig. 3D), in which, the EcN_flaB_@UPD(+) exhibited the most robust tumor cell-killing ability. When the polarized M1-type macrophages were co-incubated with 4T1 tumor cells at a 1:2 ratio, the strongest phagocytosis was found in the EcN_flaB_@UPD(+) group (Fig. 3E and Figure S15). In addition, M1-type macrophages can indirectly achieve antitumor effects by activating T cells and NK cells [37, 38]. The highest percentage of CD8^+^Granzyme B^+^ T cells and CD49b^+^Granzyme B^+^ NK cells was found in the EcN_flaB_@UPD(+) group (Fig. 3F and G, Figures S16-17; Fig. 3H and I, Figures S18-19). Furthermore, the splenic lymphocytes treated with different materials were co-incubated with 4T1 cells at 5:1, 10:1, and 40:1, showing the most pronounced 4T1 killing capacity in the EcN_flaB_@UPD(+) group (Fig. 3J).

The data above showed that EcN_flaB_@UPD(+) could stimulate un-polarized macrophages to achieve an M1 phenotype; however, tumor-associated macrophages (TAMs) are generally considered to be the predominantly M2-type, which play a pivotal role in promoting angiogenesis, cancer cell proliferation, and metastasis [33, 39]. Therefore, we investigate whether EcN_flaB_@UPD(+) was potent enough to reverse M2 macrophages into a M1 phenotype, which is beneficial for anti-cancer treatment. Here, the capacity of EcN_flaB_@UPD(+) to facilitate the reverse polarization of M2-type to M1-type macrophages was investigated (Fig. 3K). The supernatants after different treatments were used to co-incubate with M2-like macrophages (RAW264.7 pretreated by IL-4 for 48 h). Figure S20 (Supporting Information) exhibits the repolarization of M2 towards M1-type macrophages, and the highest percentage of DC-like morphology was found in the EcN_flaB_@UPD(+) group. The reprogramming of macrophages was subsequently identified by flow cytometry (Figure S21). The lowest proportion of M2-like macrophages was found in the EcN_flaB_@UPD(+) group (Fig. 3L), in which the highest M1/M2 ratio was exhibited (Fig. 3M). The highest NO content was also found in the EcN_flaB_@UPD(+) group (Fig. 3N). These findings collectively demonstrated that the EcN_flaB_@UPD promoted a shift from M2 towards M1 phenotype. M2-type macrophages were reported to promote cell proliferation and migration of breast cancer [40]. A scratch wound healing assay was carried out to evaluate the impact of supernatants from M2-repolarized macrophages on the migration of 4T1 cells within 24 h. The results revealed that the EcN_flaB_@UPD(+) strongly inhibited the migration of 4T1 cells, possessing the lowest migration rate (48.28%) compared to the PBS group (82.81%) (Fig. 3O and Figure S22).

Additionally, to evaluate the maturation of DCs induced by EcN_flaB_@UPD(+), the bone marrow dendritic cells (BMDCs) after treatments with GM-CSF and IL-4 were co-cultured with the supernatant of PBS, DMXAA, UPDMAA, UPD(+), EcN_flaB_, EcN_flaB_@U, EcN_flaB_@U(+), EcN_flaB_@UPD, and EcN_flaB_@UPD(+), and then analyzed by flow cytometry. Compared with BMDCs treated by laser- or bacteria-only, EcN_flaB_@UPD(+) can effectively induce DC maturation (Figure S23), where the percentages of mature DCs (CD11c^+^CD80^+^CD86^+^ cells) reach 20.01%. DMXAA acts as a STING agonist, also possessing vascular-disrupting function [41]. EcN_flaB_@UPD(+) significantly inhibited the migration of HUVECs by scratch assay (Figure S24A and B) and tube-forming of HUVECs by tube-forming assay (Figure S24C and D).

### The mechanism of macrophage polarization induced by EcN_flaB_@UPD(+)

**Fig. 4:**
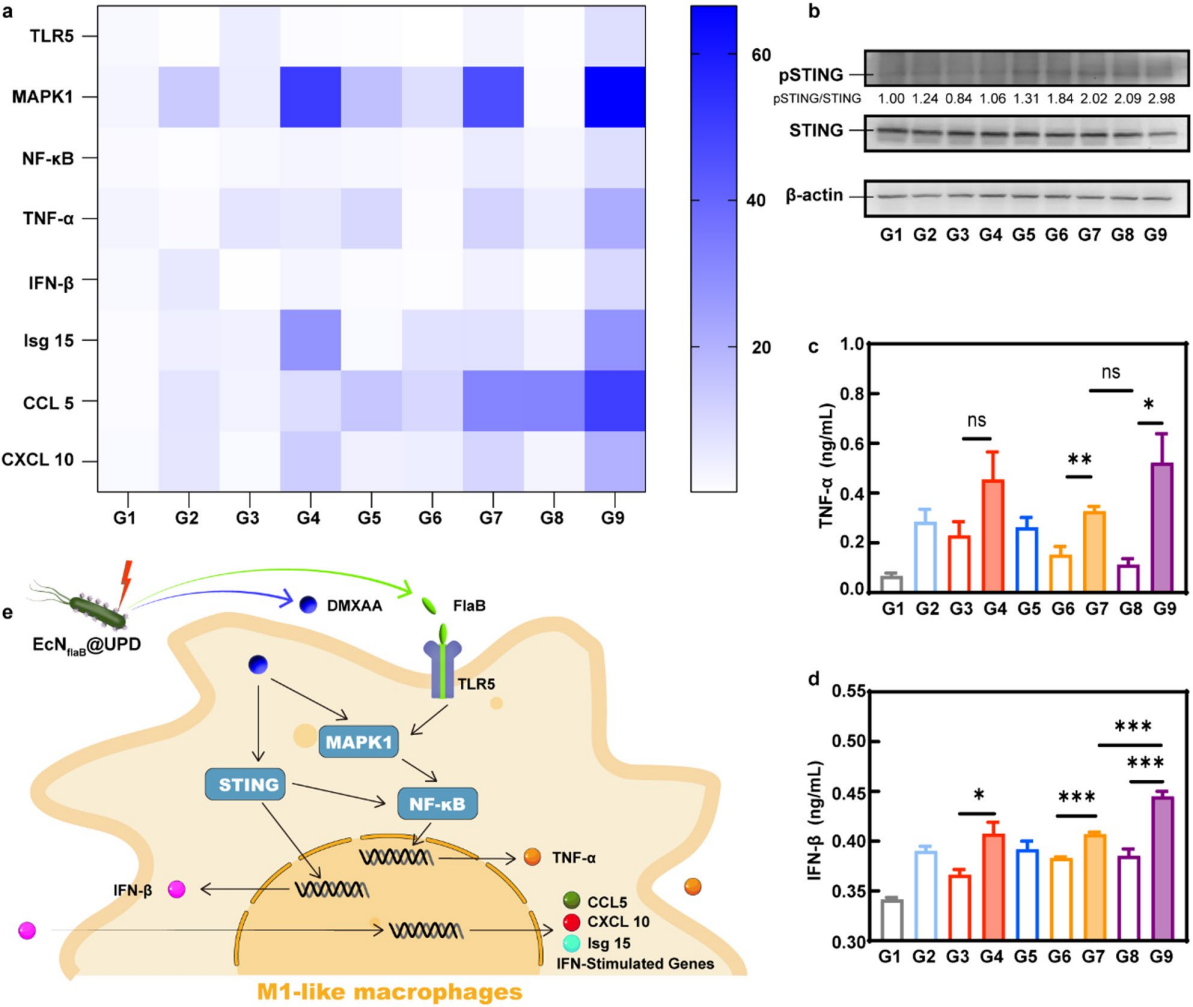
The mechanism of EcN_flaB_@UPD(+) promoting macrophage polarization. **A**) Heatmap visualization of RT-qPCR analysis of mRNA levels in the RAW264.7. GAPDH was used as a control. Data are shown as relative amounts after normalization to the mRNA level of the G1 (PBS) group. Blue represents up-regulation. Color intensity represents the relative mRNA expression values. Rows, mRNA; column, treatment. *n* = 4 biologically independent samples per group. **B**) Western blotting assay of STING, pSTING of proteins in RAW264.7. Samples derived from the same experiment and gels/blots were processed in parallel. Experiment was repeated twice independently with similar results. The concentration of **C**) TNF-α and **D**) IFN-β in the supernatants of macrophages after different treatments. *n* = 4 biologically independent samples per group. **E**) Scheme of the antitumor mechanism of EcN_flaB_@UPD(+) in vitro. G1-G9: PBS, DMXAA, UPD, UPD(+), EcN_flaB_, EcN_flaB_@U, EcN_flaB_@U(+), EcN_flaB_@UPD, and EcN_flaB_@UPD(+). Data are means ± SEM. *P < 0.05, **P < 0.01, ***P < 0.001

To elucidate the intrinsic molecular mechanism by which EcN_flaB_@UPD(+) promotes macrophage polarization towards M1, we employed fluorescence quantitative PCR to determine the expression levels of mRNA involved in the TLR5-MAPK1-NF-κB and STING pathways. The mRNA levels of TLR5, MAPK1, NF-κB, and TNF-α, which are associated with the activation of TLR5-MAPK1-NF-κB pathway, were strikingly elevated in the EcN_flaB_@UPD(+) group (Fig. 4A). The results showed that the level of IFN-β (type I interferon) was increased. The expression levels of the IFN-stimulated gene (ISG), Isg15 (an interferon-induced expression gene) [42], and CCL5 and CXCL10 (the key downstream target genes of the cGAS-STING pathway) [43], were also increased (Fig. 4A). In addition, at the protein level, the results of western blotting (Fig. 4B) showed elevations in the levels of phosphorylated STING (pSTING) in the groups containing DMXAA. And the highest amount of TNF-α (Fig. 4C) and IFN-β (Fig. 4D) in the supernatants of EcN_flaB_@UPD(+) was determined by using ELISA analysis. These findings suggest that both the TLR5-MAPK1-NF-κB and STING-NF-κB pathways can be activated by the EcN_flaB_@UPD(+), which promotes macrophages being polarized to M1-type macrophages synergistically (Fig. 4E).

Taken together, under NIR exposure, the EcN_flaB_@UPD could strongly induce macrophage polarizing towards the M1 phenotype through the synergistic activation of the TLR5-MAPK1-NF-κB and STING-NF-κB pathways.

### Antitumor efficacy and the mechanism of EcN_flaB_@UPD(+) in vivo

The excellent performance of EcN_flaB_@UPD(+) in vitro inspired us to investigate their antitumor efficacy in vivo. The in vivo distribution of EcN_flaB_@UPD stained by IR780 (IR780-EcN_flaB_@UPD) was investigated in 4T1 tumor-bearing mice. As shown in Figure S25 (Supporting Information), the fluorescence signal of EcN_flaB_@UPD gradually became stronger with time, and maintained the strong fluorescence at 48–72 h, exhibiting excellent tumor-targeting capability. Tumors and major organs were dissected to count the fluorescence values at 72 h after injection. Tumor tissues exhibited the strongest fluorescence intensity in the EcN_flaB_@UPD group. Based on the distribution results, the light irradiation began at 48 h after administration.

**Fig. 5:**
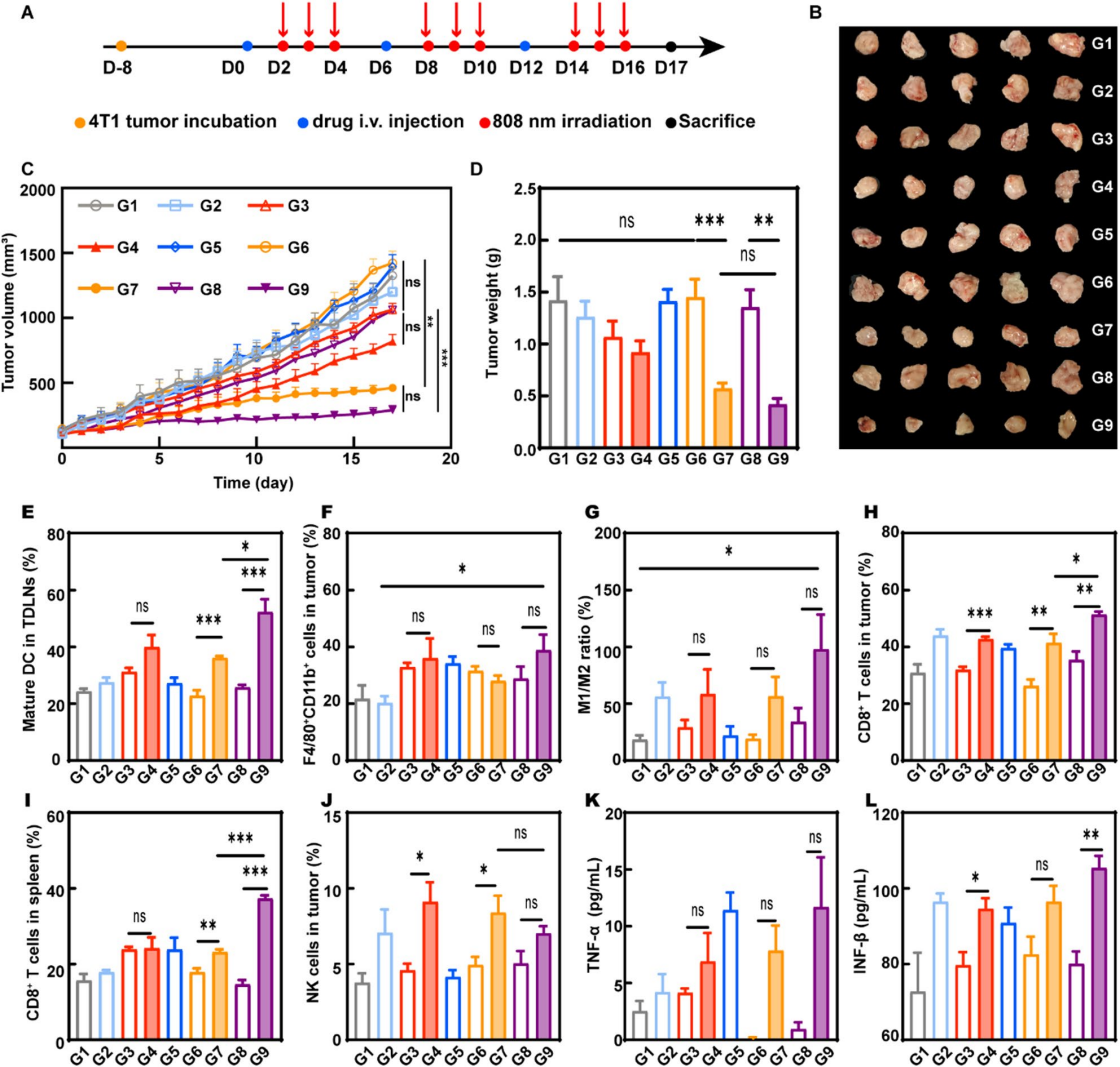
The antitumor efficacy and the mechanism in vivo. **A**) The experimental design scheme for assessing the antitumor efficacy. **B**) Representative images of the ex vivo tumor. **C**) 4T1 tumor growth curves after different treatments. (*n* = 5 animals per group) **D**) Tumor weights after different treatments. **E**) Quantitative analysis of CD80^+^ CD86^+^ on mature DCs in TDLNs by flow cytometry. (*n* = 4 animals per group) **F**) Flow cytometric quantification of the percentage of CD11b^+^ F4/80^+^ cells after treatments. (*n* = 4 animals per group) **G**) The ratio of M1/M2 in CD11b^+^F4/80^+^ cells. **H**) Quantification of CD8^+^ T cells in the tumor microenvironment. (*n* = 4 animals per group) **I**) The percentages of CD3^+^CD8^+^ T lymphocytes in spleens. **J**) The percentage of tumor-infiltrating NK cells (CD3^-^CD49b^+^). (*n* = 4 animals per group) Serum levels of **K**) TNF-α and **L**) IFN-β from mice isolated at day 17 after different treatments. (*n* = 3 animals per group) G1-G9: PBS, DMXAA, UPD, UPD(+), EcN_flaB_, EcN_flaB_@U, EcN_flaB_@U(+), EcN_flaB_@UPD, and EcN_flaB_@UPD(+). Data are presented by means ± SEM. *P < 0.05, **P < 0.01, ***P < 0.001

Thereafter, the antitumor efficacy of EcN_flaB_@UPD(+) was explored. The treatment scheme was displayed in Fig. 5A. Tumor-bearing mice were intravenously (i.v.) injected with different agents every six days, and irradiated with 808 nm NIR light (1 W cm^-2^, 15 min) at 48 h after i.v. injection for three days. The limited tumor growth inhibition effects were found in PBS, free DMXAA, UPD, EcN_flaB_, EcN_flaB_@U, and EcN_flaB_@UPD groups. UPD(+) and EcN_flaB_@U(+) could partially delay the tumor growth. In contrast, EcN_flaB_@UPD(+) exhibited the strongest inhibition of tumor growth, which may be attributed to the synergistic effect of the released flaB and DMXAA (Fig. 5B-D).

In the immune suppressive environment of solid tumors, the tumor-infiltrating DCs (TIDCs) were maintained in the immature phenotype [44]. The TIDCs’ shift towards mature phenotype was beneficial to tumor therapy. Here, the mature DCs in the tumor were analyzed by flow cytometry. Upon exposure to NIR light, EcN_flaB_@UPD could induce excellent intratumoral DC activation with a higher level of mature DC biomarkers (CD80 and CD86) compared with other groups (Figure S26). The maturation of DCs in tumor-draining lymph nodes (TDLNs) was analyzed by flow cytometry (FCM). As exhibited in Fig. 5E and Figure S27 (Supporting Information), biomarkers of mature DCs in the EcN_flaB_@UPD(+) group were significantly higher than those in the other groups. In addition, a notable surge in macrophage infiltration was found in the EcN_flaB_@UPD(+) group by flow cytometric analysis (Fig. 5F and Figure S28). M1-like TAMs in the EcN_flaB_@UPD(+) group were 1.78 times higher than those in the PBS group, while M2-polarized macrophages were 0.34 times lower than those in the PBS group (Fig. 5G and Figure S29). The M1/M2 ratio was 5.21 times higher than that in the PBS group, implying the potential of EcN_flaB_@UPD(+) to repolarize macrophages from pro-tumor M2-type to antitumor M1-type. Antigens from dead tumor cells were presented by the mature DCs and M1-type polarized macrophages towards T cells to activate T cells and promote T cell proliferation [45]. The activation of T cells in the tumor and spleen was examined by flow cytometry. Significantly, the highest CD3^+^CD8^+^ T lymphocytes were exhibited in the EcN_flaB_@UPD(+) group (Fig. 5H and I, Figure S30, and Figure S31). In addition, the population of tumor-infiltrating NK cells in the EcN_flaB_@UPD(+) group was 1.85-fold higher than that in the PBS group (Fig. 5J and Figure S32). Furthermore, the results of immunohistochemical staining showed a significantly elevated expression of Granzyme B in the tumor site in the EcN_flaB_@UPD(+) group (Figure S33). The levels of TNF-α and IFN-β produced by M1-type macrophages in different groups were examined by ELISA kits (Fig. 5K and L). The results indicated that the levels of TNF-α and IFN-β in DMXAA, EcN_flaB_, UPD(+), EcN_flaB_@U(+), and EcN_flaB_@UPD(+) groups were higher than those in the PBS group. Compared to those in the unilluminated EcN_flaB_@UPD group, the levels of TNF-α and IFN-β were significantly elevated in the EcN_flaB_@UPD(+) group, suggesting a significant activation of macrophage-mediated pathways. Given the potential risk of cytokine release syndrome (CRS) associated with such immune activation, we further measured additional pro-inflammatory cytokines (IL-6, IFN-γ, and IL-1β) in sera. Although moderate elevations were observed compared to control mice (1.3-fold for IL-6, 1.5-fold for IFN-γ, and 0.84-fold for IL-1β) (Figure S34), these levels remained within a safe range and were substantially lower than those typically associated with severe CRS [46–48].

In addition, tumor tissues were collected for TUNEL (staining apoptotic cells) and H&E staining. The number of TUNEL-positive cells was significantly higher in the EcN_flaB_@UPD(+) group compared to the other groups, which indicated the maximum degree of cell apoptosis in the EcN_flaB_@UPD(+) group (Figure S35). A similar result can be found in H&E staining (Figure S36). DMXAA has the ability to destroy tumor blood vessels [49]. CD31 is a specific marker for blood vessels [50]. The destruction of tumor blood vessels after treatments was explored by detecting the expression of CD31. The lower CD31 signal intensities were found in DMXAA, UPD(+), EcN_flaB_@U(+), and EcN_flaB_@UPD(+) groups, which may be attributed to their strong disturbing ability to tumor vasculature (Figure S37).

### The antitumor recurrence efficacy of EcN_flaB_@UPD(+)

Surgery is the preferred therapeutic method in most solid cancers without metastasis. Although surgical excision for the primary tumor can save or extend life, the surgical insult itself may precipitate or accelerate the tumor recurrence and metastasis [51]. For example, inevitable local inflammation after surgery recruits a large number of M2-like TAMs, which will promote immune escape and survival of the residual tumor cells [52]. According to the robust capability to polarize macrophages to the M1 phenotype, the potential of EcN_flaB_@UPD(+) was explored to prevent tumor recurrence and metastasis after surgical removal.

**Fig. 6:**
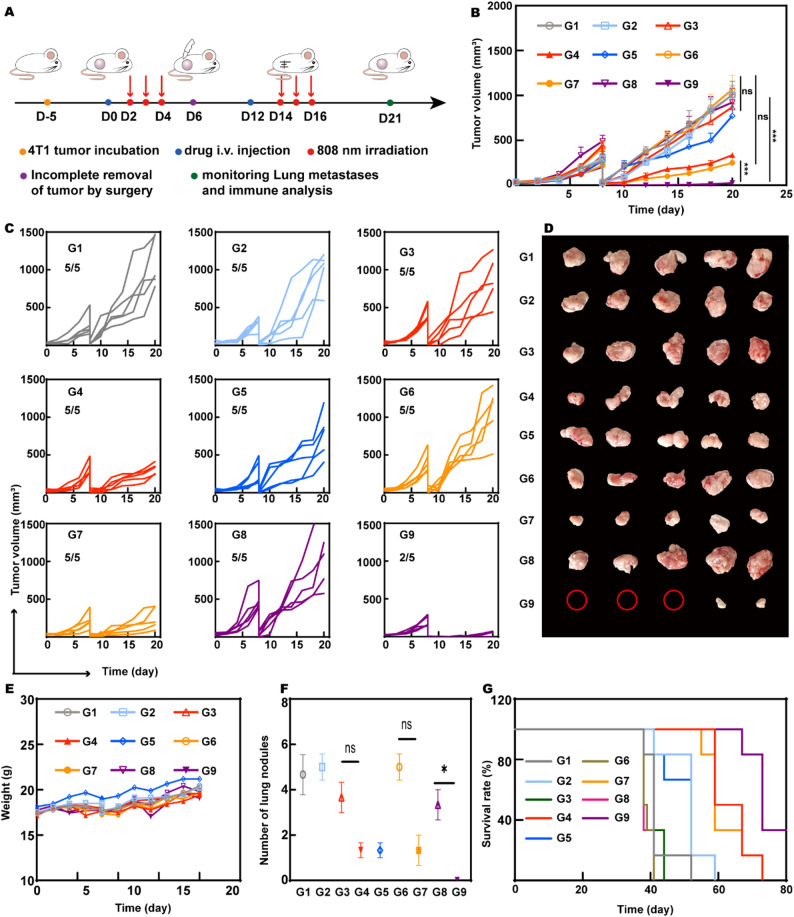
Inhibition of tumor recurrence and metastases. **A**) Experimental design of treatments in 4T1 tumor-bearing mice. **B**) Average tumor growth curves of tumor-bearing mice after treatments. *n* = 5 animals per group. **C**) Individual tumor suppression curves after different treatments. **D**) Photos of recurrent tumors on day 21 after the first administration. **E**) Body weight of mice. **F**) Numbers of nodules in the lungs of mice on day 21 after the first administration. (*n* = 3 animals per group) **G**) Survival curves of mice after different treatments (*n* = 6 animals per group). G1-G9: PBS, DMXAA, UPD, UPD(+), EcN_flaB_, EcN_flaB_@U, EcN_flaB_@U(+), EcN_flaB_@UPD, and EcN_flaB_@UPD(+). Data are presented by means ± SEM. *P < 0.05, **P < 0.01, ***P < 0.001

As exhibited in Fig. 6A, 4T1 tumor-bearing mice were administered via i.v. injection with PBS, DMXAA, UPD, and EcN_flaB_@UPD, respectively. At 48 h after administration, mice were irradiated with 1 W/cm^2^ NIR light for 15 min for three consecutive days. On day 6 after the first administration, tumors were partly surgically removed, and 5% of the tumor volume remained to simulate tumor recurrence. Then, a second round of drug administration and subsequent phototherapy began on postoperative day 6. As indicated in Fig. 6B, C and D, significant tumor suppression effects were found in the UPD(+), EcN_flaB_@U(+), and EcN_flaB_@UPD(+) groups, wherein the EcN_flaB_@UPD(+) possessed the best tumor suppression effect. The postoperative recurrence was found in only two out of five mice in the EcN_flaB_@UPD(+) group, whereas 100% postoperative recurrence was found in all other groups, which demonstrated EcN_flaB_@UPD(+) possessed excellent ability of inhibiting tumor recurrence. During the treatment period, there was no significant change in the body weight of the mice (Fig. 6E), indicating this treatment possesses good biosafety.

Lung metastasis of breast cancer is a major cause of therapy failure after surgery [53]. It has been reported that 4T1 tumor cells could spontaneously disseminate to the lungs [54]. On day 21 after the first administration, lungs from mice were collected, and the white pulmonary metastasis foci were counted and photographed (Figure S38 and Fig. 6F). Metastatic nodules were found in PBS, free DMXAA, PD, EcN_flaB_@U, and EcN_flaB_@UPD groups without NIR illumination. In contrast, no metastatic foci were found in the EcN_flaB_@UPD(+) group, suggesting that EcN_flaB_@UPD can inhibit metastasis of the 4T1 tumor when exposed to NIR light.

In addition, EcN_flaB_@UPD(+) improved the animal survival time (Fig. 6G). No mice survived in the PBS group more than 41 days after the first administration. While the maximum survival time of mice in the UPD(+) and EcN_flaB_@U(+) groups was 77 days. In the EcN_flaB_@UPD(+) group, two out of six mice survived over the 80-day observation period. According to the survival period, it was deduced that EcN_flaB_@UPD(+) possessed great potential in inhibiting postoperative tumor recurrence and metastases.

**Fig. 7:**
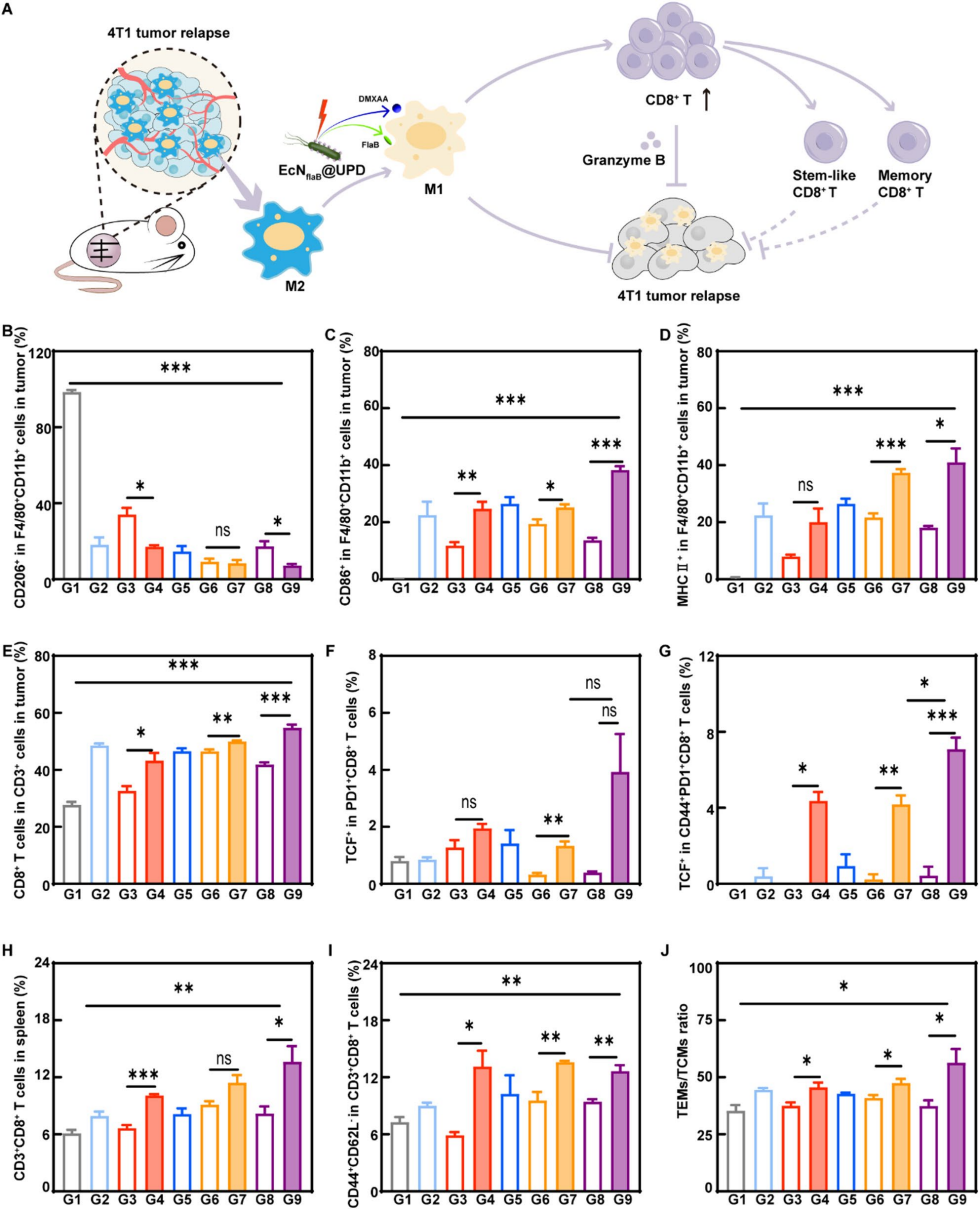
Immune responses following treatments in the model of postoperative recurrence. **A**) The diagrammatic representation illustrating the mechanisms of anti-tumour recurrence of EcN_flaB_@UPD. Quantitative analysis of the levels of A) CD206, **B**) CD86, and **C**) MHC II on macrophages in the recurrent tumor. Cells were pre-gated from F4/80^+^CD11b^+^ cells. (*n* = 4 animals per group) The proportion of **D**) CD8^+^ T lymphocytes, **E**) Stem-like TILs (TCF-1^+^PD-1^+^CD8^+^T cells) and **F**) Memory progenitor TILs (TCF-1^+^PD-1^+^CD44^+^CD8^+^T cells). (*n* = 3 animals per group) Flow cytometric analysis of **G**) CD 8^+^ T lymphocytes **H**) CD 8^+^ T effector memory T cells (CD44^+^CD62L^-^CD8^+^T cells) in spleens. **I**) The ratio of TEMs/TCMs in CD8^+^ T cells. (*n* = 3 animals per group) G1-G9: PBS, DMXAA, UPD, UPD(+), EcN_flaB_, EcN_flaB_@U, EcN_flaB_@U(+), EcN_flaB_@UPD, and EcN_flaB_@UPD(+). Data are presented by means ± SEM. *P < 0.05, **P < 0.01, ***P < 0.001

As surgery recruits a large number of M2-like TAMs to promote immune escape and survival of the residual tumor cells [55], we detected the macrophages and macrophage-related immune cells to illustrate the mechanism by which EcN_flaB_@UPD(+) inhibited tumor relapse and metastasis. As shown in Fig. 7B-D and Figure S39, a considerable quantity of M2-type while fewer M1-type macrophages infiltration at the recurrent tumor site after surgical resection in the PBS group. In contrast, a marked number of M1-like while fewer M2-polarized macrophages were found in EcN_flaB_@UPD(+)-treated tumors. M1-polarized macrophages are facile to specifically activate T cells [56]. As shown in Fig. 7E, a notable increase in CD8^+^ T cells was found in the EcN_flaB_@UPD(+) group. To achieve a long-lasting antitumor effect, it is crucial to guarantee a durable antitumor T-cell immune response. Both stem-like T cells and memory T cells have recently emerged as key determinants of cancer immunotherapy [57]. It was reported that the activation of the STING pathway [58] and maturation of antigen-presenting cells [59, 60] could maintain and activate CD8 stem-like T cells. In this study, a significant increase in stem-like TILs (TCF-1^+^PD-1^+^CD8^+^T cells) (Fig. 7F) and in memory progenitor TILs (TCF-1^+^PD-1^+^CD44^+^CD8^+^T cells) (Fig. 7G) were found in the EcN_flaB_@UPD(+) group by flow cytometric analysis (Figure S40). Furthermore, CD8^+^ T cells in the spleens notably increased in the EcN_flaB_@UPD(+) group (Fig. 7H and Figure S41). Effector memory T cells (TEMs) served as immediate antigen-primed cells that entered into peripheral tissues and mediated inflammation or cytotoxicity. In contrast, central memory T cells (TCMs) could exert cytotoxic function only after effective differentiation into TEMs [61]. The proportion of TEMs (CD44^+^CD62L^−^) in CD3^+^CD8^+^ T subtypes in EcN_flaB_@UPD(+) group was considerably higher than that in the PBS group (Fig. 7I and Figure S42). In addition, the ratio of TEMs/TCMs in the EcN_flaB_@UPD(+) group was significantly higher than that in the control group (Fig. 7J). Taken together, as depicted in Fig. 7A, surgery has been shown to increase the infiltration of M2-like tumor-associated macrophages (TAMs), which may contribute to promote tumor relapse. However, when treated with EcN_flaB_@UPD(+), the enhanced macrophage M1-type macrophages and CD8^+^ T cells exhibited durable antitumor immunity. Furthermore, there is a significant increase in the population of stem-like and memory CD8^+^ tumor-infiltrating lymphocytes (TILs), as well as effector memory T cells in the EcN_flaB_@UPD(+) group. Stem-like TILs possess the capacity for self-renewal and adaptation, while memory TILs and effector T cells facilitate rapid and effective responses when exposed to the same antigen [57]. These lymphocyte subsets can effectively prevent 4T1 tumor recurrence and metastases.

### Safety evaluation of EcN_flaB_@UPD(+) in vivo

**Fig. 8:**
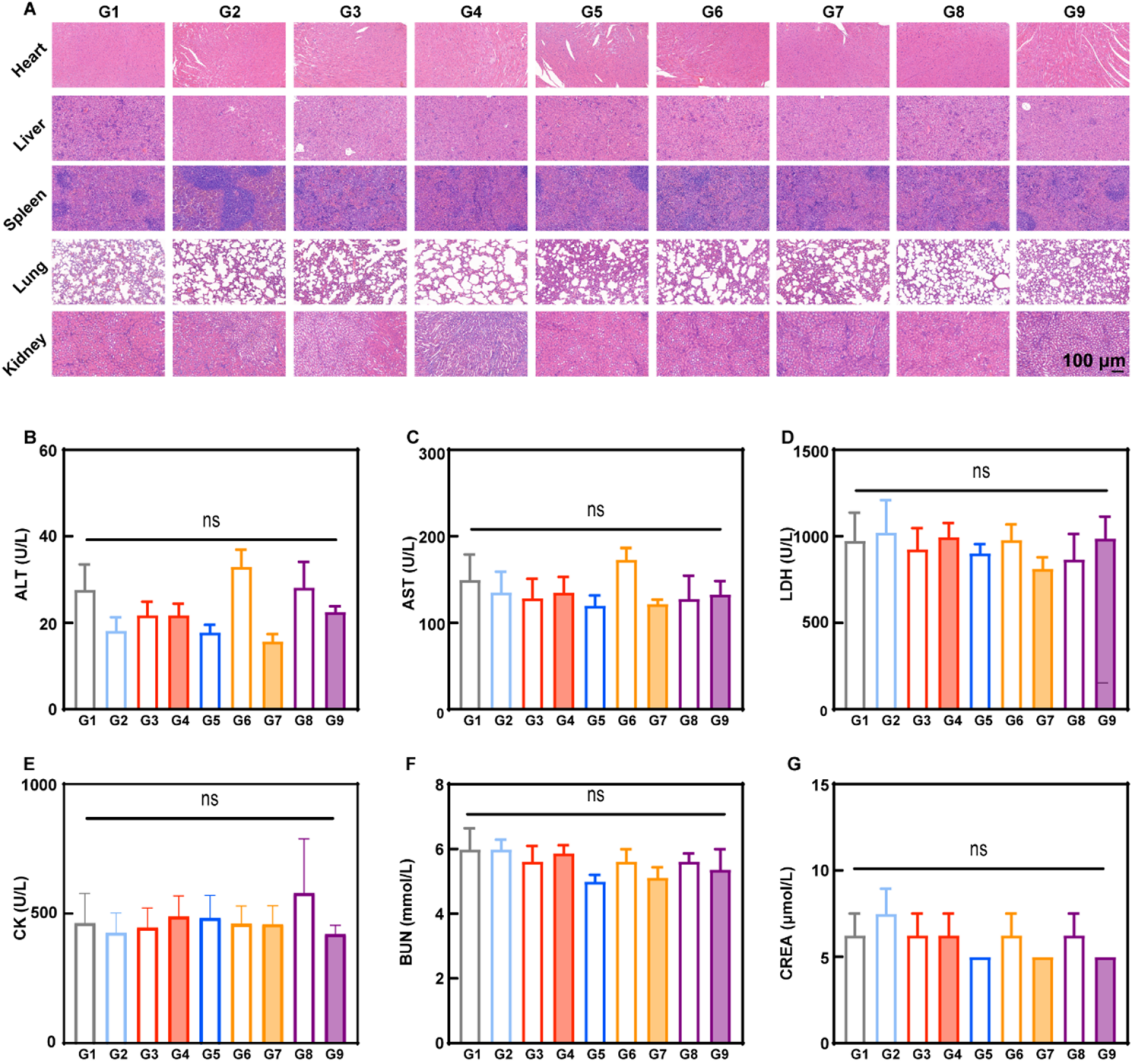
The biosafety analysis of EcN_flaB_@UPD(+) in vivo. **A**) H&E staining images of major organs of mice after different treatments. **B-G**) Serum levels of alanine aminotransferase (ALT), aspartate aminotransferase (AST), lactate dehydrogenase (LDH), creatine kinase (CK), blood urea nitrogen (BUN), and creatinine (CREA) in mice on day 17 after treatments. *n* = 4 animals per group. G1-G9: PBS, DMXAA, UPD, UPD(+), EcN_flaB_, EcN_flaB_@U, EcN_flaB_@U(+), EcN_flaB_@UPD, and EcN_flaB_@UPD(+). Data are presented by means ± SEM. *P < 0.05, **P < 0.01, ***P < 0.001

The body weight variation was monitored. No significant differences were found in the body weights of tumor-bearing mice after different treatments (Figure S43). At the endpoint of treatments, the main tissues of mice were dissected and stained with H&E sections, and no apparent necrosis or destruction of physiological structure was observed in the heart, liver, spleen, lung, and kidney tissues after different treatments (Fig. 8A). Moreover, no significant changes in the blood biochemical indexes were found in all groups (Fig. 8B-G). Therefore, it was deduced that EcN_flaB_@UPD(+) possessed good biosafety.

## Conclusion

In summary, we have successfully developed an optogenetics-engineered EcN conjugating prodrug (EcN_flaB_@UPD) for precision tumor therapy, possessing superior spatiotemporal controllability. When exposed to near-infrared (NIR) irradiation, ultraviolet and blue light emitted from upconversion nanoparticles (UCNPs) could induce flaB and DMXAA release from EcN_flaB_@UPD. The released flaB and DMXAA effectively repolarized tumor-associated macrophages (TAMs) from the immunosuppressive M2 phenotype to the antitumor M1 phenotype, primarily through the activation of the TLR5-NF-κB and STING-NF-κB signaling pathways. M1 macrophages subsequently mediate tumor cell cytotoxicity via direct phagocytosis and indirect killing, including inducing cytokine production, recruiting and activating CD8^+^ T cells and NK cells. In vivo studies revealed that EcN_flaB_@UPD(+) significantly inhibited tumor growth, and prevented tumor relapse and metastasis by inducing systemic antitumor immune memory with the activation of stem-like CD8^+^ T cells and memory CD8^+^ T cells. Our findings underscore the potential of dually NIR-triggered STING proagonists-bacteria conjugates for cancer-specific immunotherapy. Future work will focus on scaling up the platform and expanding the dual-stimulus strategy to other agents or tumor types.

## Materials and methods

### Experimental materials and chemicals

Y(CH_3_CO_2_)3 (99.9%), Yb (CH_3_CO_2_)3 (99.9%), Tm (CH_3_CO_2_)3 (99.9%), oleic acid (OA) and 1-octadecylene (ODE) were purchased from Sigma-Aldrich. Ammonium fluoride (NH_4_F). methanol, and NaOH were obtained from Sinopharm. 4-[4-(1-hydroxyethyl)-2-methoxy-5-nitrophenoxy]butanoic acid (95%) and Vadimezan (DMXAA, 98%) were purchased from Shanghai Bid PharmaTech Co. N-hydroxysuccinimide (NHS, 99%). N, N-diisopropylethylamine (DIPEA N-hydroxysuccinimide (NHS, 99%), N, N-diisopropylethylamine (DIPEA), and 1-(3-dimethylaminopropyl)-3-ethylcarbodiimide hydrochloride (EDC HCl) were purchased from Shanghai McLean Biochemistry Science and Technology Company Limited. DSPE-PEG2000-Mal (Mw = 2941.61) and DSPE-PEG2000 (Mw = 2748) were purchased from Wuhan Kostan Biotech Co. Hydroxybenzotriazole anhydrous (HOBT), furfurylamine, and 4-Dimethylaminopyridine (DMAP) were purchased from Shanghai Aladdin Biochemical Science and Technology Co. Ficoll-paque plus was purchased from GE Healthcare. FreeZol Reagent (Item No. R711), ChamQ Universal SYBR qPCR Master Mix (Item No. Q711), and One-Step Reverse Transcription Kit (Item No. R333) were purchased from Novozymes. True-Nuclear™ Transcription Factor Buffer (Set 424401), FITC anti-mouse CD3 antibody (Item 100203), PE/Cyanine7 anti-mouse CD8a antibody ((Item No. 100721), Brilliant Violet 421™ anti-mouse CD4 antibody (Item No. 100438), PE anti-mouse/human CD44 antibody (Item No. 103007), Brilliant Violet 421™ anti-mouse I-A/I-E antibody (Item No. 107632), APC/Cyanine7 anti-mouse CD45 antibody (Item No. 103116), APC anti-mouse CD279 (PD-1) antibody (Item No. 135210), PerCP/Cyanine5.5 anti-mouse CD62L antibody (Item No. 104421), FITC anti-mouse CD11b (Item No. 101205), Peridinin chlorophyllin-Cyanine5.5 (PC5.5) anti-mouse F4/80 antibody (Item No. 123117), APC anti-mouse CD206 antibody (Item No. 141723), PE anti-mouse CD86 antibody Item No. 127606), FITC anti-mouse CD11c antibody((Item No.101205), and APC anti-mouse CD80 (Item No. 104715) antibody were all purchased from Biolegend, Inc. The kits of NO and LDH were brought from Beyotime Biotechnology Co. The ELISA kits of TNF-α, IFN-β, IL-6, IL-1β and IFN-γ were purchased from Wuhan Moshak Biotechnology Co., Ltd.

### Cell, bacterium, and animal

The mouse breast cancer 4T1 cells and human umbilical vein endothelial cells (HUVECs) were cultured in RPMI 1640 containing 10% FBS and penicillin-streptomycin (100 IU/mL and 100 mg/mL). The mouse RAW264.7 macrophages were maintained in a DMEM high glucose medium containing 10% FBS and penicillin-streptomycin (100 IU/mL and 100 mg/mL). The EcN_flaB_ strain was constructed previously in our laboratory, containing the recombinant plasmid pET20b-EL222-pelB-flaB-mCherry in which the genes code for blue light-responsive EL222 sensor protein EL222, a reporter mCherry, and flaB. And the pelB signal sequence within the plasmid pET20b-EL222-pelB-flaB-mCherry can induce the secretion of flaB-mCherry infusion protein. The EcN_flaB_ was capable of producing the flaB after exposure to blue light, was cultured in LB medium at 37 °C. BALB/c mice (females, 15–17 g) were purchased from Three Gorges University, China.

### Preparation and characterization of EcN_flaB_@UPD

#### Preparation of UPD nanoparticles

UCNPs (NaYF_4_:Yb, Tm@NaYbF_4_:Nd@NaGdF_4_) were synthesized according to our previous report [30]. The synthesis method for PD is detailed in the Supporting Information. Subsequently, 5 mg of PD and 5 mg of DSPE-PEG2000-Mal were added into 10 mL of dichloromethane and stirred overnight. Then, 20 mg of UCNP and 10 mg of DSPE-PEG2000 were added, and the mixture was sonicated for 5 min. After dry-spun, 8 mL of ultrapure water was added and the mixture was sonicated for 30 min. The product was washed twice with ultrapure water and dispersed in water to obtain UPD. The UCNP without the PD was prepared as a control, to obtain the water-soluble UCNP (named U).

#### Preparation of EcN_flaB_@UPD

200 µL of UPD solution (5 mg/mL) was added to 100 µL of PBS suspension containing 1 × 10^7^ CFU EcN_flaB_, and the mixture was incubated for 2 h at 37 ℃ on a shaker with protection from light. After centrifuging at 8000 rpm for 5 min, the deposit was washed with PBS three times to obtain EcN_flaB_@UPD. Thereafter, the deposit was resuspended in 1 mL of PBS. Additionally, EcN_flaB_@U was obtained by cross-linking the water-soluble UCNP with EcN_flaB_ using the same method.

To evaluate the stability of the conjugate in a physiological environment, UPD nanoparticles were labeled with IR780 and conjugated to bacteria to prepare EcN_flaB_@UPD-IR780. Free UPD-IR780 nanoparticles were used as a control. The conjugates and controls were incubated in PBS containing 10% mouse serum at 37 °C under static conditions. At predetermined time points (0, 0.5, 1, 3, 6, and 12 h), samples were collected and centrifuged at 8,000 rpm for 5 min to separate bacteria-bound nanoparticles (pellet) from free nanoparticles (supernatant). The fluorescence intensity of IR780 in both fractions was measured using the fluorescence master system (RF-5301 PC Shimadzu, Japan) (Ex/Em = 780/820 nm). All measurements were performed in triplicate at each time point. The binding efficiency was calculated as: Binding efficiency (%) = [FLpellet / (FLpellet + FLsupernatant)] × 100%.

#### Quantitative analysis of UPD cross-linking on EcN_flaB_@UPD

EcN_flaB_@UPD was prepared by cross-linking 1 mg of UPD with 1 × 10^7^ CFU bacteria. Concentrated nitric acid was added to 1 mg of UCNP, 1 mg of UPD, and 1 × 10^7^ CFU of EcN_flaB_@UPD, respectively, boiled to dryness at 300 °C. The content of the Y-element of each material was determined by ICP-MS. The amount of cross-linked UPD on bacteria (1 × 10^7^ CFU) was calculated.

#### Particle size and potential analysis

U, UPD, EcN_flaB_, EcN_flaB_@U, and EcN_flaB_@UPD were dispersed in ultrapure water, respectively. The particle size and ζ-potential were determined by the Malvern Mastersizer 2000 particle size analyzer. The colloidal stability of UPD was evaluated by monitoring its hydrodynamic diameter and polydispersity index (PDI) over time in phosphate-buffered saline (PBS, pH 7.4). UPD nanoparticles were dispersed in PBS at a concentration of 0.1 mg/mL and incubated at 37 °C under static conditions. At predetermined time points (0, 12, 24, 48, and 72 h), aliquots of the sample were taken, and the particle size and PDI were measured using DLS on the Malvern Zetasizer. Each measurement was performed in triplicate, and the results are presented as the mean ± SEM.

#### Morphological characterization

U, UPD, EcN_flaB_, EcN_flaB_@U, and EcN_flaB_@UPD were dropped onto the carbon-supported membrane copper mesh, respectively. The morphology of each sample was recorded by a Hitachi HT7700 Bio-Transmission Electron Microscope. UPD energy spectra were characterized on a field emission transmission electron microscope (Tecnai G2 F30).

#### Determination of UV absorption and fluorescence emission spectra

The UV absorption spectra of DMXAA, U, UPD, EcN_flaB_, EcN_flaB_@U, and EcN_flaB_@UPD were determined at spectrum of 250–500 nm on a UV-Vis Spectrophotometer (UV-2600 Shimadzu, Japan). The emission spectra of the aforementioned materials were determined on the fluorescence master system (RF-5301 PC Shimadzu, Japan) under the illumination of 808 nm near-infrared light, respectively.

#### Detection of drug loading and drug release of UPD

UPD (0.5 mg) was dispersed into 500 µL PBS/DMSO (9:1) mixture and irradiated with UV light. The concentration of DMXAA was measured periodically until no changes could be found. Based on the DMXAA standard curve, the released amount of DMXAA was calculated, and then the proportion of DMXAA on the UPD was determined. The chemical composition at the molecular level was determined using a Time of Flight Mass Spectrometry (LC-MS) (UltiMate3000—microTOFII, Thermo Fisher Scientific–Brooke Dalton, USA). Furthermore, one mg/mL of UPD dispersed in PBS/DMSO was added into a 24-well plate (500 µL /well). After irradiation with an 808 nm near-infrared laser at a power density of 1 W/cm^2^ for 0, 15, 30, 45, 60, and 90 min, respectively, samples were centrifuged to collect the supernatants. The released DMXAA in the supernatant was determined by fluorescence spectrometry. The amount of the released DMXAA was calculated and the release curve was plotted using standard curve calibration. The excitation wavelength of DMXAA was 345 nm, and the fluorescence emission peak was 421 nm.

#### Detection of the bacterial growth curve of EcN_flaB_@UPD

In order to investigate the impact of cross-linking UPD on the activity of bacteria, freshly prepared EcN_flaB_, EcN_flaB_@U, and EcN_flaB_@UPD were cultured in the LB medium at a ratio of 1:100. The OD_600_ values of EcN_flaB_@UPD were measured using a UV spectrophotometer to plot the bacterial growth curve at the 0, 1.5, 3, 4, 6, 8, 10, 20 and 30 h, respectively. In addition, to investigate the effect of NIR light on the bacterial activity, EcN_flaB_ (without light), EcN_flaB_@U(+), and EcN_flaB_@UPD(+) ((+) indicates illumination under 1 W/cm^2^ 808 nm NIR light for 1.5 h) were cultured in a shaking bed at 37 ℃. The OD_600_ values were measured at different time points, and the growth curves were plotted.

#### Near-infrared light-induced drug release of EcN_flaB_@UPD

For in vitro and in vivo experiments, irradiation was performed using an 808 nm NIR laser. The laser spot size was adjusted to fully cover the target area (well or tumor). The irradiation distance was fixed at 1 cm.

The EcN_flaB_, EcN_flaB_@U, and EcN_flaB_@UPD were irradiated by NIR light of 808 nm for 10, 30, and 60 min at the power density of 1 W/cm^2^, respectively. Then, mCherry fluorescence intensity was used to quantify the level of mCherry/flaB fusion protein expression. In addition, the fluorescence values of DMXAA and mCherry were measured at 30, 60, 90, and 120 min, respectively, when the EcN_flaB_@UPD was irradiated with 1 W/cm^2^ 808 nm near-infrared light.

### In vitro test

#### Morphological analysis of the polarized macrophages induced by EcN_flaB_@UPD(+) in vitro

Pretreatment of materials: DMXAA (5.26 µg/mL), UPD (The content of DMXAA is 5.26 µg/mL), EcN_flaB_ (2 × 10^6^ CFU/mL), EcN_flaB_@U (2 × 10^6^ CFU/mL) and EcN_flaB_@UPD (2 × 10^6^ CFU/mL), were dissolved separately in a fresh medium. In which, the EcN_flaB_@U(+) and EcN_flaB_@UPD(+) groups were irradiated with 808 nm near-infrared laser with a power of 1 W/cm^2^ for 1.5 h. After centrifugation at 8,000 rpm, the supernatants were filtered through 0.22-µm sterilized filter membranes.

Morphological change of macrophage polarization from M0 to M1: RAW264.7 cells (M0) were inoculated into a 12-well plate at a density of 5 × 10^5^ cells per well and incubated for 12 h. Thereafter, one mL of the pretreated materials was added (grouped as follows: PBS, DMXAA, UPD, UPD(+), EcN_flaB_, EcN_flaB_@U, EcN_flaB_@U(+), EcN_flaB_@UPD, EcN_flaB_@UPD(+)). Following incubation for 96 h, the macrophage morphology was observed and photographed using an inverted fluorescence microscope.

Morphological change of repolarized macrophage from M2 to M1: Firstly, RAW264.7 cells(M0) (5 × 10^5^ cells/well) were spread in a 12-well plate and incubated for 12 h. Secondly, one mL of DMEM fresh medium containing 10 ng IL-4 in each well was used to replace the primary medium, and then incubated for 12 h (for obtaining M2). Thereafter, the medium was replaced by one mL of fresh DMEM complete medium and 500 µL of pretreated materials for 72 h incubation. Finally, the cells were collected, and the activation of macrophages was observed and photographed using the inverted fluorescence phase contrast microscope (Ti-2 Nikon Japan).

#### Flow cytometric analysis of polarized macrophages

The polarization of M0 type macrophages: RAW264.7 cells were treated as described in section Morphological analysis of the polarized macrophages induced by EcN_flaB_@UPD(+) in vitro. After a 48-hour incubation, the cells and supernatants were collected, respectively. After washing twice with PBS, the cells were stained with APC-CD206 and PE-CD86 antibodies, and analyzed by flow cytometry (CytoFLEX, Beckman, China). Furthermore, the levels of NO, TNF-α, and IFN-β in the supernatants were measured by the corresponding kits. The repolarization of M2 type macrophages: Similar to the macrophage repolarization procedure in Morphological analysis of the polarized macrophages induced by EcN_flaB_@UPD(+) in vitro, after incubation with the different materials for 48 h, the cells were stained with APC-CD206 and PE-CD86 antibodies and analyzed by flow cytometry.

#### 4T1 cell viability analysis after treatment with the supernatants of EcN_flaB_@UPD(+)-induced macrophages

4T1 cells were inoculated in a 96-well plate at a density of 5000 cells per well and incubated overnight. After discarding the medium, 200 µL of polarized macrophage supernatant was added. Following 24-hour incubation, the viability of 4T1 cells was detected by CCK-8 kits.

#### The phagocytosis analysis of EcN_flaB_@UPD(+)-induced macrophage

Macrophage polarization from M0 to M1 was performed as described in Morphological analysis of the polarized macrophages induced by EcN_flaB_@UPD(+) in vitro. Then, macrophages were collected and stained with FITC-CD11b antibody. 4T1 cells were stained with Dio. The stained macrophages and 4T1 cells were co-incubated in a ratio of 1:2 for 12 h. After washing with PBS, the phagocytosis of 4T1 cells by macrophages was detected by flow cytometry.

#### Activation of CD8^+^ T cells and NK Cells in spleen

Extraction of splenic lymphocytes: Spleens were removed from 6-week-old mice, rinsed with PBS repeatedly, and then punctured the spleens to release splenic cells into PBS. The cell suspension was filtered with a cell sieve and collected by centrifugation at 800 g for 5 min. After resuspension with 3 mL of PBS, the cell suspension was slowly added to a 15 mL centrifuge tube containing 3 mL of Ficoll isolate. A flat rotor was used to centrifuge at 800 g for 20 min, and the middle cloudy liquid was carefully aspirated. After washing with PBS twice, the obtained splenic lymphocytes were added into the 1640 medium for culture.

Activation of CD8^+^ T cells and NK cells in splenic lymphocytes: The 4T1 cells were collected and freeze-thawed five times to lyse the cells to expose the antigen. Then, macrophages were plated in a 12-well plate (5 × 10^5^ cells/well). One mL of pretreated material supernatant (treated as described in section Morphological analysis of the polarized macrophages induced by EcN_flaB_@UPD(+) in vitro) was added into each well for 24 h incubation. Thereafter, 50 µL of 4T1 cell lysate (with a protein concentration of 1 µg/µL) was added to each well. After a further 24 h incubation, the macrophages were collected. The extracted splenic lymphocytes and macrophages were incubated in a 1:1 ratio for three days. Subsequently, the cells were collected, stained by FITC-CD3, PE-CD8, APC-CD49b, and PC7-Granzyme B anti-mouse antibodies, and analyzed by flow cytometry.

#### Cytotoxic lymphocytes-mediated 4T1 killing

Splenic lymphocytes after the co-stimulation of antigen and macrophages mentioned above were collected, and co-cultured with 4T1 cells at the E/T ratios (Effector/target cell ratio) of 5:1, 10:1, and 40:1 in a cell culture incubator for six hours. The supernatants were collected and the specific killing effect of macrophage-stimulated splenic lymphocytes on 4T1 cells was evaluated by measuring the LDH level using an LDH Kit.

#### Activation of BMDCs by EcN_flaB_@UPD(+)

BMDCs were extracted and cultured by adding IL-4 and GM-CSF according to the report [62]. Subsequently, BMDCs were inoculated in 6-well plates at a density of 2 × 10^6^ cells/well. Two mL 1640 medium (containing the treated materials previously described) was added into the well and incubated for 48 h. The cells were stained with FITC-CD11c, PE-CD80, and APC-CD86 anti-mouse antibodies and analyzed by flow cytometry to determine BMDC maturation.

#### Evaluation of EcN_flaB_@UPD(+) on vascular formation in vitro

Tube forming assay: HUVECs were cultured in DMEM containing 0.2% FBS and penicillin-streptomycin (100 IU/mL and 100 mg/mL) for 24 h. After being melted at 4 ℃ overnight, 135 µL of the matrix gel was spread uniformly in the well of a 48-well plate on ice (avoiding premature solidification). Subsequently, the plates were incubated in an incubator at 37 ℃ for 1 h to solidify. HUVECs were added at 1 × 10^5^ cells/well, followed by adding 20 µL pretreated medium with different materials. After incubation for 6 h, the vascular network was photographed using an inverted fluorescence microscope and quantified using the Angiogenesis Analyzer plug-in for Image J.

Scratch assay: To investigate the impact of EcN_flaB_@UPD on HUVEC migration, an in vitro scratch assay was conducted. HUVECs were inoculated into a 12-well plate at 5 × 10^5^ cells/well. When the cells fused into a monolayer state, a scratch from top to bottom was scraped using a sterile 200 µL pipette tip and washed with PBS to remove cell debris. Thereafter, one mL of pre-treated solution with different materials with or without light irradiation was added. At 24 h after the scraping, the cells were washed twice with PBS and fixed with paraformaldehyde for 15 min. After washing two times with ultrapure water, crystal violet staining solution was added at 500 µL/well. Finally, the mixture was washed three times with ultrapure water before being photographed using an inverted optical microscope. The migration rate was analyzed using Image J software and calculated as follows: migration area (%) = (A_0_-A_T_)/A_0_ × 100%, where A_0_ indicates the area of the initial wound and A_T_ indicates the remaining area of the wound at the corresponding time point.

#### Quantitative real-time PCR analysis

RAW264.7 cells were spread in a 6-well plate at a density of 1 × 10^6^ cells per well, and two mL of DMEM complete medium was added into each well. After incubation for 10 h, one mL of medium was discarded from each well. Then, one mL of pretreated material supernatant (treated as section Morphological analysis of the polarized macrophages induced by EcN_flaB_@UPD(+) in vitro described ) was added and incubated for 48 h. Total RNA was extracted using TRIzol reagent and reverse transcript to cDNA using a One-Step Reverse Transcription Kit. cDNAs were prepared for the real-time PCR using ChamQ Universal SYBR qPCR Master Mix. The Primer sequences were provided in Table S1 (Supplementary Information). The mRNA expression was quantified using the QS3 real-time fluorescence quantitative PCR instrument (ABI USA).

#### Western blotting assay

RAW264.7 cells were incubated with various pretreated material supernatant (treated as section Morphological analysis of the polarized macrophages induced by EcN_flaB_@UPD(+) in vitro described) for 10 h. Then, the proteins derived from RAW264.7 were extracted and further quantified by the BCA kit. Subsequently, the samples were separated through SDS-PAGE gel. The PVDF membranes that had been transferred with the separated proteins were then blocked using 5% skimmed milk. After incubation with primary antibodies, including phospho-STING (Ser365) (D8F4W) (72971 S, Cell Signaling Technology, 1:1000 dilution), STING (D2P2F) (13647, Cell Signaling Technology, 1:1000 dilution) overnight at 4 °C, the PVDF films were incubated with anti-rabbit IgG, HRP-linked antibody (A0208, Beyotime,1:1000 dilution) for another 1 h. Chemiluminescence detection was carried out for protein band visualization with ECL Substrate (Thermo Scientific, cat.32109). Following being washed with a stripping buffer, the membranes were reblocked and incubated with β-actin (AC026, abclonal, 1:100000 dilution). Then, the western blot procedure was repeated as described above.

#### Influence of EcN_flaB_@UPD(+)-mediated macrophage repolarization on the migration of 4T1 cells

The in vitro scratch experiments were conducted to investigate the effect of EcN_flaB_@UPD(+)-treated macrophages on the migration of 4T1 cells. The cells were seeded on a 12-well plate at 5 × 10^5^ cells/well. Once the cells had fused into a monolayer state, the cell monolayer was scraped from top to bottom using a sterile 200 µL pipette tip and then washed with PBS to remove cellular debris. Subsequently, one mL of the supernatant from the treated macrophages and one mL of fresh DMEM complete medium were added. At 0 and 48 h post-scratch, the cells were stained with crystal violet. Photography was conducted using an inverted optical microscope. The migration area was analyzed using Image J software.

### Animal test

### Tissue distribution analysis of EcN_flaB_@UPD

One mL of UPD at a concentration of 5 mg/mL was co-incubated with 500 µL of bacterial suspension containing 5 × 10^7^ EcN_flaB_ for 2 h at 37 °C. Subsequently, the mixture was centrifuged at 8000 rpm for 5 min and washed three times to remove the uncross-linked nanoparticles. The resulting precipitate was resuspended in 200 µL of PBS, and 5 µL of IR780 (1 mg/mL) were added. The mixture was incubated at 37 ℃ for 30 min on a shaker in the dark. Thereafter, the mixture was centrifuged and washed with PBS until the supernatant became clear. The deposit was resuspended in 1 mL of PBS.

5 × 10^6^ CFU of IR780-stained EcN_flaB_@UPD was injected into the tail vein of 4T1 tumor-bearing mouse. Fluorescence imaging was performed at 0, 6, 12, 24, 48, and 72 h after the injection using In vivo Imaging Systems (IVIS) (AniView 600, Guangzhou Biolight Biotechnology Co., Ltd). At the end of imaging, mice were euthanized, and major organs and tumors were collected for imaging (IR780, ex = 780, em = 820).

### Evaluation of tumor suppression effect of EcN_flaB_@UPD(+)

4T1 cells (2 × 10^6^ cells per mouse)were subcutaneously injected into the right flank of BALB/c mice. When the tumor volume reached about 100 mm^3^, the mice were randomly divided into nine groups and intravenously injected with PBS, DMXAA (2.63 µg per mouse), UPD (containing 2.63 µg of DMXAA per mouse), EcN_flaB_ (1 × 10^6^ CFU per mouse), EcN_flaB_@U (1 × 10^6^ CFU per mouse) and EcN_flaB_@UPD (1 × 10^6^ CFU per mouse). In the irradiation groups, the 808 nm NIR was applied to illuminate the tumor for 15 min at the power density of 1 W/cm^2^ per mouse on day 2, 3, and 4. On day 6 and day 12, the drug was intravenously injected again, and the illumination was conducted. The body weight and tumor volume of each mouse were recorded daily. The tumor volume was calculated according to the equation: *V=*$\frac{1}{2}$ *Length × Width × Width*. At the end of the experiments, the tumors and the major organs (heart, liver, spleen, lungs, and kidneys) were collected for histological analysis.

### Analysis of blood biochemical and cytokines

At the end of the experiments, the blood from mice was collected for measuring alanine aminotransferase (ALT), aspartate aminotransferase (AST), lactate dehydrogenase (LDH), creatine kinase (CK), blood urea nitrogen (BUN), creatinine (CREA) by an automatic biochemical analyzer. The levels of TNF-α, IFN-β, IL-6, IFN-γ and IL-1β in sera were detected using the ELISA kits from Wuhan Moshak Biotechnology Co., Ltd.

### H&E, TUNEL, and immunofluorescence staining

The tumors and major organs (heart, liver, spleen, lung, and kidney) were collected for H&E staining. Part of the tumor sections was used to detect the apoptosis by TUNEL. The other tumor sections were incubated with CD31 antibody and granzyme B antibody for analysis of vascular and granzyme B^+^ lymphocytes, respectively.

### In vivo immune activation

The mice were treated as described in section Evaluation of tumor suppression effect of EcN_flaB_@UPD(+). On day 16, the lymph nodes of mice were collected and homogenized into single-cell suspensions. FITC anti-mouse CD11b antibody, APC anti-mouse CD80 antibody, and PE anti-mouse CD86 antibody were used for analyzing the mature of DCs. On day 17 after treatments, tumors and spleens were harvested. The tumors were digested with collagenase (1 mg/mL, Biosharp) in a DMEM medium containing 5% FBS for 60 min at 37 °C. Subsequently, erythrocytes were removed by using an erythrocyte lysis buffer. Then the cells were filtered through a nylon mesh filter to obtain single-cell suspensions. The cell suspensions were stained with a series of fluorescent antibodies (the PE anti-mouse CD45 antibody, FITC anti-mouse CD3 antibody, PE/Cyanine7 anti-mouse CD8a antibody, Brilliant Violet 421™ anti-mouse CD4 antibody, and APC anti-mouse CD49b antibody. CD8^+^ T cells (CD45^+^ CD3^+^ CD8^+^ cells), CD4^+^ T cells (CD45^+^ CD3^+^ CD4^+^ cells), and the NK cells (CD45^+^ CD3^−^CD49b^+^ cells), were analyzed by flow cytometry. To analyze TAM populations, the following antibodies were utilized: FITC anti-mouse CD11b antibody, Peridinin chlorophyllin-Cyanine5.5 (PC5.5) anti-mouse F4/80 antibody, APC anti-mouse CD206 antibody, PE anti-mouse CD86 antibody. CD45^+^CD11b^+^F4/80^+^CD86^+^ cells were considered as the M1-like TAMs. CD45^+^CD11b^+^F4/80^+^CD206^+^ cells were considered as M2-like TAMs.

### Detection of anti-recurrence and anti-metastasis

2 × 10^6^ 4T1 cells were injected into the flank of the mice. On the fifth day after inoculation of cells, 4T1 tumor-bearing mice were randomly divided into nine groups for the administration of **G1**: PBS, **G2**: DMXAA (2.63 µg per mouse), **G3**: UPD (containing 2.63 µg of DMXAA per mouse), **G4**: UPD(+) (containing 2.63 µg of DMXAA per mouse), **G5**: EcN_flaB_ (1 × 10^6^ CFU per mouse), **G6**: EcN_flaB_@U (1 × 10^6^ CFU per mouse), **G7**: EcN_flaB_@U(+) (1 × 10^6^ CFU per mouse), **G8**: EcN_flaB_@UPD (1 × 10^6^ CFU per mouse), and **G9**: EcN_flaB_@UPD(+) (1 × 10^6^ CFU per mouse). On day 2 after the first administration, the mice in G4, G7, and G9 underwent illumination for 15 min with NIR 808 nm at 1 W/cm^2^ for three consecutive days. On day 6 after the first administration, mice were anesthetized, and 95% of the tumors were excised and sutured following experimental manipulations in the literature [63]. On day 12 after the first administration, the second administration of the drugs was performed, and illuminated on days 14, 15, and 16. Tumor volume and body weight were measured every other day during treatments. On day 21 after the first administration, the tumor, spleen, and lung tissues were harvested for analyzing the activation of immune cells.

### Evaluation of lung metastasis

The lungs from mice with different treatments were collected and fixed in Bouin’s solution (Solarbio, Beijing, China) over 12 h. After washing with 75% ethanol, the lungs were photographed, and the metastatic tumor nodes were counted.

### Analysis of immune activation in the post-surgical model

In order to investigate the activation of immune cells in the recurrent tumors induced by EcN_flaB_@UPD(+), an incomplete tumor resection model was constructed and treated as described above (*Detection of In vivo Anti-Recurrence and Anti-Metastasis*). On the 21st day after the first administration, the tumors and spleens were collected from the mice for flow cytometric analysis. M1-type macrophages (CD45^+^F4/80^+^CD11b^+^CD86^+^) and M2-type macrophages (CD45^+^F4/80^+^CD11b^+^CD86^+^), CD8^+^ T cells (CD45^+^CD3^+^CD8^+^), stem-like CD8^+^ T cells (CD45^+^CD3^+^CD8^+^PD1^+^TCF^+^), and memory-like progenitor TILs (TCF-1^+^PD-1^+^CD44^+^CD8^+^T cells) in tumor sites, as well as TEMs cells (CD45^+^CD3^+^CD8^+^CD44^+^CD62L^−^) and TCMs (CD45^+^CD3^+^CD8^+^CD44^+^CD62L^+^), were analyzed by flow cytometry.

### Survival analysis

The incomplete resection model was constructed and treated as described above (Detection of in vivo anti-recurrence and anti-metastasis). The survival time of mice was recorded. The resulting survival curves were plotted.

### Data analysis

Data are means ± SEM. Comparisons between two groups were analyzed by two-tailed unpaired Student’s *t*-test, unless otherwise indicated. For multiple-group comparisons, one-way ANOVA with Tukey’s multiple-comparisons test was performed, unless otherwise noted. Data with repeated measurements over time (e.g., tumor growth curves) were analyzed using two-way ANOVA with Tukey’s post-test after confirming that the data met the assumptions of normality and homogeneity of variance. *P* < 0.05 (*), *P* < 0.01 (**), and *P* < 0.001 (***) were defined as statistically different.

## Supplementary Information

Below is the link to the electronic supplementary material.

Supplementary Material 1

## Data Availability

The data that support the findings of this study are available from the corresponding author upon reasonable request.
