# Supplementary material for: Photoactivated probiotic micro-reactor synchronizes STING/TLRs agonists to spatiotemporally synergize antitumor immunotherapy
Source: J Nanobiotechnology. 2026 Apr 2;24:435. doi: 10.1186/s12951-026-04300-w (PMC13169788; doi:10.1186/s12951-026-04300-w)
Supplement: Supplementary file 1 — Supplementary Material 1 [file 12951_2026_4300_MOESM1_ESM.docx]

Supporting Information

**Photoactivated probiotic micro-reactor synchronizes STING/TLRs agonists to spatiotemporally synergize antitumor immunotherapy**

Yuzhi Qiu^1,2^, Yunting Liu^1,3^, Sihan Chen^1,3^, Yidi Liu^1,3^, Xi Yu^1,3^, Xiangliang Yang^1,2,3*^, Yan Zhang^1,2,3*^, Yanhong Zhu^1,2,3*^

1. National Engineering Research Center for Nanomedicine, Huazhong University of Science and Technology, 1037 Luoyu Road, Wuhan, 430074, P. R. China

2. Hubei Key Laboratory of Bioinorganic Chemistry and Materia Medica, Huazhong University of Science and Technology, 1037 Luoyu Road, Wuhan, 430074, P. R. China

3. College of Life Science and Technology, Huazhong University of Science and Technology, 1037 Luoyu Road, Wuhan, 430074, P. R. China

* Corresponding authors. College of Life Science and Technology Huazhong University of Science and Technology, 1037, Luoyu Road, Wuhan, 430074, PR China.

*E-mail addresses:* nanomedicine@mail.hust.edu.cn (X. Yang), yan_zhang@hust.edu.cn (Y. Zhang), yhzhu@hust.edu.cn (Y. Zhu).

**Synthesis of PD**

The roadmap for the synthesis of photosensitizing DMXAA precursor is shown in **Fig. S1(a)** in the following steps: 1) **Synthesis of N-(furan-2-ylmethyl)-4-(4-(1-hydroxyethyl)-2-methoxy-5-nitrophenoxy)butyramide (photolabile linker)** HOBT (203 mg, 1.5 mmol), EDC (295.65 mg, 1.5 mmol) and 4-[4-(1-Hydroxyethyl)-2-methoxy-5-nitrophenoxy]butanoic acid (1 mmol, 300 mg) were dissolved in 5 mL of dry dichloromethane (DCM) under argon atmosphere and stirred for 2 h. Then furfural amine (133 μL, 1.5 mmol) and DIPEA (262 μL, 1.5 mmol) were added and stirred overnight at room temperature. Until the reaction was completed as monitored by thin-layer chromatography (TLC) analysis, 10 mL of ethyl acetate was added, and the mixture was washed with saturated Na_2_CO_3_, saturated NaCl, and H_2_O three times, respectively. After being dried over by anhydrous MgSO_4_, the solvent was removed under vacuum to obtain the photolabile linker (Yield: 359.1mg, 95%). ^1^H NMR spectra were recorded on a Bruker 600 MHz NMR. The chemical composition at the molecular level was determined using a Fourier-transform ion cyclotron resonance mass spectrometer (FT-MS) (SolariX 7.0, Thermo Fisher Scientific-Brooke Dalton, USA). FT-MS m/z: calcd for C_18_H_23_N_2_O_7_^+^ [M + H]^+^, 379.1500; found, 379.1493. ^1^H NMR (600 MHz, DMSO-d_6_, δ): 8.37 (t, J = 5.7 Hz, 1H), 8.12 (dd, J = 8.0, 1.7 Hz, 1H), 7.87 (d, J = 8.1 Hz, 1H), 7.84 (dd, J = 7.3, 1.7 Hz, 1H), 7.55 (d, J = 1.8 Hz, 1H), 7.45 (t, J = 7.6 Hz, 1H), 7.27 (s, 1H), 7.25 (d, J = 8.1 Hz, 1H), 6.56 (s, 1H), 6.40 – 6.35 (m, 2H), 6.23 (d, J = 3.2 Hz, 1H), 4.28 (d, J = 5.4 Hz, 2H), 4.17 (d, J = 16.9 Hz, 1H), 4.06 (d, J = 17.1 Hz, 1H), 3.88 (ddt, J = 41.9, 9.3, 6.5 Hz, 2H), 3.51 (s, 3H), 2.38 (s, 3H), 2.28 (td, J = 7.3, 3.2 Hz, 2H), 2.07 (s, 3H), 1.93 (pd, J = 7.0, 2.4 Hz, 2H), 1.51 (d, J = 6.5 Hz, 3H). 2) **Synthesis of** **1-(4-(4-(4-((furan-2-ylmethyl)amino)-4-oxobutoxy)-5-methoxy-2-nitrophenyl)ethyl 2-(1,5-dimethyl-9-oxo-9H-oxa-anthracen-4-yl)acetate (PD)**: 120 mg of DMXAA, 100 mg of EDC (0.525 mmol), and 4.8 mg of DMAP (0.035 mmol) were mixed in the solution of 5 mL dichloromethane in argon atmosphere. After being stirred in the dark at room temperature for 20 min, 80 mg of the photolabile linker dissolved in 3 mL of dichloromethane was added. The mixture was heated at 40 ℃, refluxing overnight, followed by adding 50 mL of ethyl acetate. Then, the mixture was washed with saturated Na_2_CO_3_, saturated NaCl, and H_2_O three times, respectively. The combined organic phase was dried over anhydrous MgSO_4_, and the solvent was removed under vacuum. Finally, the crude residue was purified by silica gel column chromatography (ethyl acetate: petroleum ether=5:1) to obtain PD (Yield: 85.5 mg, 31.3%). FT-MS m/z: calcd for C_35_H_35_N_2_O_10_^+^ [M + H]^+^, 643.2286; found, 643.2283. ^1^H NMR (600 MHz, DMSO-*d*_6_, δ): 8.37 (t, *J* = 5.7 Hz, 1H), 8.12 (dd, *J* = 8.0, 1.7 Hz, 1H), 7.87 (d, *J* = 8.1 Hz, 1H), 7.84 (dd, *J* = 7.3, 1.7 Hz, 1H), 7.55 (d, *J* = 1.8 Hz, 1H), 7.45 (t, *J* = 7.6 Hz, 1H), 7.27 (s, 1H), 7.25 (d, *J* = 8.1 Hz, 1H), 6.56 (s, 1H), 6.40 – 6.35 (m, 2H), 6.23 (d, *J* = 3.2 Hz, 1H), 4.28 (d, *J* = 5.4 Hz, 2H), 4.17 (d, *J* = 16.9 Hz, 1H), 4.06 (d, *J* = 17.1 Hz, 1H), 3.88 (ddt, *J* = 41.9, 9.3, 6.5 Hz, 2H), 3.51 (s, 3H), 2.38 (s, 3H), 2.28 (td, *J* = 7.3, 3.2 Hz, 2H), 2.07 (s, 3H), 1.93 (pd, *J* = 7.0, 2.4 Hz, 2H), 1.51 (d, *J* = 6.5 Hz, 3H).


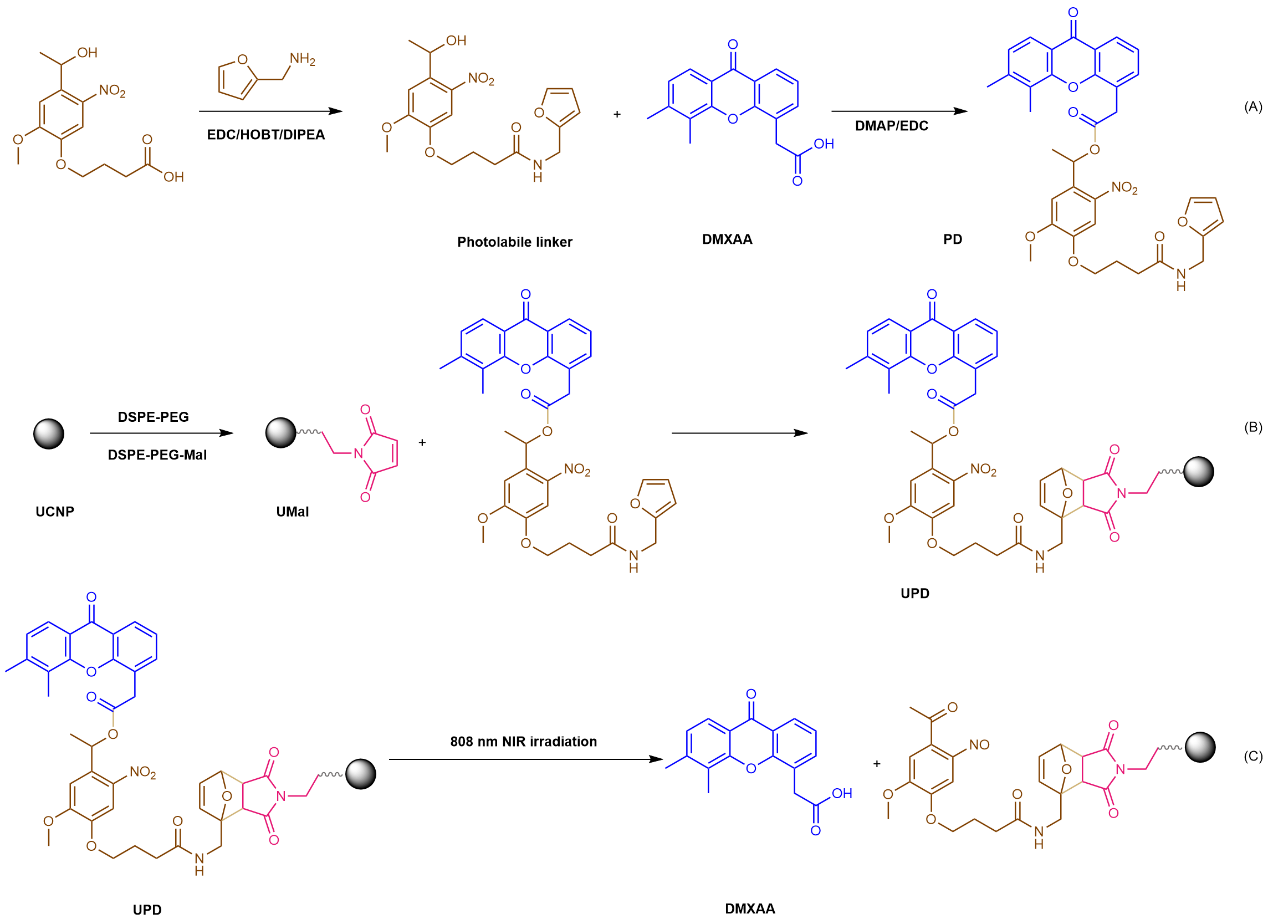


**Fig. S1.** The synthesis route of UPD. (A) Synthesis of the photolabile prodrug PD. (B) Synthesis of UPD. (C) The release of UPD under 808 nm irradiation.


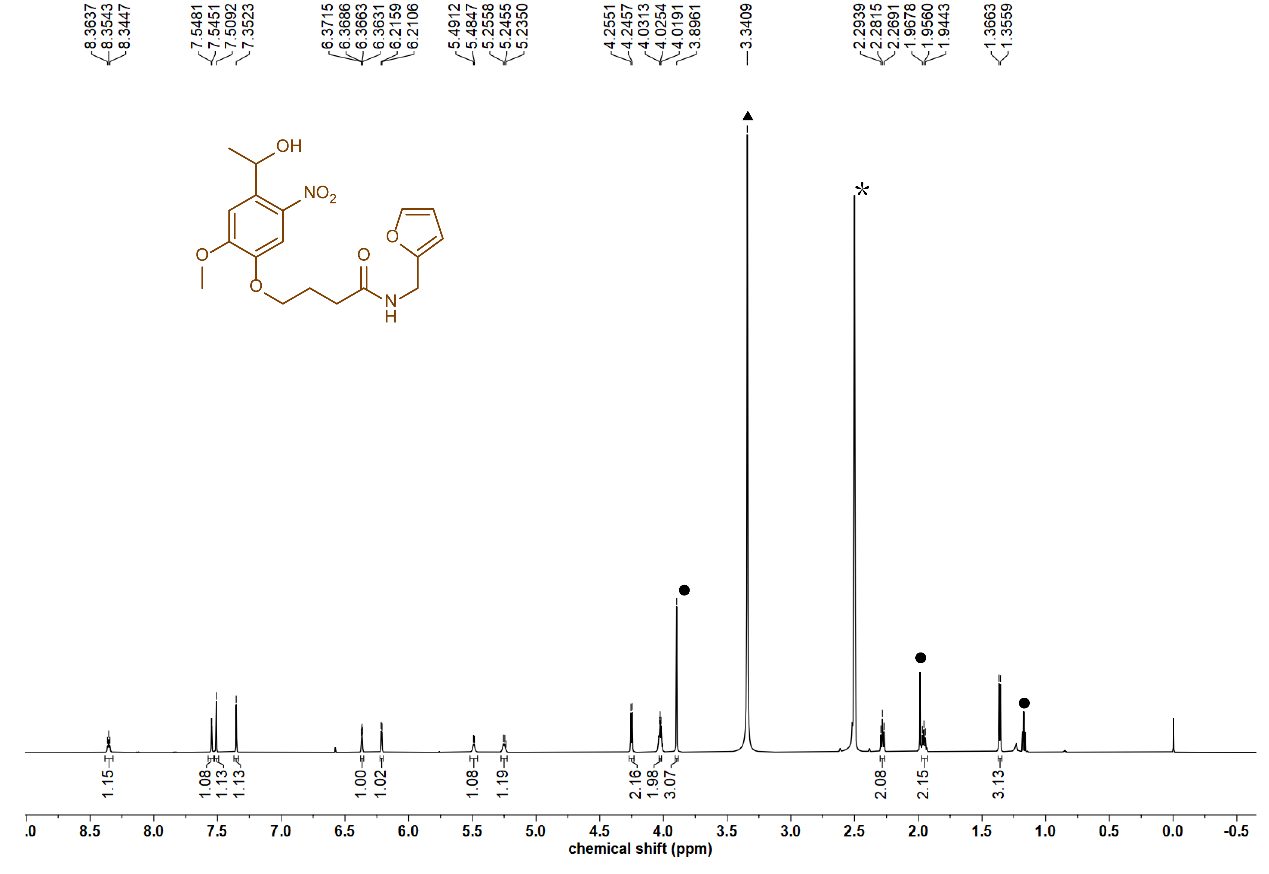


**Fig. S2.** ^1^H NMR spectrum of the photolabile linker (* DMSO ▲H2O ●Ethyl acetate).


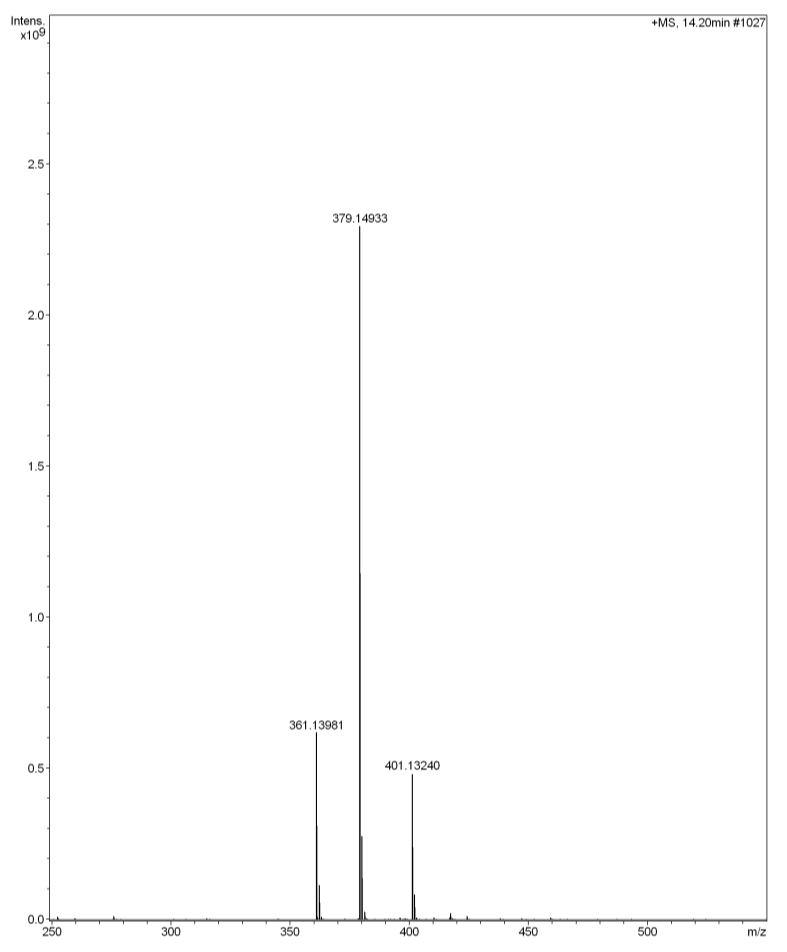


**Fig. S3.** Mass spectrometry of photolabile linker.


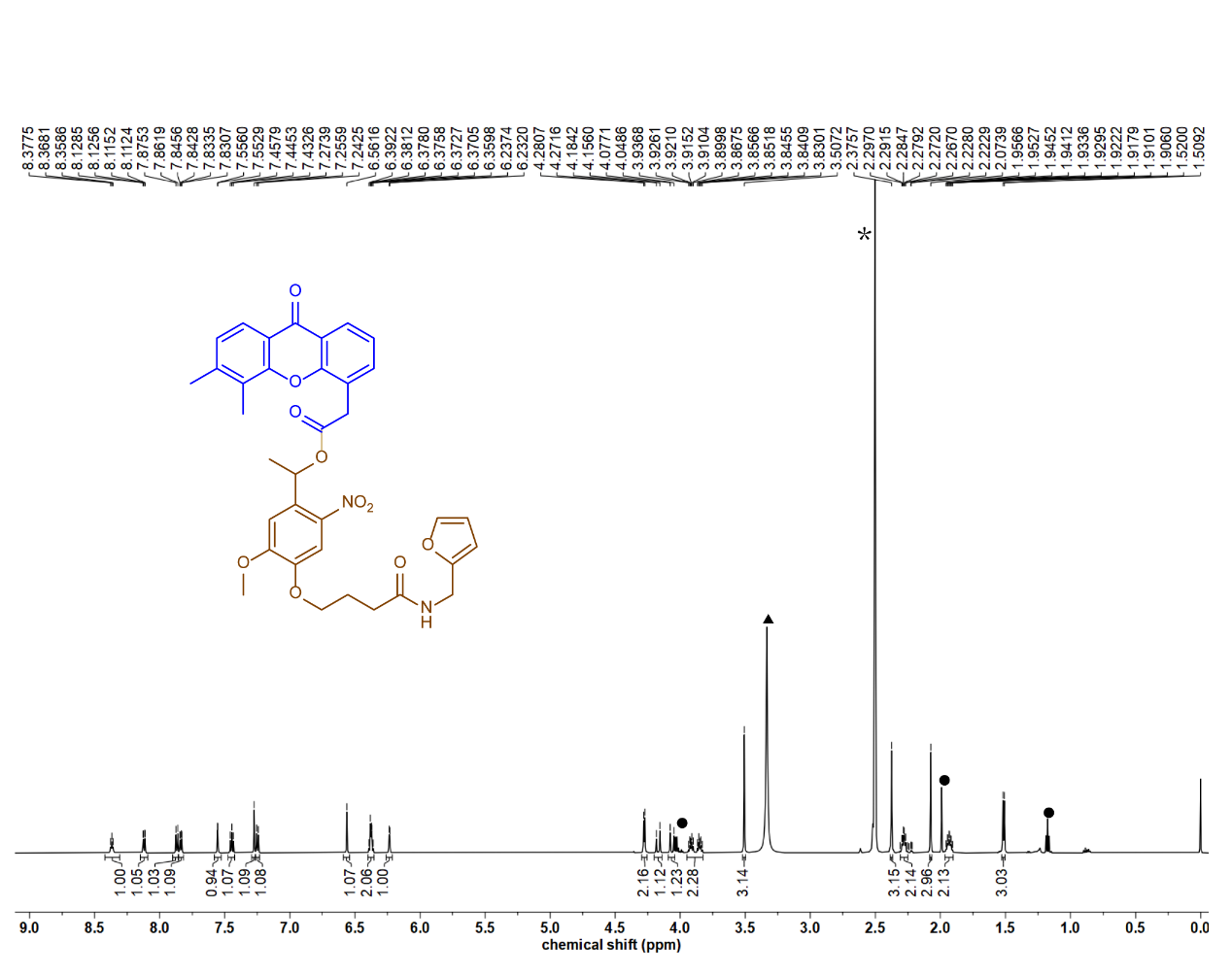


**Fig. S4.** ^1^H NMR spectrum of PD (* DMSO ▲H2O ●Ethyl acetate).


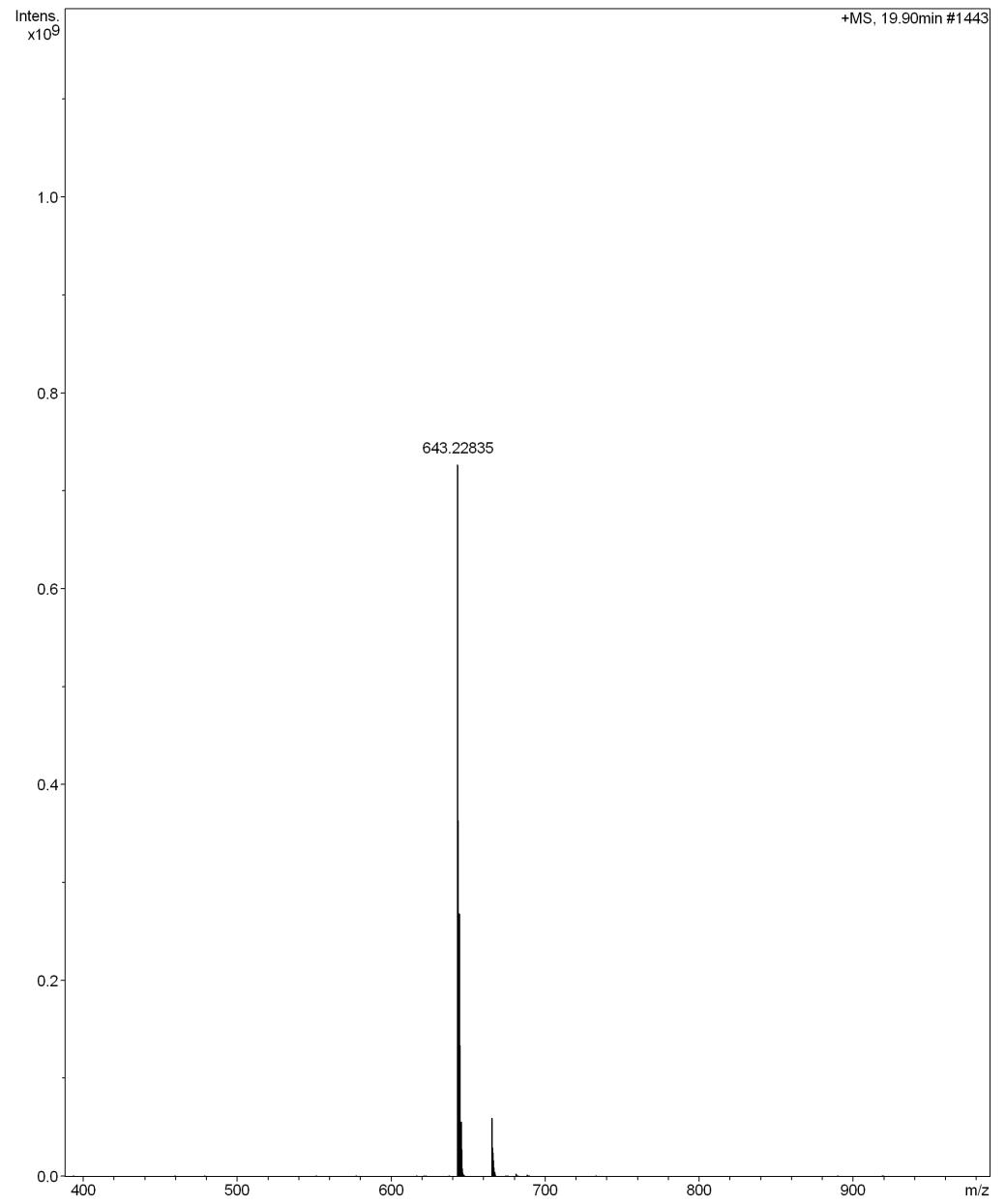


**Fig. S5.** Mass spectrometry of PD.


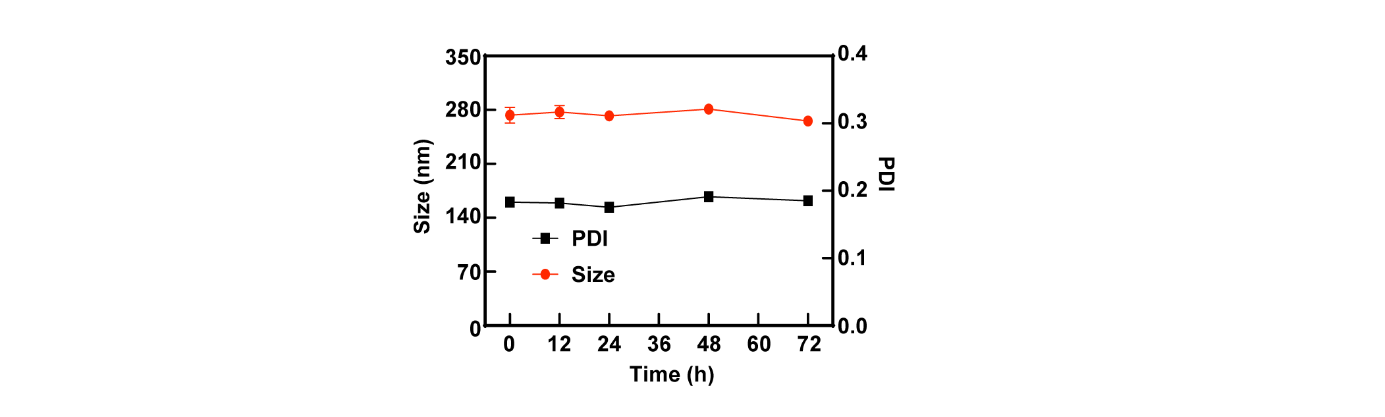


**Fig. S6. Hydrodynamic diameter and polydispersity index (PDI) of UPD nanoparticles over 72 hours of incubation in PBS.**


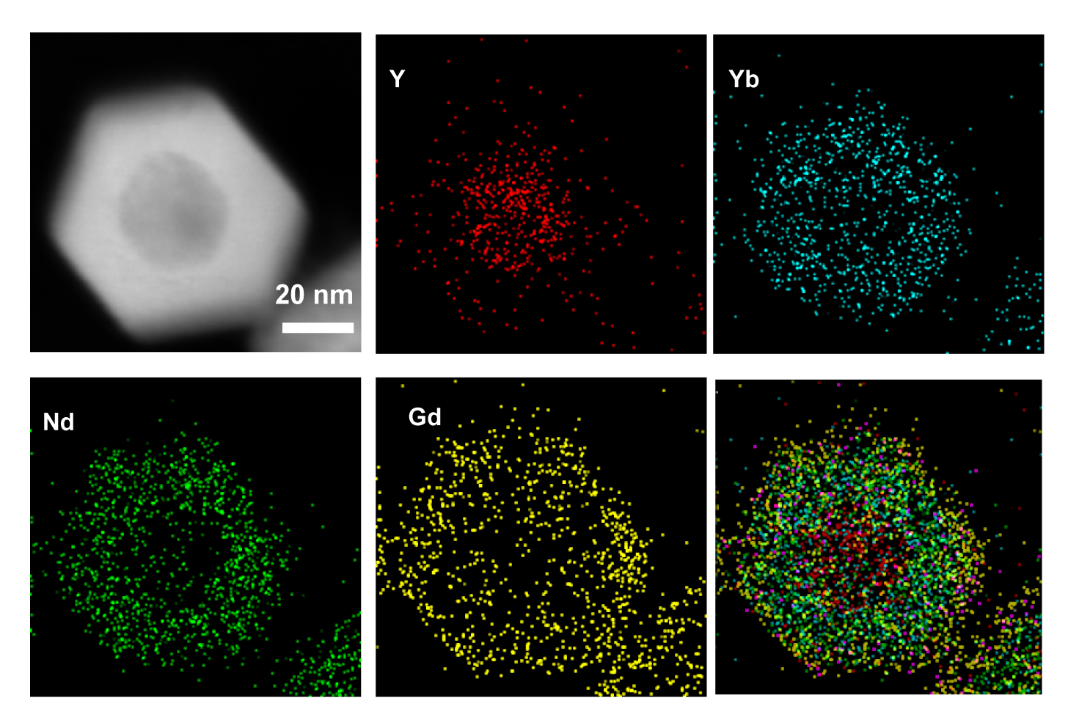


**Fig. S7.** The scanning transmission electron microscopic (STEM) image of UPD and the corresponding EDS elemental mapping of Y, Yb, Nd and Gd, respectively.


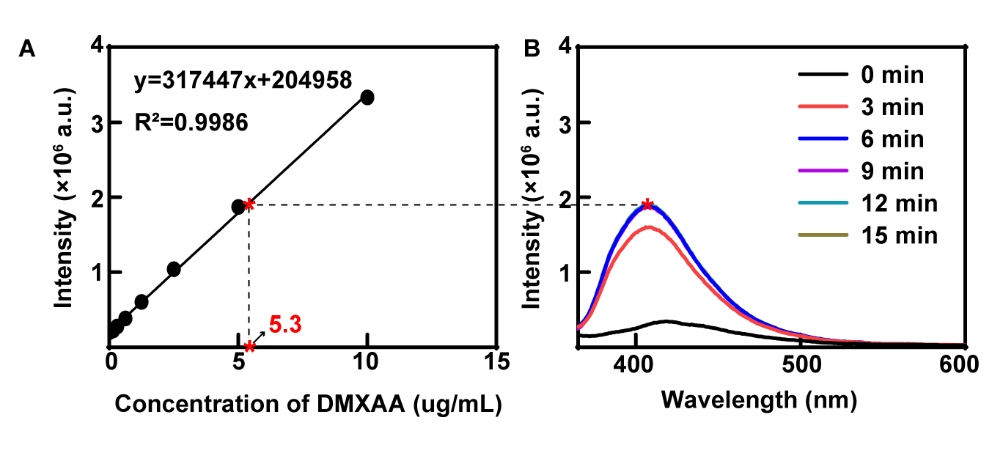


**Fig. S8.** A) The standard curve of DMXAA. ex=345 em=421 nm. B) The release of UPD under UV illumination at different time points. Experiments were repeated three times independently with similar results.


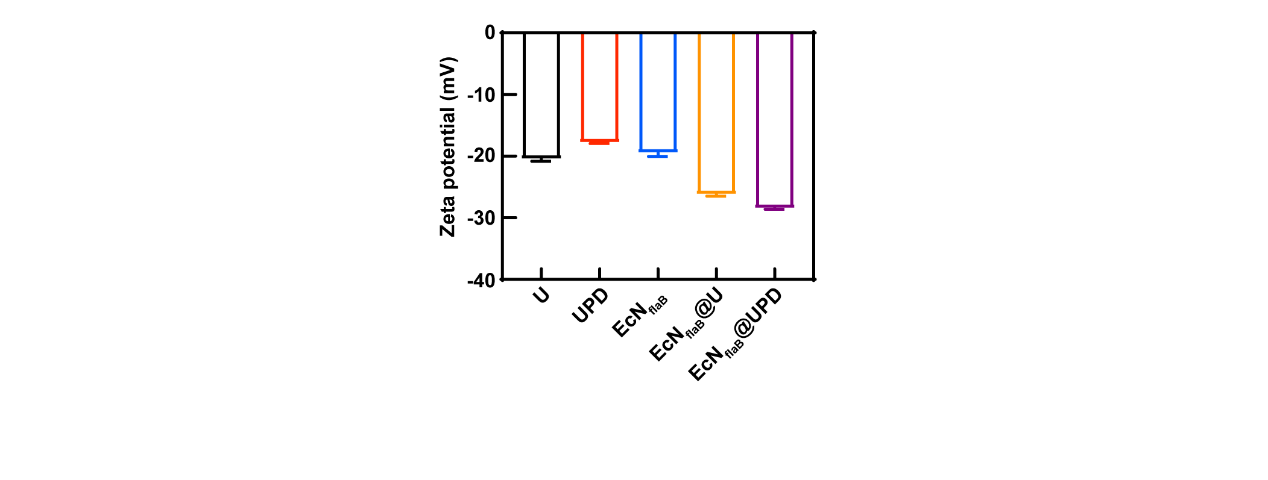


**Fig. S9.** Zeta potential of EcN_flaB_@UPD. (n=3 independent experiments)


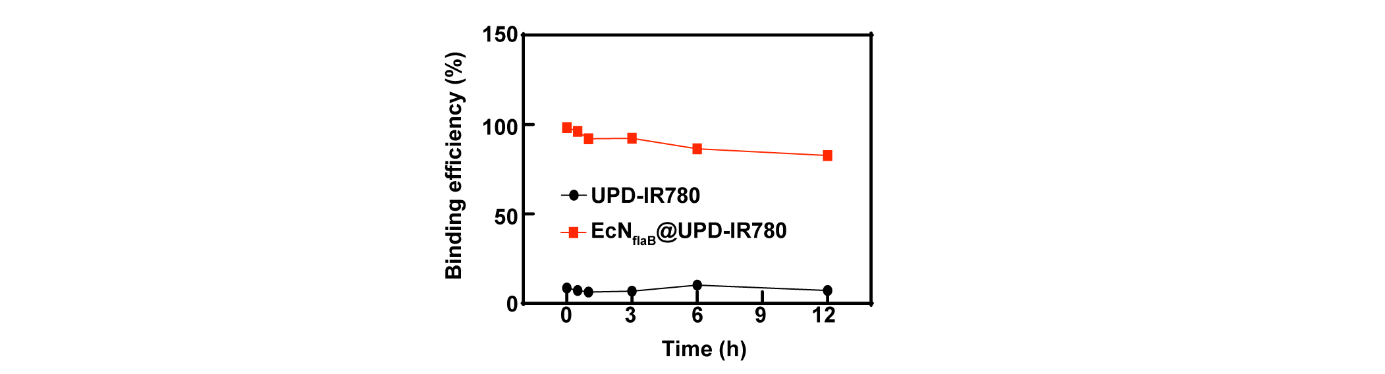


**Fig. S10.** Binding efficiency of EcN_flaB_@UPD-IR780 during incubation in 10% mouse serum at 37°C. All measurements were performed in triplicate at each time point.


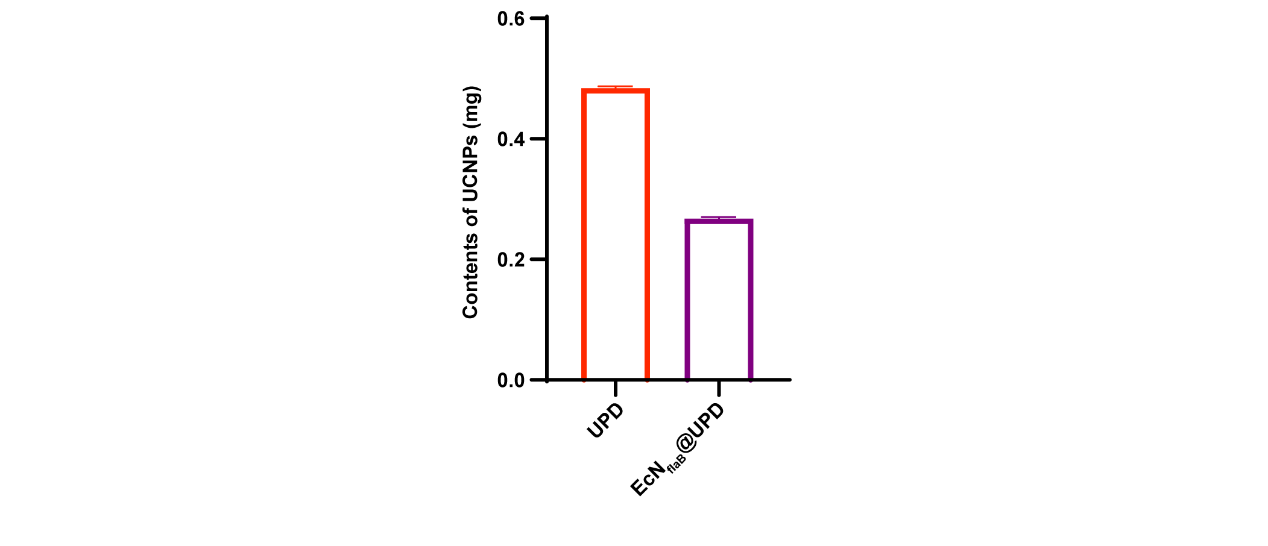


**Fig. S11.** The UCNPs contents of UPD (1 mg) and EcN_flaB_@UPD (10^7^ CFU) measured by ICP-MS. (n=3 independent experiments)


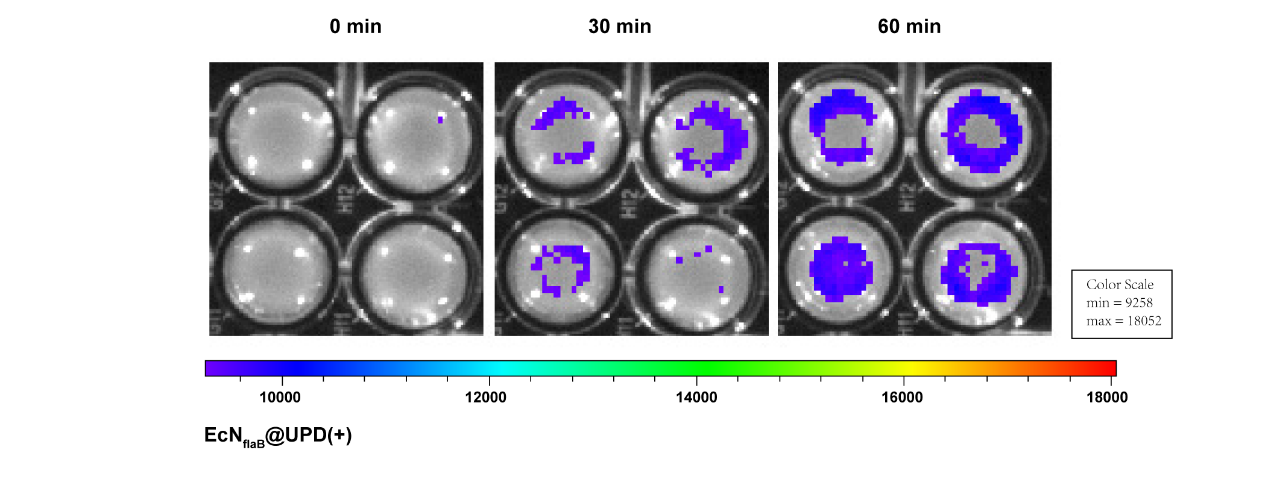


**Fig. S12.** IVIS images of EcN_flaB_@UPD(+) suspension under 808 nm exposure at different time points.


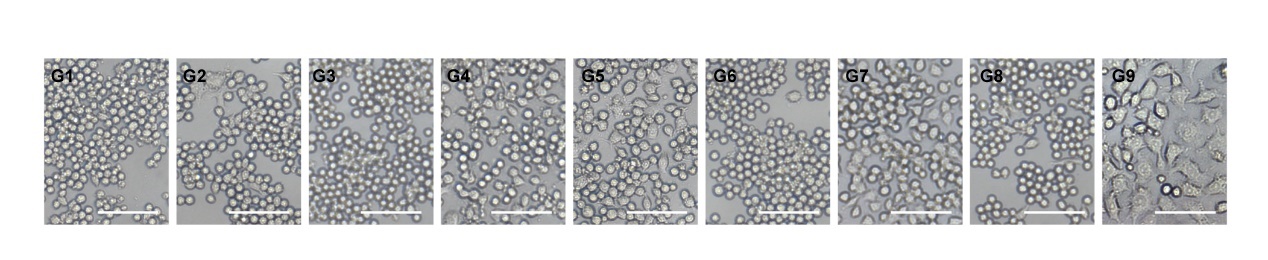


**Fig. S13.** Bright-field images of macrophages with M1 phenotype when M0 macrophages induced by EcN_flaB_@UPD(+). Scale bar=100 μm. G1-G9: PBS, DMXAA, UPD, UPD(+), EcN_flaB_, EcN_flaB_@U, EcN_flaB_@U(+), EcN_flaB_@UPD, and EcN_flaB_@UPD(+).


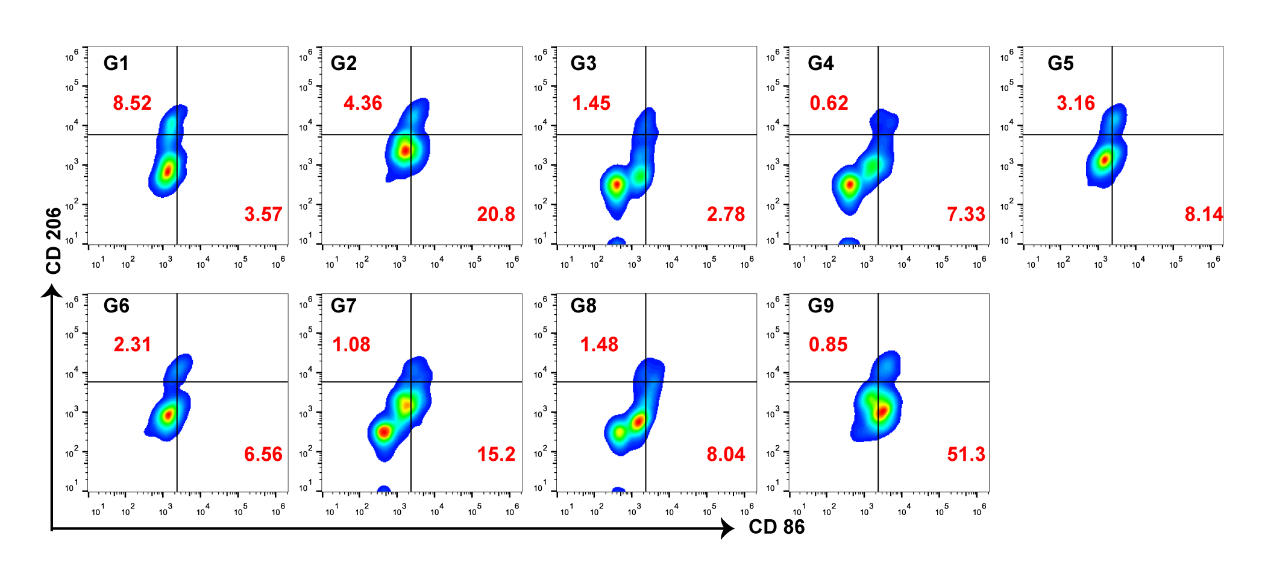


**Fig. S14.** Representative flow cytometry plots indicating CD86^+^ M1-like macrophages and CD206^+^ M2-like macrophages. G1-G9: PBS, DMXAA, UPD, UPD(+), EcN_flaB_, EcN_flaB_@U, EcN_flaB_@U(+), EcN_flaB_@UPD, and EcN_flaB_@UPD(+).


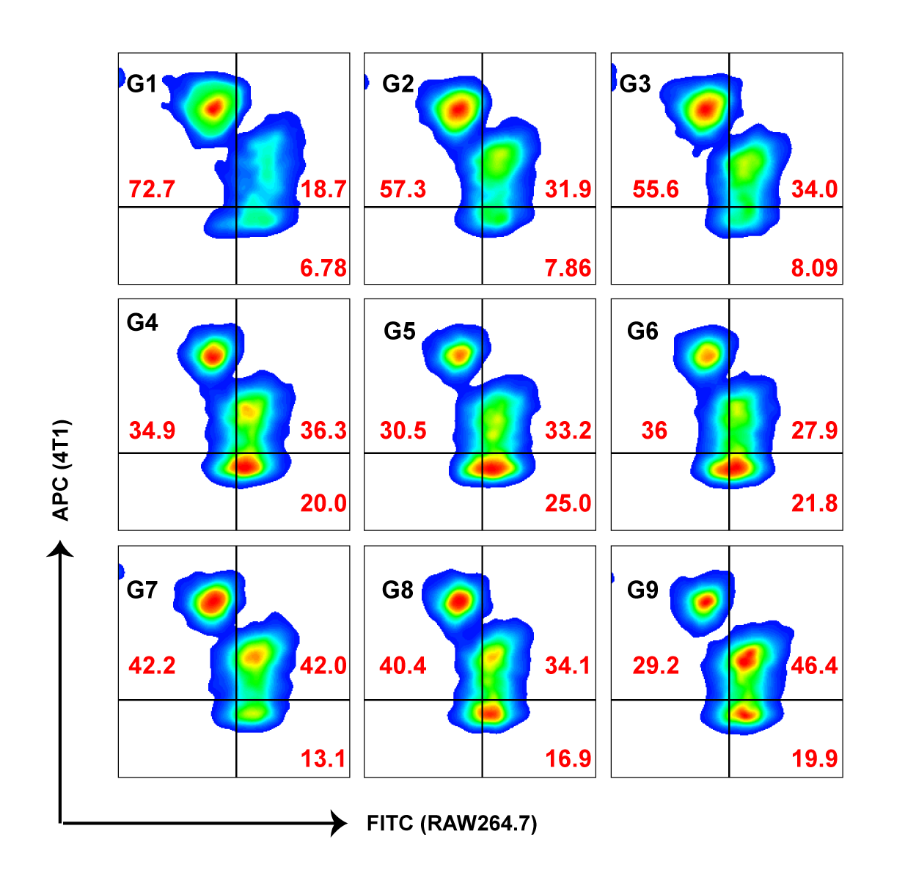


**Fig. S15.** Representative flow cytometric analysis of 4T1 cell phagocytosis by RAW264.7 after treatment with different formulations. G1-G9: PBS, DMXAA, UPD, UPD(+), EcN_flaB_, EcN_flaB_@U, EcN_flaB_@U(+), EcN_flaB_@UPD, and EcN_flaB_@UPD(+).


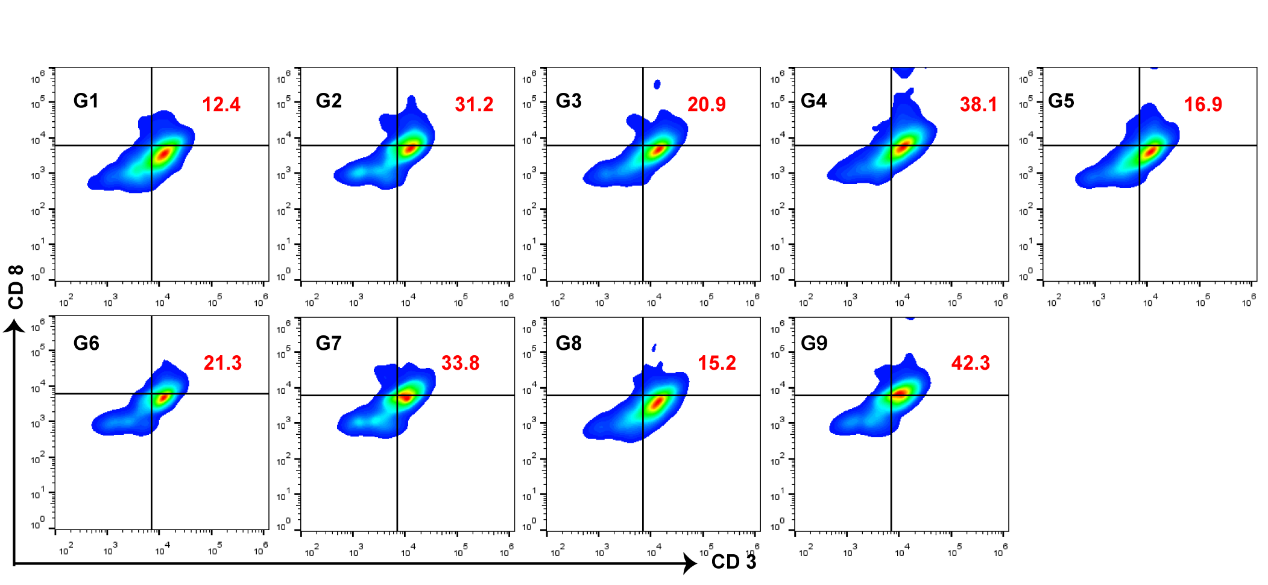


**Fig. S16.** Representative frequency of CD3^+^CD8^+^ T cells in splenic lymphocytes treated with macrophages. G1-G9: PBS, DMXAA, UPD, UPD(+), EcN_flaB_, EcN_flaB_@U, EcN_flaB_@U(+), EcN_flaB_@UPD, and EcN_flaB_@UPD(+).


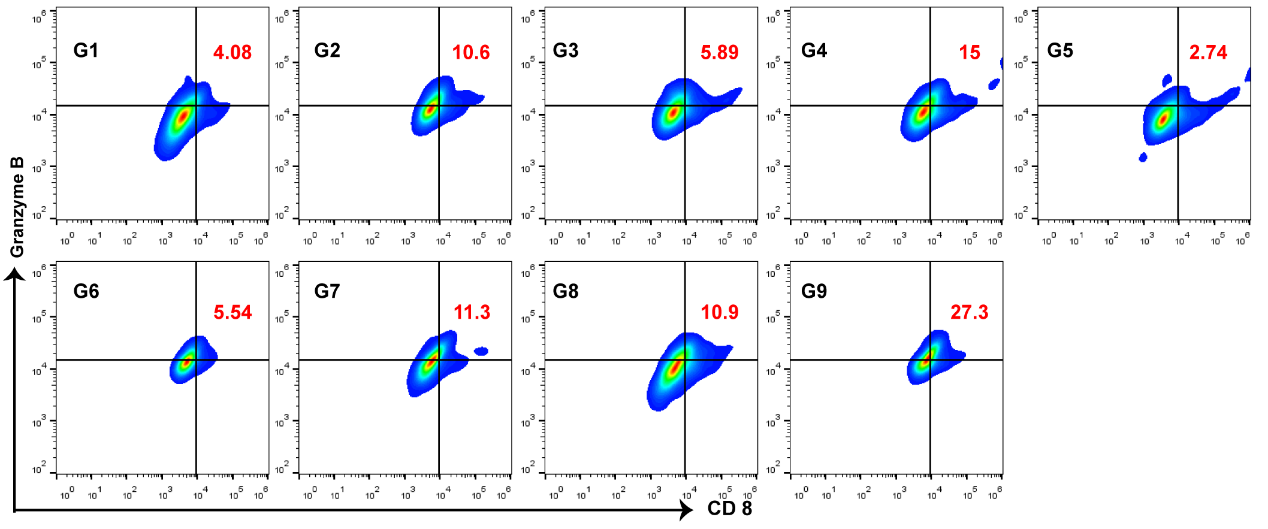


**Fig. S17.** Representative frequency of CD8^+^ Granzyme B^+^ T cells in CD3^+^ splenic lymphocytes treated with various macrophages. G1-G9: PBS, DMXAA, UPD, UPD(+), EcN_flaB_, EcN_flaB_@U, EcN_flaB_@U(+), EcN_flaB_@UPD, and EcN_flaB_@UPD(+).


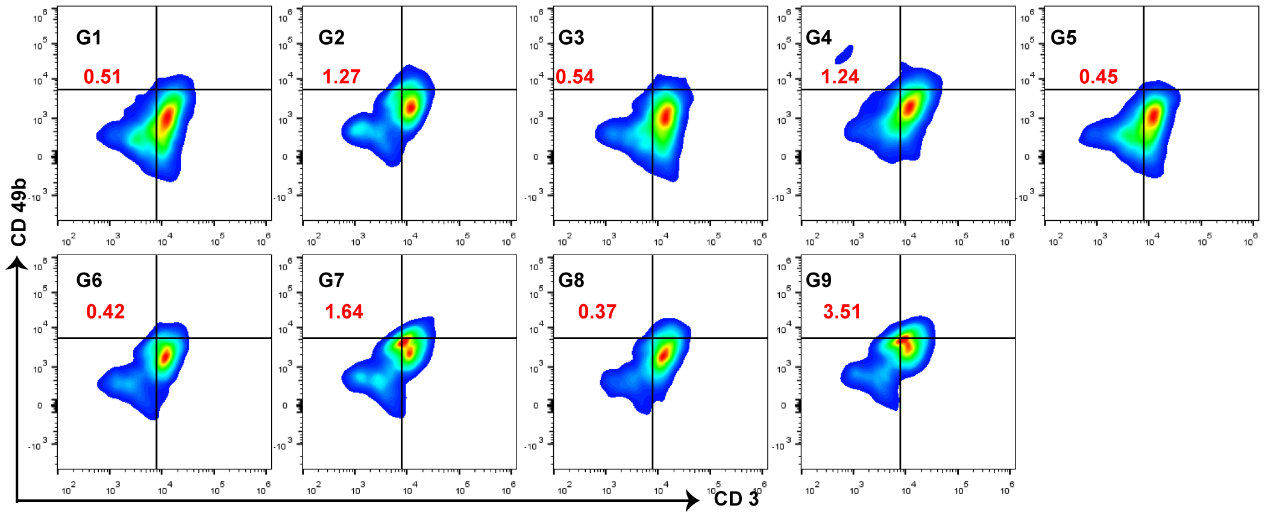


**Fig. S18.** Flow cytometric analysis of CD3^-^ CD49b^+^ cells in splenic lymphocytes treated with different macrophages. G1-G9: PBS, DMXAA, UPD, UPD(+), EcN_flaB_, EcN_flaB_@U, EcN_flaB_@U(+), EcN_flaB_@UPD, and EcN_flaB_@UPD(+).


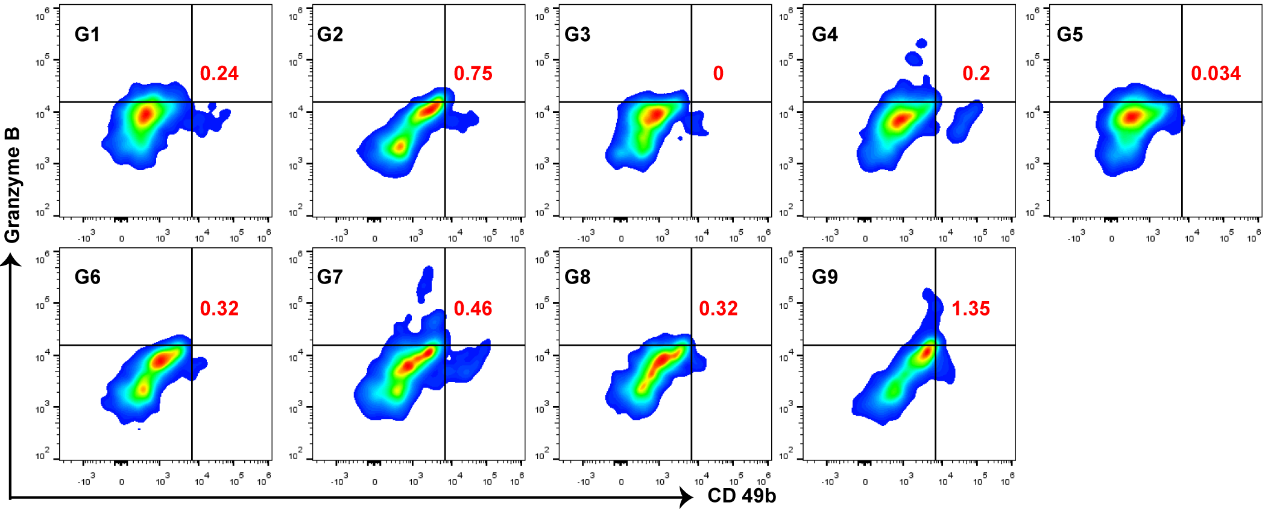


**Fig. S19.** Flow cytometric analysis of CD49b^+^ Granzyme B^+^ cells in CD3^-^ splenic lymphocytes treated with different macrophages. G1-G9: PBS, DMXAA, UPD, UPD(+), EcN_flaB_, EcN_flaB_@U, EcN_flaB_@U(+), EcN_flaB_@UPD, and EcN_flaB_@UPD(+).


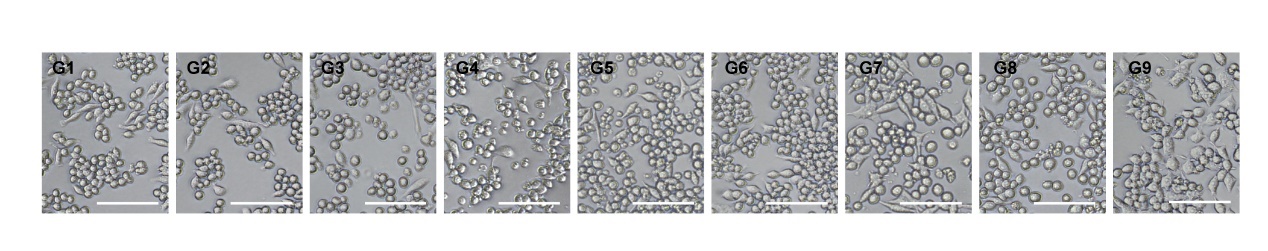


**Fig. S20.** Bright-field images of macrophages with M1 phenotype when M2 macrophages were induced by EcN_flaB_@UPD(+). Scale bar= 100 μm. G1-G9: PBS, DMXAA, UPD, UPD(+), EcN_flaB_, EcN_flaB_@U, EcN_flaB_@U(+), EcN_flaB_@UPD, and EcN_flaB_@UPD(+).


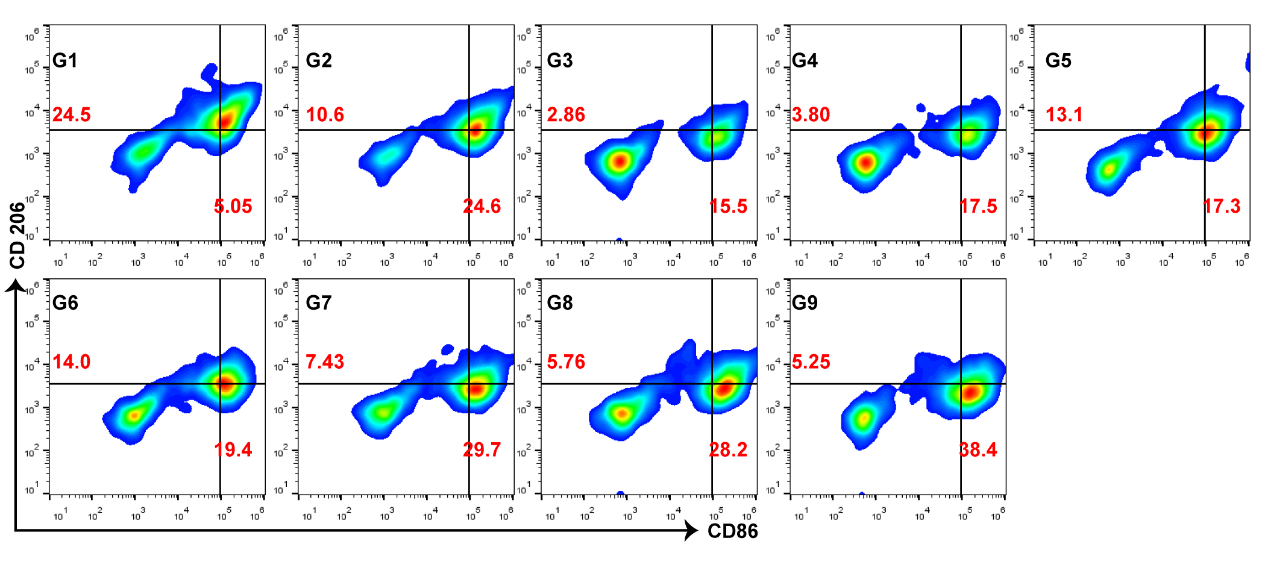


**Fig. S21.** Representative flow cytometry plots of CD86 or CD206-positive macrophages of RAW264.7 after different treatments at indicated time points. G1-G9: PBS, DMXAA, UPD, UPD(+), EcN_flaB_, EcN_flaB_@U, EcN_flaB_@U(+), EcN_flaB_@UPD, and EcN_flaB_@UPD(+).


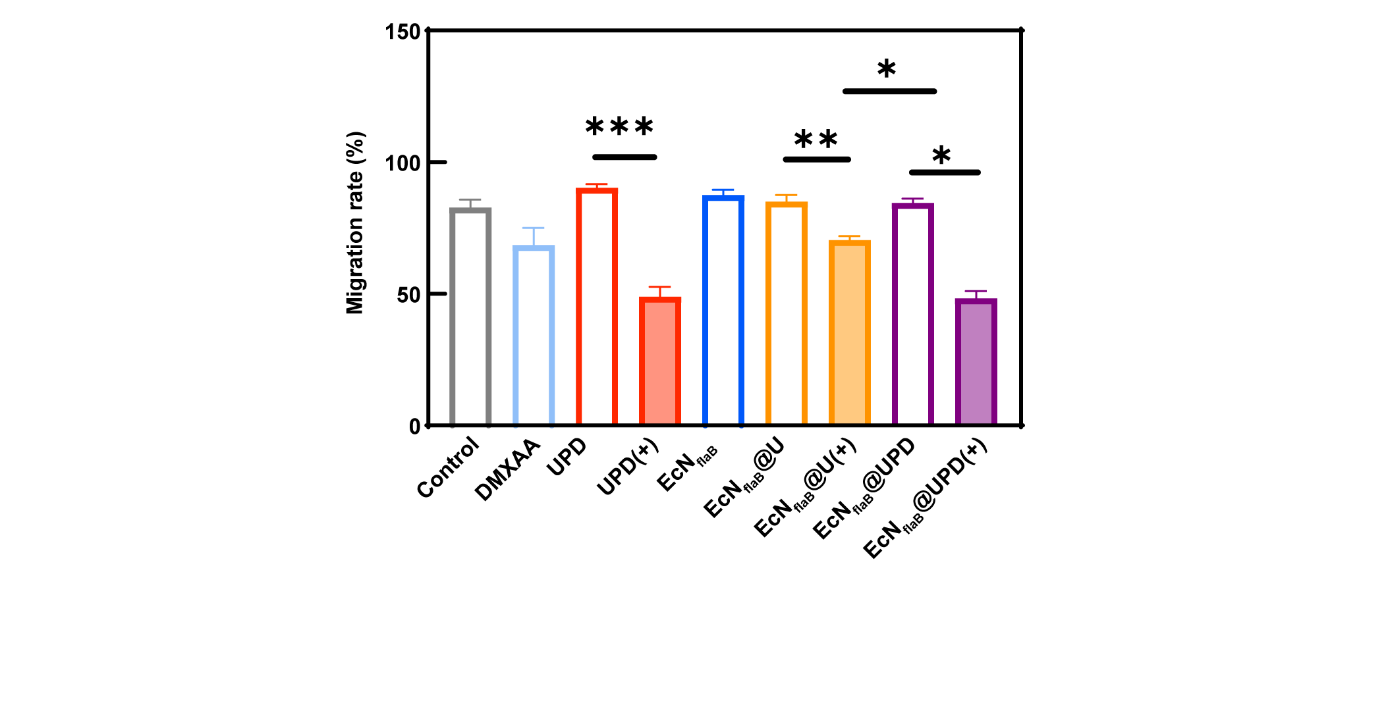


**Fig. S22.** Statistical analysis of wound area of 4T1 cells after treatments. (n=3)


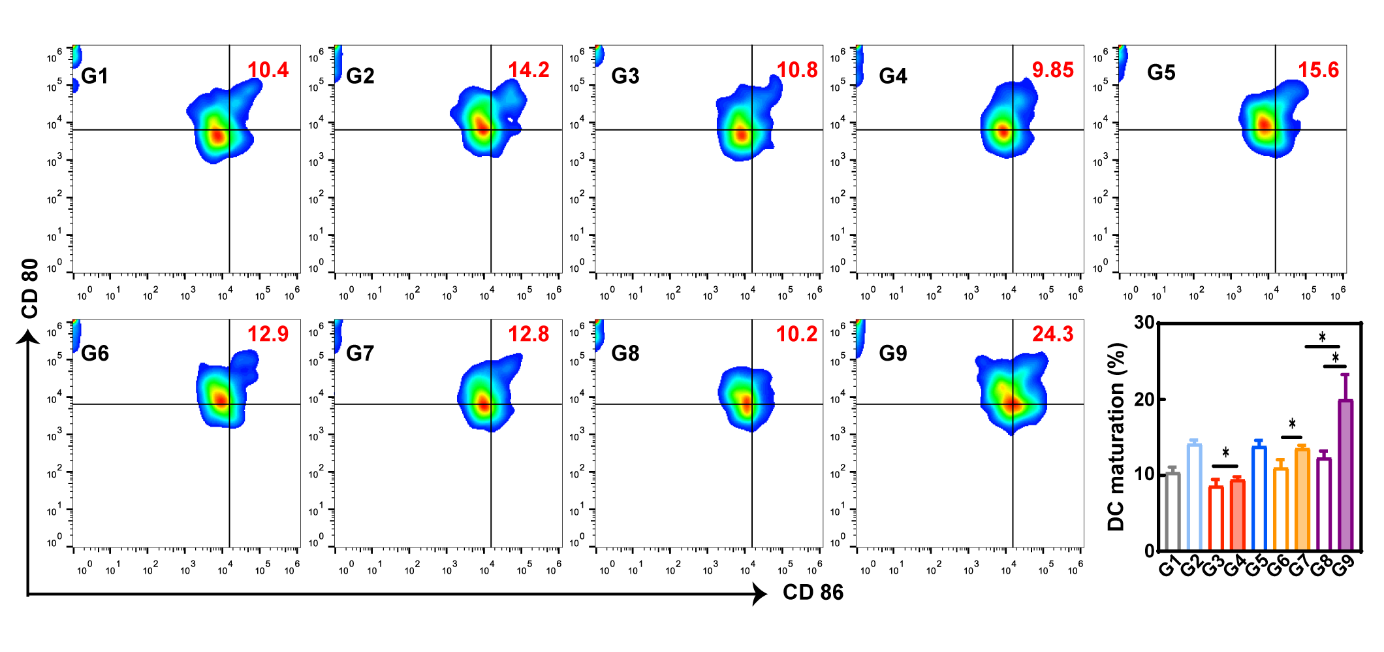


**Fig. S23.** Representative flow cytometry plots and quantifications of the percentages of mature DCs (CD11c^+^CD80^+^CD86^+^). G1-G9: PBS, DMXAA, UPD, UPD(+), EcN_flaB_, EcN_flaB_@U, EcN_flaB_@U(+), EcN_flaB_@UPD, and EcN_flaB_@UPD(+). Data are means ± SEM. Statistical analysis was performed using one-way analysis of variance (ANOVA) with Tukey’s post-test. *P<0.05, **P<0.01, ***P<0.001.


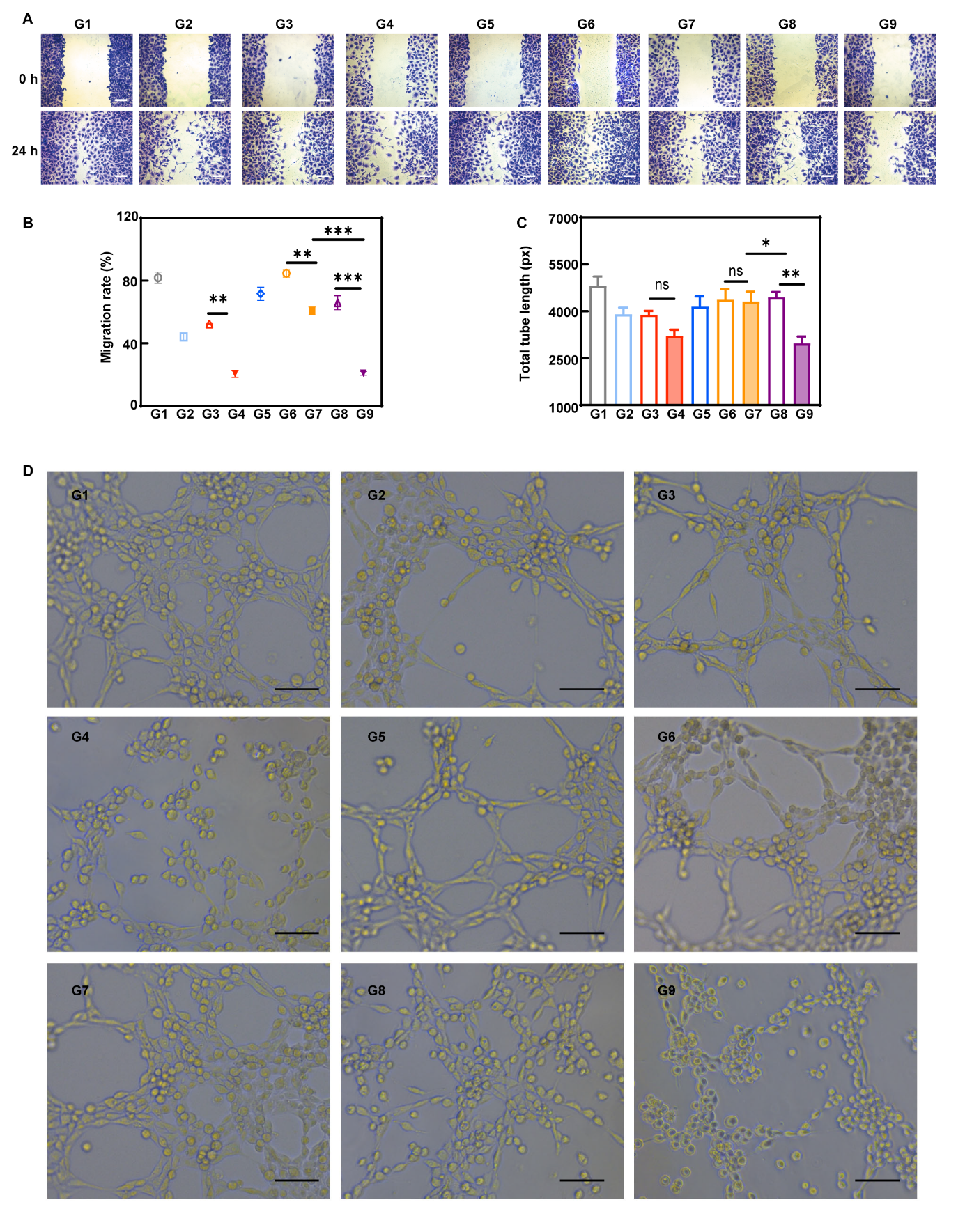


**Fig. S24.** A) HUVEC cells wound closure and cell migration assay after different treatments with supernatant of G1-G9: PBS, DMXAA, UPD, UPD(+), EcN_flaB_, EcN_flaB_@U, EcN_flaB_@U(+), EcN_flaB_@UPD, and EcN_flaB_@UPD(+) after incubation for 0 and 24 h. Scale bar = 100 μm. B) Statistical analysis of wound area of HUVEC. Analysis of tube formation by HUVECs treated with the supernatant of various materials. C) Length of formed tubes (measured in px). D) Brightfield microscopy images. Scale bar = 100 μm. Data are means ± SEM. Statistical analysis was performed using one-way analysis of variance (ANOVA) with Tukey’s post-test. *P<0.05, **P<0.01, ***P<0.001


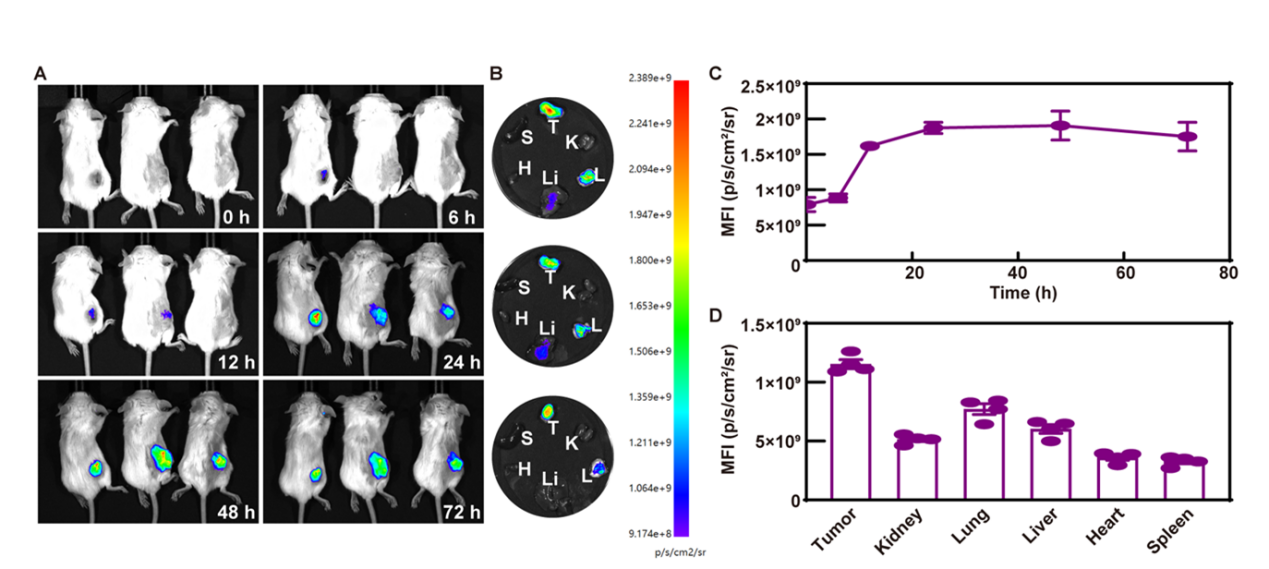


**Fig. S25.** Tumor targeting ability on 4T1 subcutaneous tumor. A) IVIS images of 4T1 tumor-bearing mice at 0, 6, 12, 24, 48 and 72 hours after tail vein injection with IR780-EcN_flaB_@UPD. B) IVIS images of heart, liver, spleen, lung, kidney, and tumor tissues sampled at 72 hours post-injection. C) Radiant efficiencies of the tumors. D) Radiant efficiencies of the tissues harvested at 72^nd^ hour.


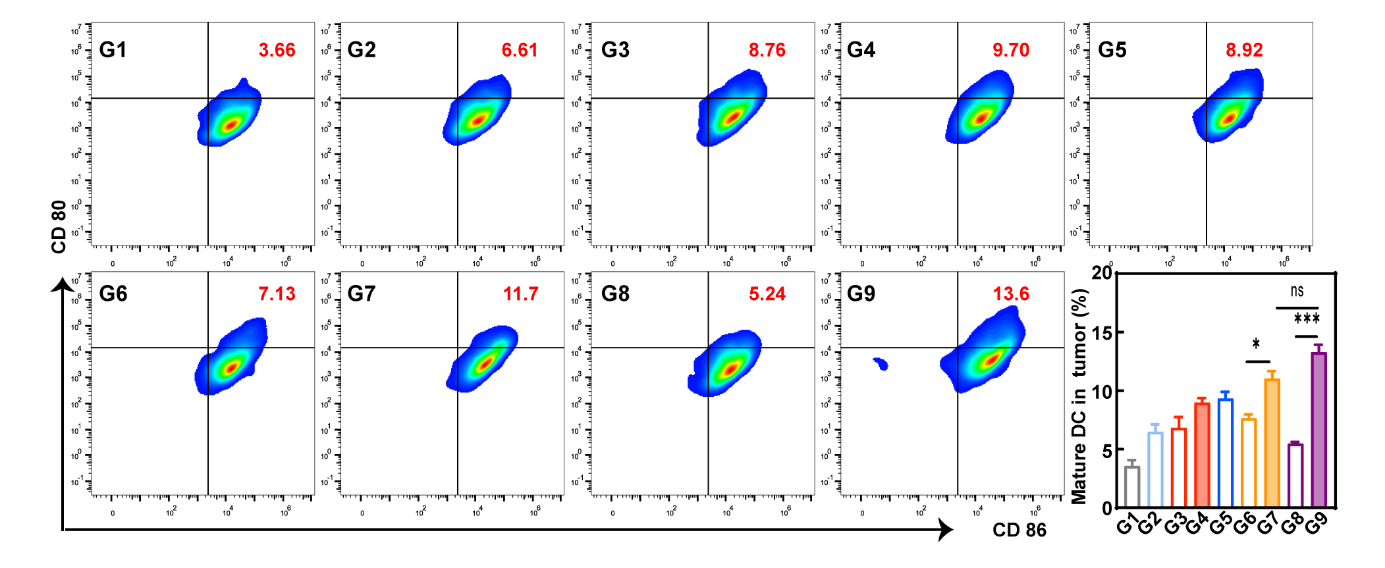


**Fig. S26.** Representative flow cytometry plots indicating mature DCs (CD80^+^ CD86^+^) in tumors and their quantitative analysis. (n=4) G1-G9: PBS, DMXAA, UPD, UPD(+), EcN_flaB_, EcN_flaB_@U, EcN_flaB_@U(+), EcN_flaB_@UPD, and EcN_flaB_@UPD(+). Data are means ± SEM. Statistical analysis was performed using one-way analysis of variance (ANOVA) with Tukey’s post-test. *P<0.05, **P<0.01, ***P<0.001.


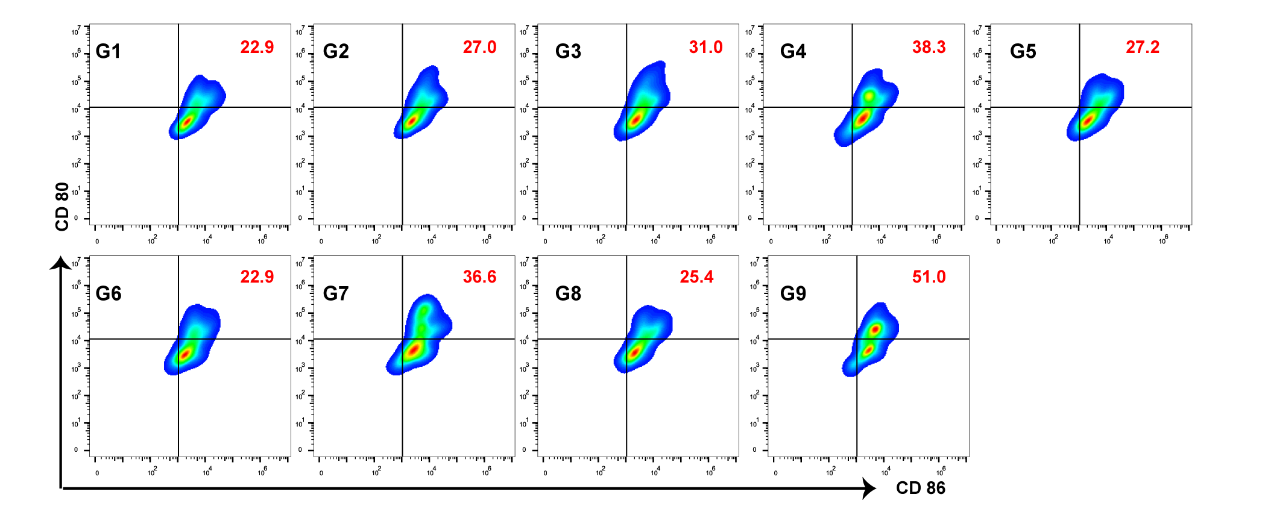


**Fig. S27.** Representative flow cytometry plots indicating mature DCs (CD80^+^ CD86^+^) in TDLNs. G1-G9: PBS, DMXAA, UPD, UPD(+), EcN_flaB_, EcN_flaB_@U, EcN_flaB_@U(+), EcN_flaB_@UPD, and EcN_flaB_@UPD(+).


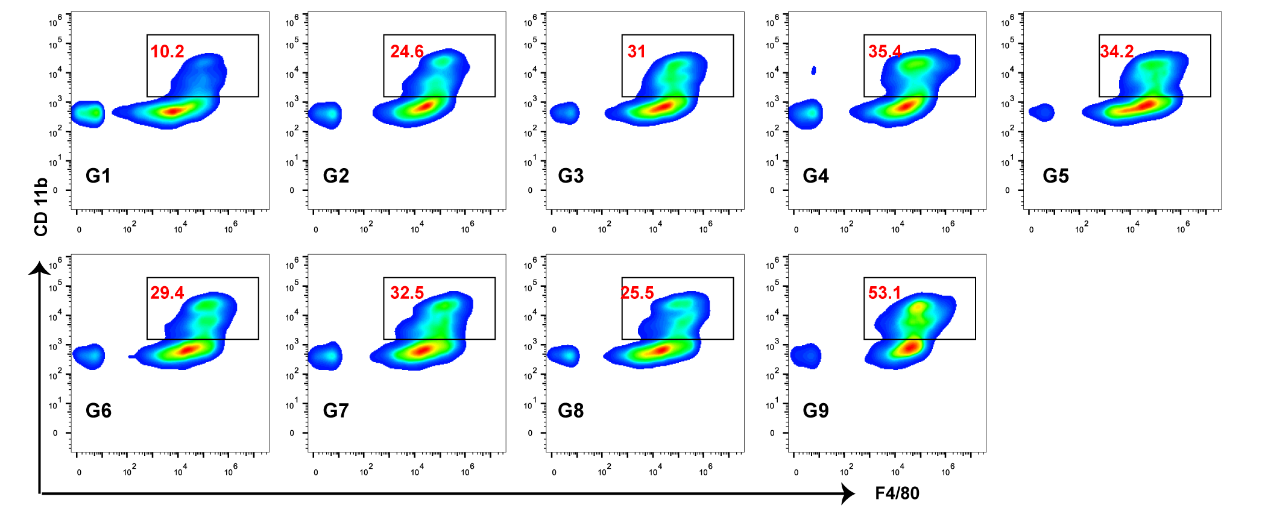


**Fig. S28.** Representative frequency of F4/80^+^CD11b^+^ cells in tumors. G1-G9: PBS, DMXAA, UPD, UPD(+), EcN_flaB_, EcN_flaB_@U, EcN_flaB_@U(+), EcN_flaB_@UPD, and EcN_flaB_@UPD(+).


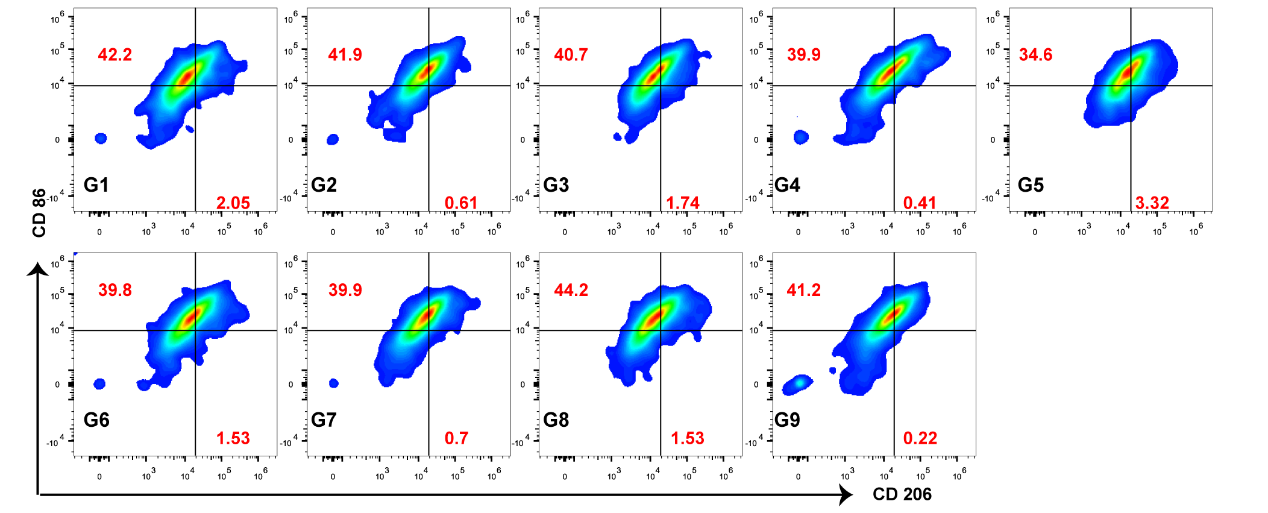


**Fig. S29.** Representative flow cytometry plots indicating M1-like macrophages (CD86^+^) and M2-like macrophages (CD206^+^) in tumors. G1-G9: PBS, DMXAA, UPD, UPD(+), EcN_flaB_, EcN_flaB_@U, EcN_flaB_@U(+), EcN_flaB_@UPD, and EcN_flaB_@UPD(+).


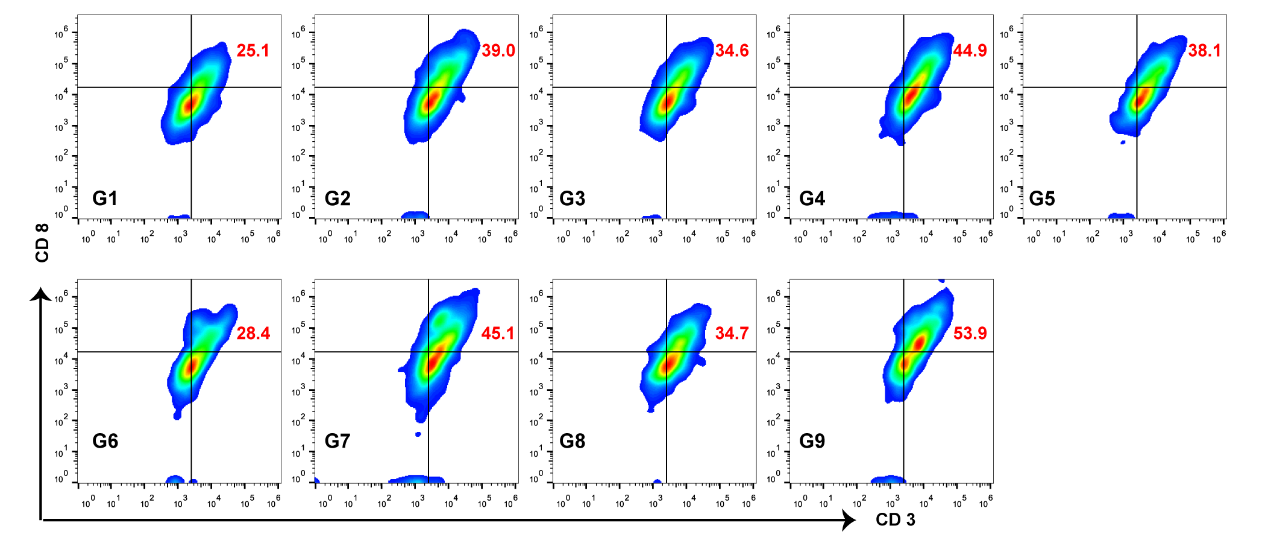


**Fig. S30.** Representative flow cytometry plots of CD3^+^CD8⁺ T cells in tumors. G1-G9: PBS, DMXAA, UPD, UPD(+), EcN_flaB_, EcN_flaB_@U, EcN_flaB_@U(+), EcN_flaB_@UPD, and EcN_flaB_@UPD(+).


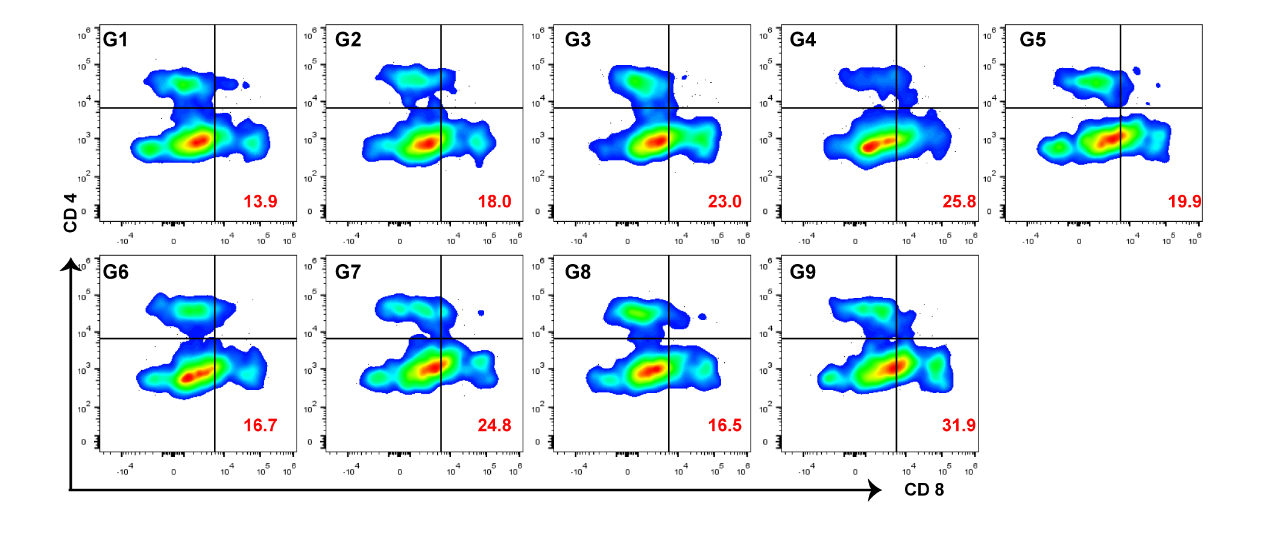


**Fig. S31.** Representative flow cytometry plots of CD8⁺ T-cells among CD3⁺T-cells in spleen. G1-G9: PBS, DMXAA, UPD, UPD(+), EcN_flaB_, EcN_flaB_@U, EcN_flaB_@U(+), EcN_flaB_@UPD, and EcN_flaB_@UPD(+).


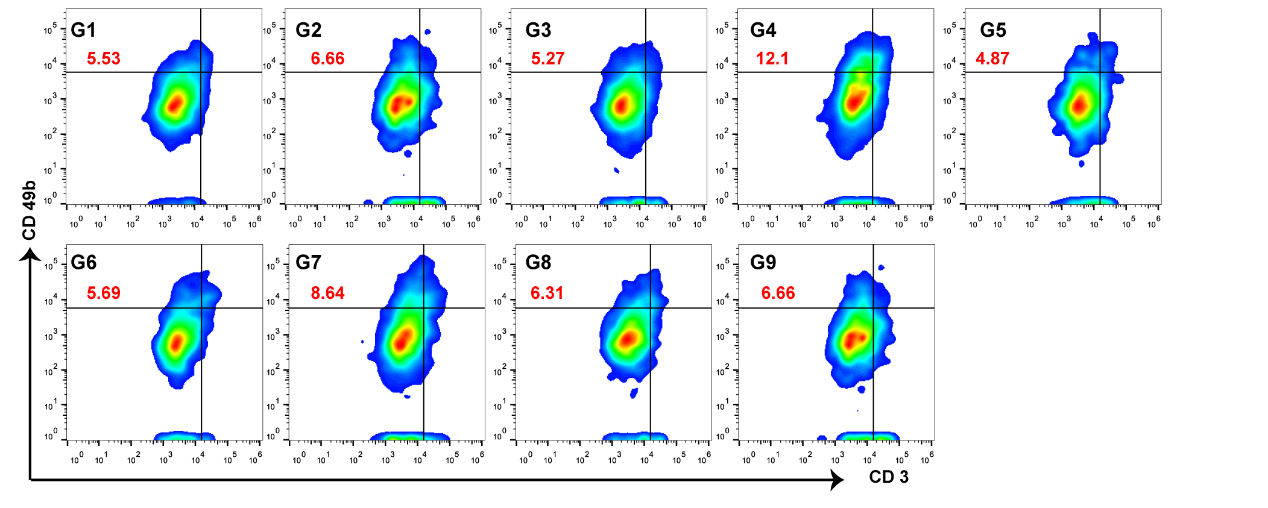


**Fig. S32.** Representative plots show expression of CD3 and CD49b in CD45^+^ cells. G1-G9: PBS, DMXAA, UPD, UPD(+), EcN_flaB_, EcN_flaB_@U, EcN_flaB_@U(+), EcN_flaB_@UPD, and EcN_flaB_@UPD(+).


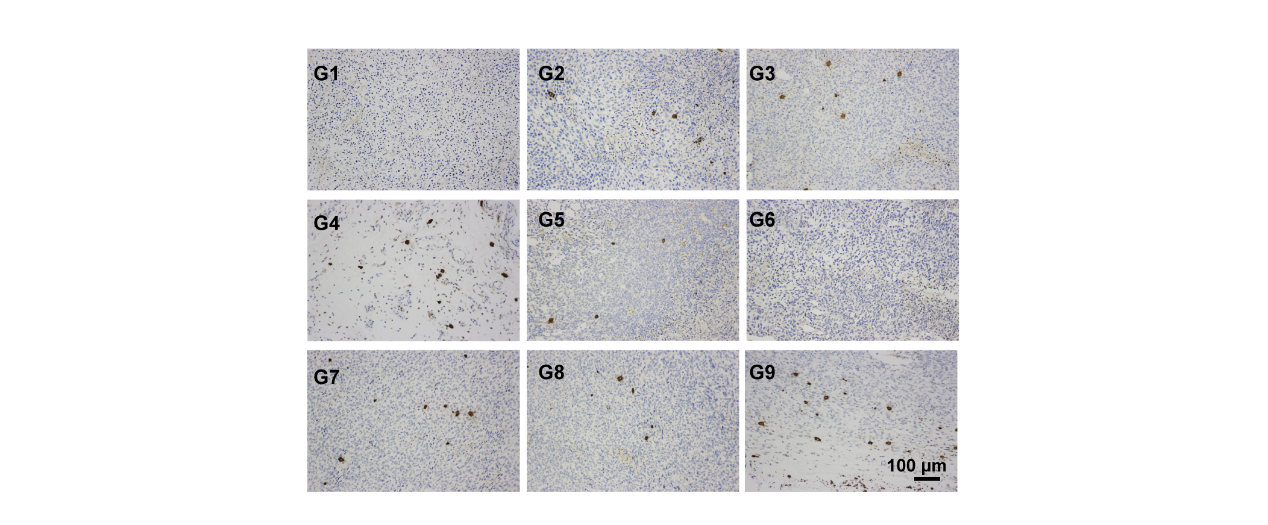


**Fig. S33.** Immunohistochemical (IHC) analysis of granzyme B in tumor sections. G1-G9: PBS, DMXAA, UPD, UPD(+), EcN_flaB_, EcN_flaB_@U, EcN_flaB_@U(+), EcN_flaB_@UPD, and EcN_flaB_@UPD(+).


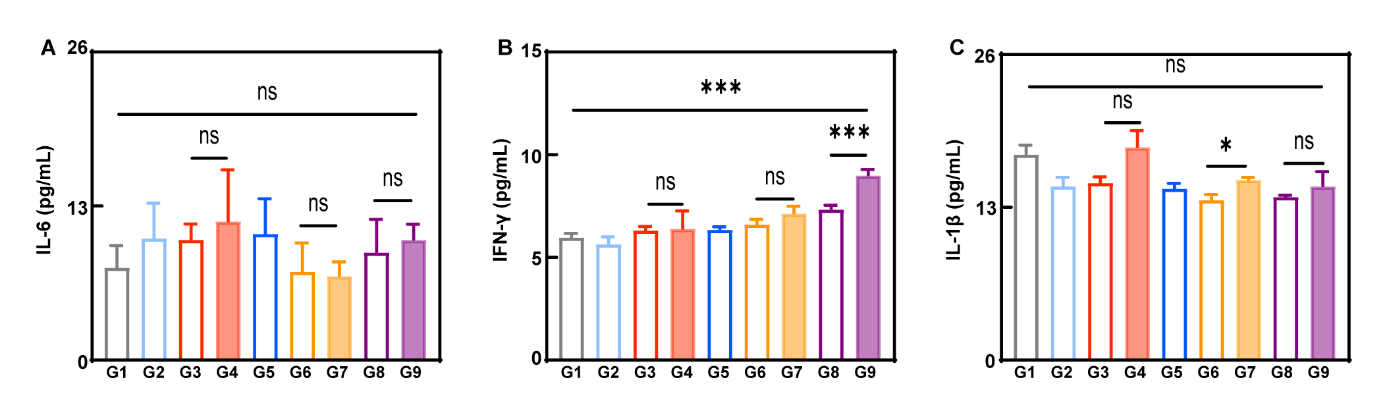


**Fig. S34.** Serum levels of A) IL-6, B) IFN-γ and C) IL-1β from mice isolated at day 17 after different treatments. (n = 3 animals per group) G1-G9: PBS, DMXAA, UPD, UPD(+), EcN_flaB_, EcN_flaB_@U, EcN_flaB_@U(+), EcN_flaB_@UPD, and EcN_flaB_@UPD(+).


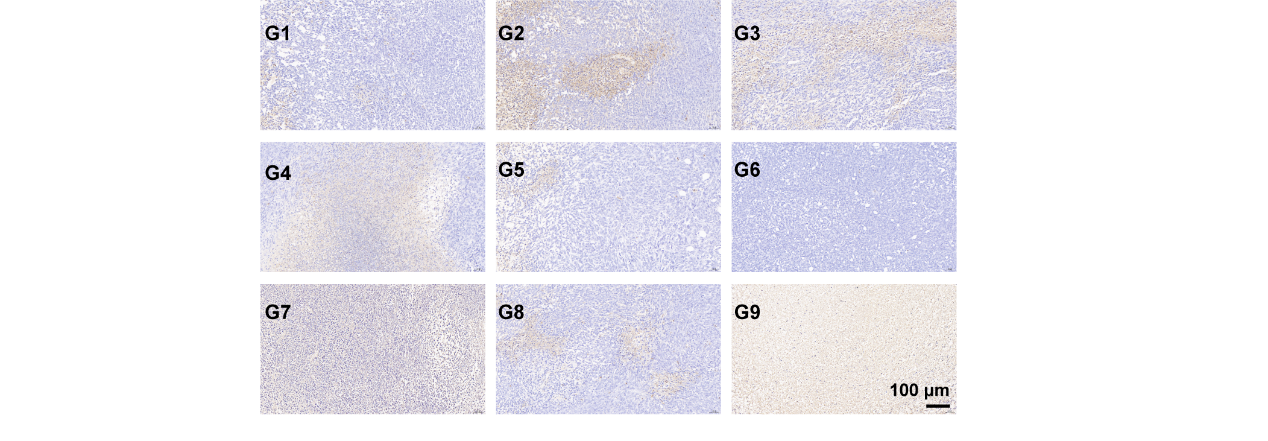


**Fig. S35.** TUNEL-stained tumor sections. G1-G9: PBS, DMXAA, UPD, UPD(+), EcN_flaB_, EcN_flaB_@U, EcN_flaB_@U(+), EcN_flaB_@UPD, and EcN_flaB_@UPD(+).


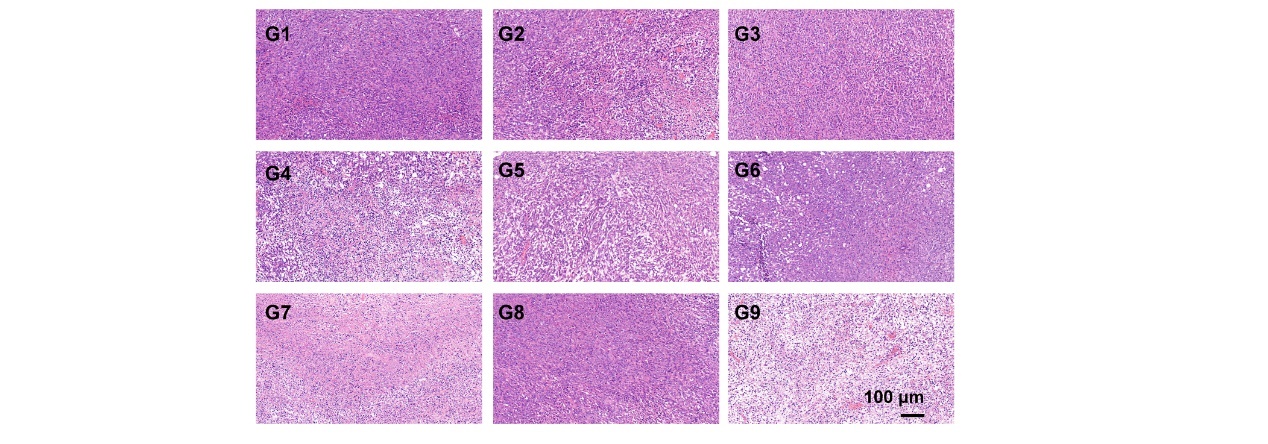


**Fig. S36.** H&E-stained tumor sections. G1-G9: PBS, DMXAA, UPD, UPD(+), EcN_flaB_, EcN_flaB_@U, EcN_flaB_@U(+), EcN_flaB_@UPD, and EcN_flaB_@UPD(+).


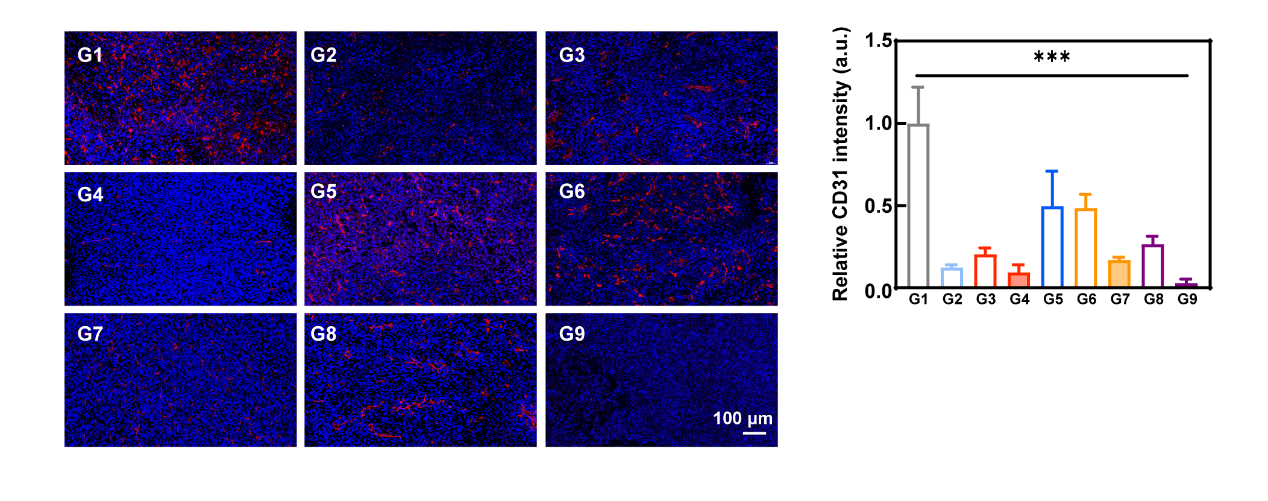


**Fig. S37.** Immunostaining of CD31^+^ in tumor sections. G1-G9: PBS, DMXAA, UPD, UPD(+), EcN_flaB_, EcN_flaB_@U, EcN_flaB_@U(+), EcN_flaB_@UPD, and EcN_flaB_@UPD(+).


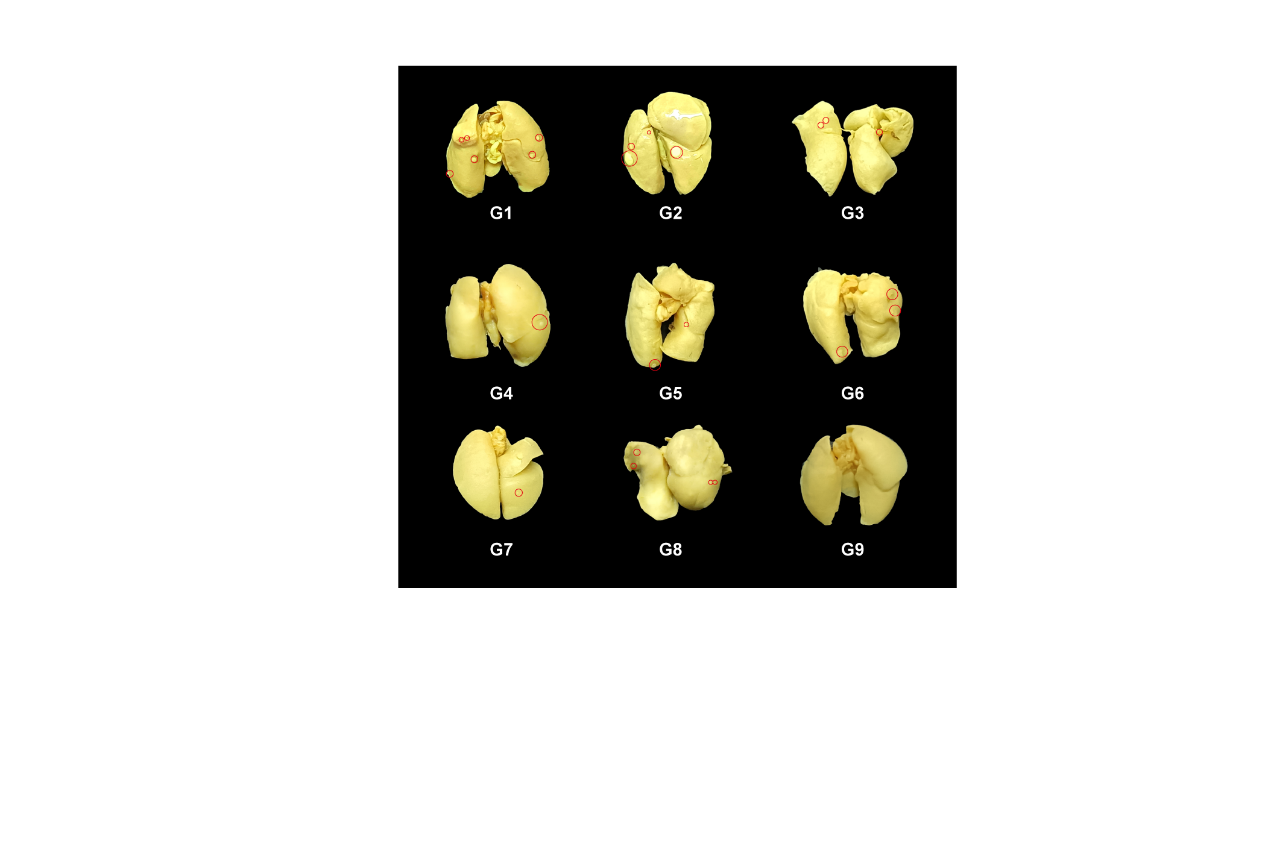


**Fig. S38.** The representative Lungs on the 21^st^-day post-treatments. G1-G9: PBS, DMXAA, UPD, UPD(+), EcN_flaB_, EcN_flaB_@U, EcN_flaB_@U(+), EcN_flaB_@UPD, and EcN_flaB_@UPD(+).


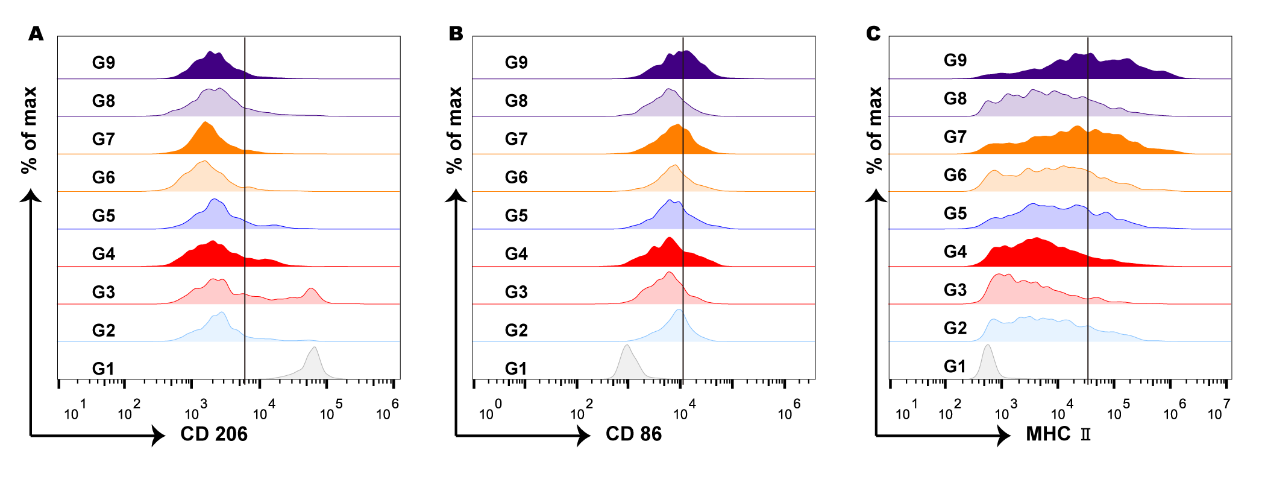


**Fig. S****39.** Representative histograms from flow cytometric analyses, showing the expression of A) CD206, B) CD86, and C) MHC II as macrophage markers in the recurrence tumors. Cells were pre-gated from F4/80^+^CD11b^+^ cells. G1-G9: PBS, DMXAA, UPD, UPD(+), EcN_flaB_, EcN_flaB_@U, EcN_flaB_@U(+), EcN_flaB_@UPD, and EcN_flaB_@UPD(+).


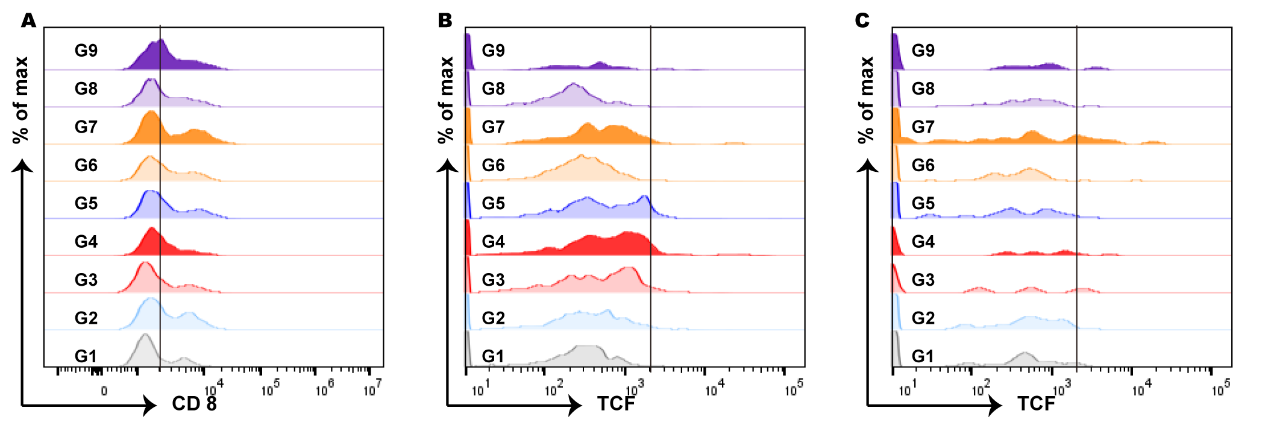


**Fig. S40.** T cell stemness induced by EcN_flaB_@UPD(+). The representative histogram shows A) CD8 expression in CD3^+^ tumor cells. B) TCF-1 expression in PD1^+^CD8^+^ CD3^+^CD45^+^ T cells in the tumor. C) TCF-1 expression in PD1^+^CD44^+^CD8^+^CD3^+^CD45^+^ T cells in the tumor. G1-G9: PBS, DMXAA, UPD, UPD(+), EcN_flaB_, EcN_flaB_@U, EcN_flaB_@U(+), EcN_flaB_@UPD, and EcN_flaB_@UPD(+).


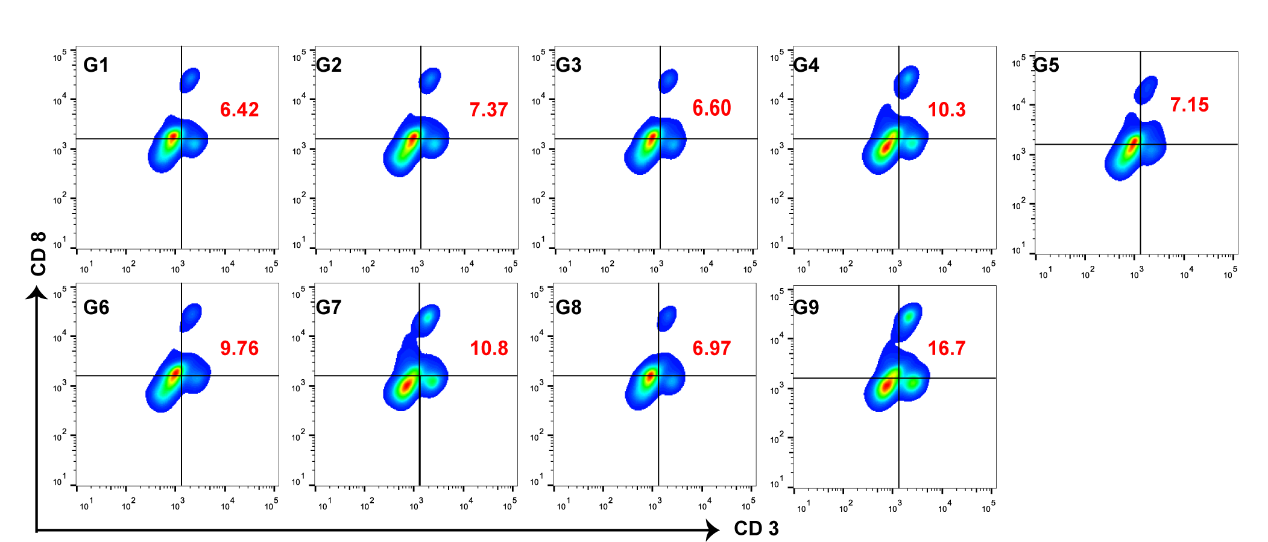


**Fig. S41.** Representative flow cytometry plots indicating CD3^+^ CD8^+^ T cells in spleens. G1-G9: PBS, DMXAA, UPD, UPD(+), EcN_flaB_, EcN_flaB_@U, EcN_flaB_@U(+), EcN_flaB_@UPD, and EcN_flaB_@UPD(+).


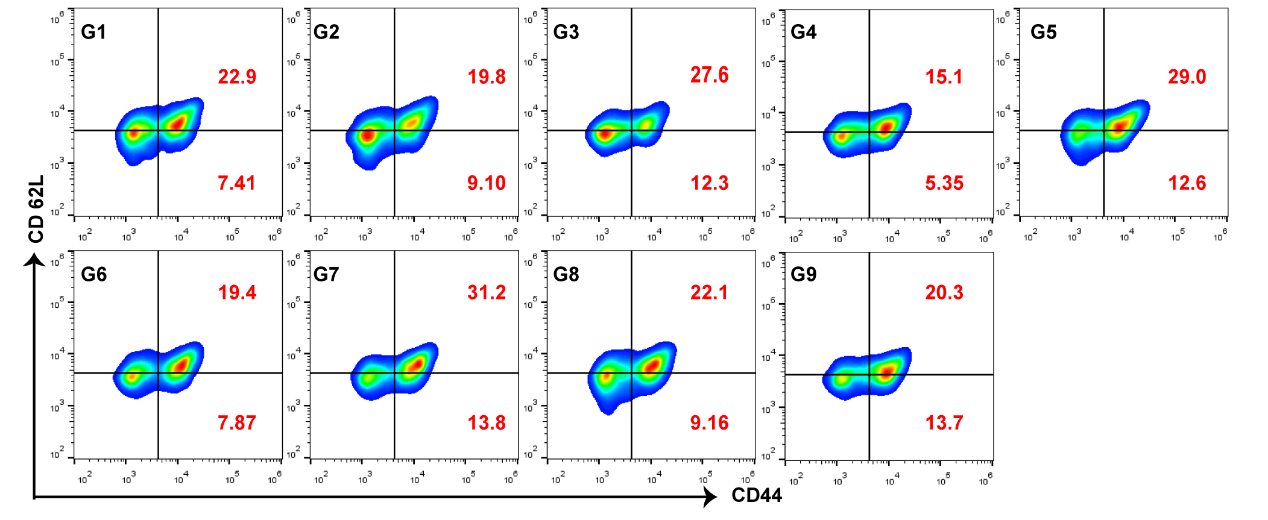


**Fig. S42.** Representative flow cytometric analysis of TEM (CD44^+^CD62L^-^) and TCM (CD44^+^ CD62L^+^) cells gating on CD8^+^ T cells in spleen of 4T1 tumor-bearing mice at the end of treatments. G1-G9: PBS, DMXAA, UPD, UPD(+), EcN_flaB_, EcN_flaB_@U, EcN_flaB_@U(+), EcN_flaB_@UPD, and EcN_flaB_@UPD(+).


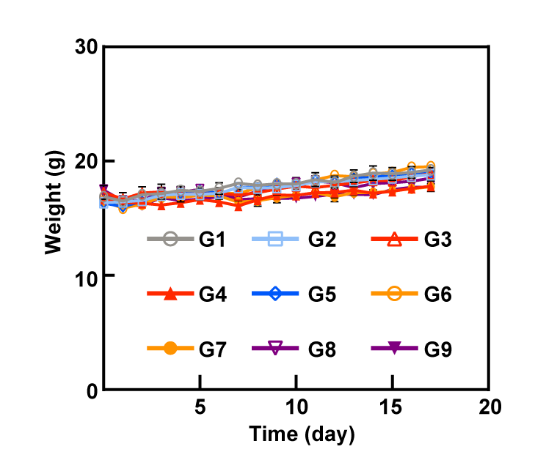


**Fig. S43.** Body weight of mice at different time points. G1-G9: PBS, DMXAA, UPD, UPD(+), EcN_flaB_, EcN_flaB_@U, EcN_flaB_@U(+), EcN_flaB_@UPD, and EcN_flaB_@UPD(+).

| **Gene** | **Forward primer (5’-3’)** | **Reverse primer (3’-5’)** |
| --- | --- | --- |
| **GAPDH** | GTTCCTACCCCCAATGTGTCC | TAGCCCAAGATGCCCTTCAGT |
| **TNF-α** | GACCCTCACACTCAGATCATCTTCT | CCACTTGGTGGTTTGCTACGA |
| **IFN-β** | CACAGCCCTCTCCATCAACTATAAG | GGCAGTGTAACTCTTCTGCATCTTC |
| **TLR5** | TGGGGACCCAGTATGCTAACT | CCACAGGAAAACAGCCGAAGT |
| **IL-6** | CCTGAGACTCAAGCAGAAATGG | AGAAGGAAGGTCGGCTTCAGT |
| **CXCL10** | CAACTGCATCCATATCGATGAC | GATTCCGGATTCAGACATCTCT |
| **Isg15** | AGCGAGCCTCTGAGCATCCTG | GCGTGTCTACAGTCTGCGTCAG |
| **NF-κB** | ATGGCAGACGATGATCCCTAC | CGGAATCGAAATCCCCTCTGTT |
| **MAPK1** | GGTTGTTCCCAAATGCTGACT | CAACTTCAATCCTCTTGTGAGGG |

**Table S1.** Primers used in this study.
